# Glucagon-like peptide-1 analogues: a new way to quit smoking? (SKIP)—a structured summary of a study protocol for a randomized controlled study

**DOI:** 10.1186/s13063-023-07164-9

**Published:** 2023-04-20

**Authors:** Sophia Lengsfeld, Thilo Burkard, Andrea Meienberg, Nica Jeanloz, David Coynel, Deborah R. Vogt, Lars G. Hemkens, Benjamin Speich, Davide Zanchi, Tobias E. Erlanger, Mirjam Christ-Crain, Bettina Winzeler

**Affiliations:** 1 Endocrinology, Diabetology and Metabolism, Department of Internal Medicine, University Hospital Basel, Petersgraben 4, 4031 Basel, Switzerland; 2 Medical Outpatient Department, University Hospital Basel, Petersgraben 4, Basel, 4031 Switzerland; 3 Department of Cardiology, University Hospital Basel, Basel, Switzerland; 4 Faculty of Medicine, University of Basel, Basel, Switzerland; 5 Division of Cognitive Neuroscience, Department of Psychology and Transfaculty Research Platform, University of Basel, Basel, Switzerland; 6 Department of Clinical Research, University of Basel and University Hospital of Basel, Basel, Switzerland; 7 Research Center for Clinical Neuroimmunology and Neuroscience Basel (RC2NB), University of Basel and University Hospital of Basel, Basel, Switzerland; 8 Meta-Research Innovation Center at Stanford (METRICS), Stanford University, Stanford, CA USA; 9 Meta-Research Innovation Center Berlin (METRIC-B), Berlin Institute of Health, Berlin, Germany; 10 Roche Innovation Centre Basel, F. Hoffmann- La Roche, Basel, Switzerland; 11 Stanford University Graduate School of Business, Stanford, CA USA

**Keywords:** Cigarette smoking, GLP-1 analogues, Dulaglutide, Weight gain, Prevalence abstinence rate, Diabetes, Nicotine craving, Nicotine addiction, Varenicline

## Abstract

**Background:**

Cigarette smoking is the leading preventable cause of premature death. Despite dedicated programmes, quit rates remain low due to barriers such as nicotine withdrawal syndrome or post-cessation weight gain. Glucagon-like peptide-1 (GLP-1) analogues reduce energy intake and body weight and seem to modulate addictive behaviour. These GLP-1 properties are of major interest in the context of smoking cessation. The aim of this study is to evaluate the GLP-1 analogue dulaglutide as a new therapy for smoking cessation.

**Methods:**

This is a placebo-controlled, double-blind, parallel group, superiority, single-centre randomized study including 255 patients. The intervention consists of a 12-week dulaglutide treatment phase with 1.5 mg once weekly or placebo subcutaneously, in addition to standard of care (behavioural counselling and pharmacotherapy with varenicline). A 40-week non-treatment phase follows. The primary outcome is the point prevalence abstinence rate at week 12. Smoking status is self-reported and biochemically confirmed by end-expiratory exhaled carbon monoxide measurement. Further endpoints include post-cessational weight gain, nicotine craving analysis, glucose homeostasis and long-term nicotine abstinence.

Two separate substudies assess behavioural, functional and structural changes by functional magnetic resonance imaging and measures of energy metabolism (i.e. resting energy expenditure, body composition).

**Discussion:**

Combining behavioural counselling and medical therapy, e.g. with varenicline, improves abstinence rates and is considered the standard of care. We expect a further increase in quit rates by adding a second component of medical therapy and assume a dual effect of dulaglutide treatment (blunting nicotine withdrawal symptoms *and* reducing post-cessational weight gain). This project is of high relevance as it explores novel treatment options aimed at preventing the disastrous consequences of nicotine consumption and obesity.

**Trial registration:**

ClinicalTrials.gov NCT03204396. Registered on June 26, 2017.

**Supplementary Information:**

The online version contains supplementary material available at 10.1186/s13063-023-07164-9.

## Administrative information

The protocol was developed in accordance with the recommendations for Interventional Trials (SPIRIT) [1] guidelines and checklist *(Trials structured Study Protocol template).* Note: the numbers in curly brackets in this protocol refer to SPIRIT checklist item numbers. The order of the items has been modified to group similar items (see http://www.equator-network.org/reporting-guidelines/spirit-2013-statement-defining-standard-protocol-items-for-clinical-trials/).

**Table:** 

Title {1}	Glucagon-like peptide-1 analogues: a new way to quit smoking? (SKIP)—a structured summary of a study protocol for a randomized controlled study
Trial registration {2a and 2b}.	Registered June 29, 2017, on ClinicalTrials.gov: NCT03204396
Protocol version {3}	12.3.2018, Version 4.0
Funding {4}	This study is investigator-initiated. Sources of funding for each author: BW: this study is supported by a grant by the Swiss National Foundation, the Gottfried Julia Bangerter-Rhyner Stiftung, the Goldschmidt-Jacobson Foundation, the Hemmi-Foundation and by funds of the University of Basel. SL: research is funded by the Department of Internal Medicine of the University Hospital of Basel, the Gottfried Julia Bangerter-Rhyner Stiftung and the Swiss Academy of Medical Sciences. BS: is supported by the Research Foundation of the University of Basel. The funders are not involved in designing, conducting nor analyzing the study or reporting study results. Other Support: The University Hospital Basel (and the integrated Clinical Trial Unit and Department of Endocrinology, Diabetology and Metabolism) provide the location and infrastructure.
Author details {5a}	1 Endocrinology, Diabetology and Metabolism, Department of Internal Medicine, University Hospital Basel, Petersgraben 4, 4031 Basel, Switzerland 2 Medical Outpatient Department, University Hospital Basel, Petersgraben 4, 4031 Basel, Switzerland 3 Department of Cardiology, University Hospital Basel, Basel, Switzerland 4 Faculty of Medicine, University of Basel, Basel, Switzerland 5 Division of Cognitive Neuroscience, Department of Psychology and Transfaculty Research Platform, University of Basel, Basel, Switzerland 6 Department of Clinical Research, University of Basel and University Hospital of Basel, Basel, Switzerland 7 Research Center for Clinical Neuroimmunology and Neuroscience Basel (RC2NB), University of Basel and University Hospital of Basel, Basel, Switzerland 8 Meta-Research Innovation Center at Stanford (METRICS), Stanford University, Stanford, CA, USA 9 Meta-Research Innovation Center Berlin (METRIC-B), Berlin Institute of Health, Berlin, Germany 10 Roche Innovation Centre Basel, F. Hoffmann- La Roche, Basel, Switzerland
Name and contact information for the trial sponsor {5b}	Principle Investigator: Bettina Winzeler Endocrinology, Diabetology and Metabolism, University Hospital Basel Petersgraben 4, 4031 Basel, Switzerland Email: bettina.winzeler@usb.ch Phone: + 41 265 25 25
Role of sponsor {5c}	The funders of the study have no role in study design, data collection, data analysis, data interpretation, or writing the report.

## Introduction

### Background and rationale {6a}

Smoking cessation is a health priority as smoking is the leading preventable cause of premature death [2]. Although most smokers wish to quit, 1-year quit rates after an unaided attempt are very low (3–6%) [3, 4]. If smokers participate in a smoking cessation programme making use of the most effective treatment—a combination of behavioural and pharmacotherapy—abstinence rates after 1 year are higher (approximately 30%), but still unsatisfactory according to two systematic reviews of 2013 and 2016 [5, 6].

Smokers encounter several difficulties when they try to quit. Nicotine is a potent psychoactive drug that causes physical dependence [7], and withdrawal symptoms put patients at risk for early relapse. Post-cessational weight gain is another substantial barrier [8]. On average, people who quit smoking show an increase in mean body weight of 4–5 kg within 12 months [9, 10]. The increased short-term risk of type 2 diabetes observed after smoking cessation [11–13] is directly proportional to weight gain [14]. Current pharmacotherapies for smoking cessation (e.g. nicotine replacement therapy, varenicline and bupropion) have not shown to reduce post-cessational weight gain [15, 16]. To maximize smoking cessation rates, novel strategies should address both nicotine withdrawal syndrome and unfavourable metabolic effects of smoking cessation (e.g. weight gain, diabetes).

The gut-brain hormone glucagon-like peptide-1 (GLP-1) is released from endocrine cells from the distal gut and centrally by neurons in the nucleus of the solitary tract of the hindbrain in response to food consumption [17–19]. GLP-1 receptors are expressed in several brain areas, including the hypothalamus and the reward nodes ventral tegmental area and nucleus accumbens [20–22], implicating a role of GLP-1 in reward regulation [23]. Due to its insulinotropic and satiation-promoting effects, GLP-1 analogues are widely used to treat type 2 diabetes and obesity. GLP-1 might not only play a role in regulating appetite and energy intake (likely via food-related reward processing), but also in controlling reward induced by addictive drugs such as alcohol, amphetamine or nicotine, as suggested by recent studies in animals and by one human study [24–26]. Preclinical studies suggested that nicotine activates GLP-1 neurons [27] while GLP-1 analogues may lead to modified nicotine-induced effects on the mesolimbic dopamine system [28], abolishing nicotine reward and decreasing nicotine intake [27]. In humans, a preliminary study investigating exenatide for smoking cessation in individuals with prediabetes or overweight found increased smoking abstinence rates (46.3% versus 26.8%) and a tendency of lower craving and post-cessation weight compared to placebo after 6 weeks [29].

Therefore, GLP-1 analogues may bring together all important properties for a promising novel therapy for smoking cessation: targeting withdrawal symptoms and preventing weight gain and diabetes. Hence, the aim of this study is to investigate the GLP-1 analogue dulaglutide as a potential therapy for smoking cessation.

## Objectives {7}

The primary objective is to assess the effect of dulaglutide combined with standard of care (SOC) consisting of behavioural counselling and pharmacotherapy with varenicline, on smoking quit rates after 12 weeks compared to placebo and SOC. Further objectives include assessing post-cessational weight gain, nicotine craving analysis, glucose homeostasis and long-term nicotine abstinence.

In two substudies, we aim to explore underlying mechanisms of dulaglutide influencing (1) nicotine reward (“substudy functional magnetic resonance imaging (fMRI)”) and (2) post-cessational energy metabolism (“substudy energy”).

## Trial design {8}

**Fig. 1:**
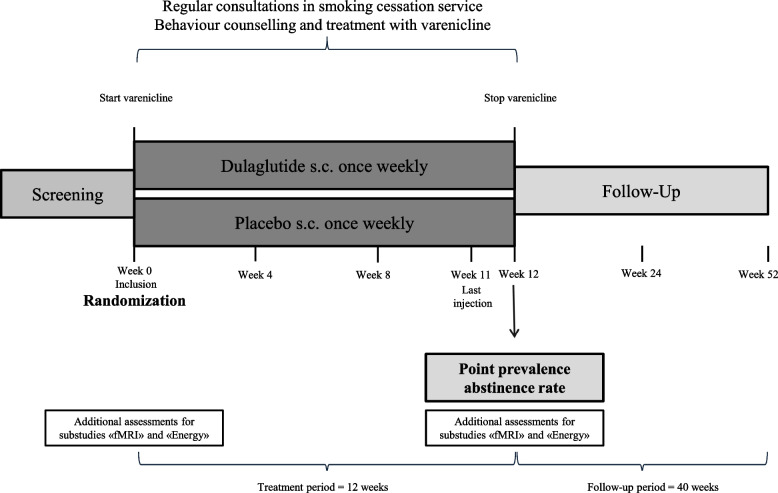
Study Flow

This is a placebo-controlled, double-blind, parallel group, superiority, single-centre randomized study including 255 patients. The study flow is shown in (Fig. 1).

## Methods: participants, interventions and outcomes

### Study setting {9}

Data are collected at the University Hospital Basel in Switzerland (single-centre study).

### Eligibility criteria {10}

Investigators proof the inclusion and exclusion criteria. Patients are eligible if they fulfil the following criteria and are willing to provide written informed consent.

#### Inclusion criteria

- Age 18 to 75 years
- Daily smokers who are willing to quit and exhibit one of the following criteria:
  -  ≥ 10 cigarettes per day or
  - At least moderate cigarette dependence defined by a Fagerstroem [30–32]
  - Score of ≥ 5 points or
  - Tobacco-associated disease
- Willing to get treatment with varenicline

For the “substudy fMRI”, only patients aged 18–60 years are eligible, as the risk of neurological changes increases with age. For the “substudy energy”, a BMI of 18–30 kg/m^2^ is required, as pronounced under- and overweight influences energy expenditure.

#### Exclusion criteria

- Pregnancy (incl. wish to become pregnant within next 3 months) or breast feeding
- Pre-existing treatment with GLP-1 agonists
- History of pancreatitis
- Severe renal insufficiency (estimated glomerular filtration rate < 30 ml/min/1.73 m.^2^)
- Instable psychiatric conditions
- Anorexia nervosa

For the “substudy fMRI”, additional exclusion criteria apply: medical conditions that affect brain function (e.g. stroke, epilepsy, space-occupying lesions, multiple sclerosis, Parkinson’s disease, dementia, transient ischemic attack), current use of medications that alter brain function, current illicit drug abuse including marijuana, alcohol (≤ 1 drink per day allowed; in Switzerland, 1 standard drink corresponds to 33 cl 5% beer, 13 cl wine or a drink or shot based on 4 cl 40% liquor [33]), claustrophobia, cardiac pacemaker, electronic device or ferromagnetic metal foreign bodies.

### Who will take informed consent? {26a}

Investigators obtain written informed consent from potential trial participants.

### Additional consent provisions for collection and use of participant data and biological specimens {26b}

Additional participant data and biological samples (plasma specimens) are obtained to be stored for use in future studies. Participants declare informed consent for this procedure.

## Interventions

### Explanation for the choice of comparators {6b}

To causally attribute the effect to dulaglutide (and not to the interventional setting) and prove that the agent modulates smoking cessation, we choose a placebo-controlled design for the study.

### Intervention description {11a}

The study medication (dulaglutide or placebo) is injected subcutaneously in the abdomen or thigh once weekly for 12 weeks at the study centre.

Dulaglutide is purchased commercially as Trulicity® [34] pen 0.75 mg and 1.5 mg. The first injection contains dulaglutide 0.75 mg/0.5 ml while for the second to the twelfth injection, a dose of 1.5 mg/0.5 ml is given. The dulaglutide doses used in this study correspond to recommended doses for the treatment of diabetes mellitus and show a favourable safety profile [35–38].

During the study period, all participants in both groups are followed at the smoking cessation service and receive standardized care. Treatment consists of a combination of individual advice, behavioural counselling and pharmacotherapy with varenicline according to current national guidelines for smoking cessation [39]. As varenicline is associated with gastrointestinal symptoms, all participants are instructed to ingest varenicline together with a meal to obtain optimal tolerability.

### Criteria for discontinuing or modifying allocated interventions {11b}

Predominant adverse effects on dulaglutide treatment are gastrointestinal symptoms with nausea (up to 20%) as the largest component. As gastrointestinal symptoms are dose-depended and peak during the first 2 weeks of treatment, the first injection is given at a dose of 0.75 mg. In case of persisting gastrointestinal side effects during the study period, the dosage can be kept at 0.75 mg or be reduced from 1.5 to 0.75 mg. In exceptional cases based on the investigators’ judgement, they may decide to skip an injection (e.g. pronounced local reactions or severe gastrointestinal side effects).

### Strategies to improve adherence to interventions {11c}

The study medication is injected on site. It is usually injected once weekly on a fixed day, but a time span of ± 3 days is allowed (= one injection/week, independent of the day). Participants not presenting to the planned study visits are contacted by phone and a new appointment is arranged. If participants are not able to present at the study centre, a study visit can take place as a home visit.

If the time span of a week cannot be adhered, the visit is skipped. For withdrawn participants, an end-of-study (EoS) visit is arranged in order to complete data collection and assess possible adverse events (AEs). If they agree, they are contacted at weeks 12, 24 and 52 and smoking status is assessed. Whenever possible, self-reported smoking abstinence is biochemically validated by CO measurement (e.g. at a home visit).

### Relevant concomitant care permitted or prohibited during the trial {11d}

Whenever possible, any additional treatment during the study period should be avoided. If concomitant medication is strongly recommended, the investigator decides whether the study is continued.

### Provisions for post-trial care {30}

In accordance with applicable regulations and Good Clinical Practice (GCP), a study monitor periodically controls study procedures.

In case participants suffer harm caused by the study, a corresponding insurance is in place that would cover the costs.

### Outcomes {12}

The primary outcome is the point prevalence abstinence rate at week 12 after randomization. It is assessed at week 12 (within a ± 7-day time window) and defined as self-reported 7-day nicotine abstinence *and* end-expiratory exhaled carbon monoxide (CO) measurement of 10 ppm or less.

The main secondary outcome is the change of body weight in kg (and BMI [kg/m^2^]) at week 12 relative to baseline.

Further secondary outcomes are:

- Point prevalence abstinence at weeks 24 and 52 (self-reported 7 days abstinence), biochemically validated by end-expiratory exhaled CO (10 ppm or less)
- Prolonged abstinence at weeks 24 and 52, defined by the condition that the participant was already previously abstinent (i.e. at week 12 for prolonged abstinence at week 24 and at weeks 12 and 24 for prolonged abstinence at week 52)
- Smoking reduction by number of cigarettes per day and by end-expiratory exhaled CO (reduction of more than 50%) at weeks 12, 24 and 52 compared to baseline
- Craving at weeks 4 and 12 relative to baseline assessed by the German version of the Questionnaire of Smoking Urge [40] (QSU-G; see Additional file 1) and a visual analogue scale (VAS; see Additional file 2)
- Change of body weight in kg (and BMI [kg/m^2^]) at weeks 4, 8, 24 and 52 relative to baseline
- Change in haemoglobin A1c (HbA1c) in per cent at weeks 12, 24 and 52 relative to baseline
- Switch from tobacco cigarettes to alternative smoking products (iQOS [heating of tobacco], electronic cigarettes [inhalation of vapour], cannabis, over-the-counter nicotine substitutes) or to medically prescribed nicotine substitutes
- Electronic cigarettes and IQOS at weeks 12, 24 and 52
- New-onset treatment with nicotine replacement therapy at weeks 12, 24 and 52

Outcomes of the substudy fMRI are as follows:

- Behavioural outcomes include craving measured by VAS and working memory performance investigated by the N-back task scores
- Functional neuronal changes are assessed through the surrogate of blood oxygenated level-dependent signal, an indirect measure of neural activity. Three echo-planar imaging sequences are performed to investigate neural substrates of nicotine craving, working memory and resting state functional networks
- Structural outcomes include changes of grey and white matter properties in regions parts of the reward pathway (i.e. anterior cingulate cortex, insula, striatum) and in subcortical regions
- Changes in cerebral blood flow in the reward pathway are also assessed using an Arterial Spin Labelling (ASL) sequence

All outcomes for this substudy are assessed at week 12.

We consider the following outcomes for substudy energy:

- Change of resting energy expenditure [29] (non-smokers versus persistent smokers; dulaglutide versus placebo treatment)
- Change of body composition (lean body mass and fat mass) as assessed by bioelectrical impedance analysis
- Haemodynamic parameters (cardiac index, peripheral vascular resistance, volemia, inotropy and vasoactivity) in dulaglutide versus placebo-treated participants and in non-smokers versus persistent smokers
- Change in sympathetic activity in dulaglutide versus placebo-treated participants and in non-smokers versus persistent smokers
- Change in eating habits and physical activity in dulaglutide versus placebo-treated participants and in non-smokers versus persistent smokers

All outcomes for this substudy are assessed at week 12; haemodynamic parameters are assessed at weeks 13 and 14 additionally.

### Participant timeline {13}

The participant timeline and study procedures are presented in Table 1.

**Table 1:** Timeline and study procedures

Study Procedures Week		0	1	2	3	4	5	6	7	8	9	10	11	12	24	52	
Study visit		V0	V1	V2	V3	V4	V5	V6	V7	V8	V9	V10	V11	V12	V13	V14	EoS
Eligibility assessment	X																
Inclusion/randomization		X															
Smoking status/CO measurement		X	X	X	X	X	X	X	X	X	X	X	X	X	X	X	X
Medical history		X															
Weight, BMI, vital signs		X				X				X				X	X	X	X
Questionnaire for smoking urge		X				X								X	X	X	X
Laboratory testing (blood and urine)		X												X	X	X	X
Study drug		X	X	X	X	X	X	X	X	X	X	X	X	X			
Adverse events			X	X	X	X	X	X	X	X	X	X	X	X	X	X	X
Substudies																	
- fMRI		X												X			X
- Indirect calorimetry		X												X			X
- Body impedance analysis		X												X			X
- HOTMAN®		X												X	X	X	X
- Serum catecholamines		X												X			X

*EoS* End of study visit, *V* Visit, *CO* Carbon monoxide

Before inclusion, information about the study is given and eligibility criteria are checked.

#### Baseline visit

After informed consent is obtained, participants are randomized. Baseline data and information regarding smoking are then assessed by questions and questionnaires. A short physical examination is performed, as well as a pregnancy test in premenopausal females. Smoking and nicotine exposure are assessed by a CO measurement (Micro + TM Smokerlyzer® [41] and a cotinine test in the spot urine (Urine Cotinine All Test COT 3 in 1 [42]). The participants then receive the first dose of study medication or placebo. The medication with varenicline is started on the same day.

#### Treatment phase

During the treatment phase of 12 weeks, weekly visits take place and tolerability of the study medication, potential AEs, self-reported smoking status and CO measurement as well as smoking urge by VAS are assessed; doses of the study medication and varenicline are documented. At visits 4, 8 and 12, short clinical assessments and, at visit 12, blood and urinary analyses for cotinine measurements additionally take place. At visit 12, motivation to stop smoking is assessed by a questionnaire in persistent smokers and consumption of alcohol or other substances (e.g. cannabis, opiate, benzodiazepine or illicit drugs) is documented for all participants.

#### Follow-up phase

At visit 13 (week 24) and visit 14 (week 52), potential AEs are assessed as well as self-reported smoking status, validated by CO measurement and smoking urge by VAS. A short clinical examination and blood and urinary analyses for cotinine measurements also take place.

#### Laboratory assessments

The blood samples drawn on visits 1, 12, 13 and 14 are immediately centrifuged and stored at − 70 °C (Biobank Department Endocrinology) for further analyses. HbA1c is measured immediately, but investigators and study staff are blinded to these results.

#### Substudy fMRI

In the stubstudy fMRI, participants undergo two fMRI sessions at baseline and between weeks 10 and 12 of the treatment phase. Before the fMRI session, the average of daily smoked cigarettes during the last 3 days and the timepoint of the last cigarette smoked is assessed and the CO concentration in the exhaled air is measured. Moreover, smoking urge is assessed by the VAS.

The fMRI block includes the following structural and functional sequences: T1-weighting and diffusor tensor imaging structural sequences, an arterial spin labelling sequence, a resting state sequence, a nicotine craving task [43] to investigate nicotine craving and an N-Back task to investigate working memory-related brain activations [44, 45].

#### Substudy energy

In participants of the substudy energy, parameters related to energy metabolism are assessed at baseline and after 12 weeks of treatment (a time frame of 3 weeks between visits 9 and 12 is accepted) including indirect calorimetry, a bioelectrical impedance analysis, non-invasive thoracic bioimpedance measurement and measurement of plasma catecholamine and neuropeptide Y (NPY) levels. Furthermore, self-perceived eating habits and physical activity are assessed.

REE is measured by indirect calorimetry after an overnight fast. Outcome measures are kcal per 24 h, assessed by the volume of oxygen uptake (VO2) and expelled volume of carbon dioxide (VCO2) in millilitres per minute and calculated by the Weir equation REE = [3.9 × (VO2) + 1.1 (VCO2)] × 1.44. Body composition is assessed by bioelectrical impedance analysis. Measures are lean body mass and fat mass in kilogrammes.

Haemodynamic parameters as blood pressure (mmHg), heart rate (beats per minute), cardiac index (l/min/m^2^), peripheral vascular resistance (Pa [s/m^3^]), volemia, inotropy and vasoactivity are non-invasively assessed using the HOTMAN® System (Hemo Sapiens Medical Inc., Sedona, AZ, USA). Haemodynamic parameters by non-invasive thoracic bioimpedance measurement are additionally assessed at weeks 24 and 52.

Plasma catecholamine (epinephrine and norepinephrine) and NPY levels were measured in picogrammes per millilitre. NPY is a 36 amino acid peptide well known to potentiate the action of catecholamine postsynaptically through the Y1 receptor and inhibit presynaptically the catecholamine secretion through the Y2 receptor [46].

### Sample size {14}

For the sample size estimation of the main study, we used a simulation approach (*R* = 999). We calculated the power to reject the null hypothesis of no difference between the point prevalence abstinence rates in the control and intervention groups for various sample sizes (100–500) and differences (10–30 percentage points). We used a *χ*^2^-test at a significance level of 5%. Assuming a point prevalence abstinence rate of 33% in the control group, and an increase by 18 percentage points to 51% in the intervention group, 255 participants should provide a power of at least 80% to reject the null hypothesis.

The substudies are considered exploratory. Accordingly, the sample sizes are based on feasibility criteria such as costs, availability of staff, infrastructure and readiness of patients to participate. We plan to include 60 participants from the main study in the substudies “Energy” and “fMRI” each. We expect that this sample size suffices to generate meaningful results, allowing the generation of new hypotheses.

### Recruitment {15}

Participants are recruited in the smoking cessation service at the Medical Outpatient Department of the University Hospital Basel and by advertisements.

## Assignment of interventions: allocation

### Sequence generation {16a}

Randomization is done according to a computer-generated randomization list (1:1 block randomization with randomly selected, varying block sizes), produced by the Clinical Trial Unit of the University Hospital Basel. No stratification is used.

### Concealment mechanism {16b}

The randomization list is implemented in the electronic data capture system (secuTrial®) and remains concealed until the evaluation of the study. Only unblinded study nurses who are responsible for the application of the study intervention but otherwise not involved in the study are able to see the assignment of the study intervention.

### Implementation {16c}

The allocation sequence is done by a computer-generated randomization list produced by the Clinical Trial Unit of the University Hospital Basel. Participants are assigned to a sequentially numbered study ID (in chronological order), which is activated by investigators after inclusion and corresponds to the predefined randomization list. Only unblinded study nurses are able to see the assigned study intervention (placebo versus dulaglutide) of the participants. Unblinded study nurses are needed for injection of the study drug.

## Assignment of interventions: blinding

### Who will be blinded {17a}

Participants, healthcare providers and data collectors are blinded to treatment allocation. As injection devices of dulaglutide and placebo are not identical, unblinded study staff administers the injections. The unblinded study staff is otherwise not involved in the study. During the study medication injection, participants wear a blindfold and are not able to see the injection side or the injection device. This process is conducted and documented according to the respective standard operating procedure of the Clinical Trial Unit Basel.

### Procedure for unblinding if needed {17b}

In emergency situations, e.g. AEs, and up to the decision of the investigator if medically important, the blind of the participant is broken and the allocation codes in the sealed data system are revealed.

## Data collection and management

### Plans for assessment and collection of outcomes {18a}

#### Baseline visit

After informed consent is obtained, assessments are done by:

- Self-created questions (e.g. demographics (age, sex, …), medical history questionnaire (inclusive tobacco-associated disease and cardiovascular risk factors), smoking habits and motivation and arguments to stop smoking (assessed by a questionnaire), self-reported 7-day abstinence)
- Validated questionnaires (6-item Fagerstroem test to assess cigarette dependence, 32-item QSU-G that evaluates intention and desire to smoke, visual analogue scale (VAS) to assess craving, where 0 means “no urge at all” and 10 means “maximal smoking urge”
- Clinical assessments (vital signs: weight in kg, height in cm, calculated BMI, blood pressure and heart rate)
- Smoking and nicotine exposure is assessed by an end-expiratory exhaled CO measurement (Micro + TM Smokerlyzer® [41]) and a cotinine test in the spot urine (Urine Cotinine All Test COT 3 in 1 [42])
- Blood withdrawal (e.g. glucose homeostasis assessed by measurement of HbA1c; further laboratory tests; collection of aliquots)
- A pregnancy test in premenopausal females to exclude a pregnancy

#### Treatment and follow-up phase

Mentioned analyses at baseline are repeated at determinated follow-up visits.

The point prevalence abstinence rate is assessed by a questionnaire (self-reported 7-day abstinence), and exhaled CO measurement weekly, for the stricter definition urinary cotinine [42], is measured additionally at week 12.

Tolerability of the drug and symptoms (abdominal pain, nausea, vomitus, diarrhoea, local irritation or pain, allergic reaction) as well as further adverse events are assessed on all study visits.

#### Processes to promote data quality

For accurate data collection, study nurses and study physicians are trained. The data transmission in the electronic database is checked for completeness. Completeness of data for primary and secondary outcomes at weeks 0, 12, 24 and 52 is checked by hand additionally. Regular study meetings take place. Additionally, monitoring visits take place.

### Plans to promote participant retention and complete follow-up {18b}

Weekly and personal visits, personal motivation to obtain study information and a small financial compensation after participation should have a positive impact on continuity. The importance of the 12 visits is explained to the participants, and we try to be flexible and to postpone visits in the time span of ± 3 days on the request of participants. In case of skipped visits, attempts are made to collect data otherwise (e.g. by telephone or e-mail). For withdrawn participants, an end-of-study visit is arranged in order to complete data collection (with the assessment of adverse events, medical history, smoking status, craving, reason of premature study discontinuation).

### Data management {19}

Study data are recorded in an encrypted fashion in case report forms (CRF) compliant to GCP and transferred to an electronic data capture system (secuTrial®).

Password protection ensures that only authorized persons can enter the system to view, add or edit data according to their permissions. Data can be validated for completeness and discrepancies automatically. An audit trail system maintains a record of initial entries and changes.

### Confidentiality {27}

All personal and medical information obtained for this study is confidential and disclosure to third parties is prohibited. Patients’ data are identified by study and subject ID number. Confidentiality of the patients is maintained by assigning patients a study number, keeping identifiers separate from the data and storing data in a locked file in the department of Endocrinology, Diabetes and Metabolism.

The study team (e.g. nurses, physicians or sponsor-investigator, respectively) has access to the source data (this is the paper form/hardcopy form) and to the electronical data. Intern statisticians have access to the anonymized data set.

Further parties, e.g. other researchers, may have access rights to the anonymized data set on demand after approval by the steering committee of this study.

### Plans for collection, laboratory evaluation and storage of biological specimens for genetic or molecular analysis in this trial/future use {33}

Biological material, i.e. blood samples, are handled according to GCP and good laboratory practice. Samples are collected in secure containers (Sarstedt Monovette®) and then centrifuged to collect plasma. Plasma is stored at − 80 °C in a thermo-controlled ultra-deep freezer.

## Statistical methods

### Statistical methods for primary and secondary outcomes {20a}

A detailed description of the reporting and statistical analysis of the data collected in this study (*statistical report and analysis plan*) is planned to be finalized before database closure. All analyses are performed in R version 4.1.1 or higher [47]. The analyses of the main study and the substudies are planned and performed in separate steps.

#### Analysis sets

The full analysis set consists of all patients who are randomized and patients are analysed according to their group of randomization in the intention-to-treat set (ITT-S). The per-protocol analysis set (PPS) consists of all patients in the ITT-S without any major protocol violations and who received at least 80% of study medication (10/12 doses), which is considered necessary to achieve a “response” to the medication and to observe the postulated effect of dulaglutide.

The analysis sets for the two substudies include all respective participants.

#### Primary analysis

The primary analysis assesses the difference in the point prevalence abstinence rates at week 12 between the verum and placebo arm. The difference is tested for a difference from zero using Pearson’s *χ*^2^-test. The difference in the point prevalence abstinence rates is reported with 95% confidence interval (CI; Wilson score method with continuity correction). The primary analysis is performed as an intention-to-treat analysis on the ITT-S.

Estimands and supplementary analyses to further examine the effect of dulaglutide on the primary outcome comprise (1) per-protocol analysis (primary analysis on PPS, see the “Analysis sets” section above); (2) analysis of all randomized participants while assuming that patients with missing data at week 12 are failed to quit smoking and are considered as smokers; (3) analysis of “strict abstinence” while negative urine cotinine measurement complements the definition of smoking abstinence (self-reported smoking abstinence and biochemically validated CO measurement ≤ 10 ppm). This analysis allows to identify smokers who stopped smoking just recently, or continue smoking a few days per week who might have sufficiently low CO measurements to pass as non-smokers as defined for the primary outcome; (4) logistic regression with total doses of dulaglutide as a single predictor; and (5) logistic regression with total doses of varenicline, nicotine substitute, baseline HbA1c and BMI as covariates.

#### Secondary analyses

In confirmatory analyses, we estimate the treatment effect of dulaglutide on secondary outcomes and test it for a difference from zero. Continuous outcomes are analysed using linear regression models with treatment (dulaglutide–placebo) as the categorical predictor and the baseline value as an adjusting covariate. The estimated, baseline-adjusted treatment effect is reported with 95% CI. For binary outcomes, the difference in proportions (dulaglutide–placebo) is provided with 95% CI and tested for a difference from zero using Pearson’s *χ*^2^-test. Descriptive analyses of all secondary outcomes include summary statistics for each time point according to treatment (mean and standard deviation or median and 1st and 3rd quartiles for continuous outcomes; frequencies and percentages for binary outcomes). All secondary analyses are performed as intention-to-treat analyses on the ITT-S, and confirmatory analyses also as per-protocol analyses on the PPS.

Since we aim to analyse short-term outcomes first, before long-term assessments are complete, we will adjust for short-term (up to week 12) and long-term (week 24 and later) outcomes separately. Confirmatory analyses of long-term outcomes will only be performed if a short-term treatment effect has been shown. We assume that outcomes related to smoking behaviour, weight and glucose metabolism measure separate treatment effects. Within each of the corresponding six groups of hypotheses, we will apply the Bonferroni-Holm procedure to keep the family-wise error rates at a level of 5%.

#### Safety analyses

Based on the ITT-S, the number and proportions of patients with adverse and serious AEs will be reported by the study arm. The most common events will be listed in more detail.

### Interim analyses {21b}

No interim analysis is planned.

### Methods for additional analyses (e.g. subgroup analyses) {20b}

We will use data collected during this study for further analyses investigating the following topics:

- Factors predicting successful smoking cessation and smoking relapse
- Sex differences in smoking cessation
- Effect of dulaglutide and smoking cessation on blood pressure and cardiovascular risk factors
- Effect of dulaglutide on alcohol and other reward-related behaviour
- Effect of dulaglutide and smoking cessation on inflammatory markers (e.g. pro-inflammatory cytokines)
- Effect of dulaglutide and smoking cessation on markers of platelet aggregation/function

### Methods in analysis to handle protocol non-adherence and any statistical methods to handle missing data {20c}

Multiple attempts will be made to obtain missing data. If participants are not able to present at the study centre, the CO measurement and urinary test (cotinine) can be obtained at a home visit. According to the standard procedure in most of the smoking cessation trials [48] and a proposal of a common standard for the outcome criteria in smoking cessation trials [49], we assume that missing data in outcomes related to abstinence are not missing at random but for reasons closely linked to the smoking status and we will consider such patients as smokers. For binary outcomes, we will hence impute the value corresponding to smokers. For continuous outcomes, missing values will be imputed by multiple imputation using chained equations in case of more than 5% missing values (per outcome). Otherwise, analyses will be performed on complete cases.

### Plans to give access to the full protocol, participant-level data and statistical code {31c}

We may share de-identified, individual participant-level data and related documents, including the study protocol and the statistical analysis plan. Data will be available with the publication of the main manuscript on receipt of a request detailing the study hypothesis and statistical analysis plan. The steering committee of this study will discuss all requests and decide on the basis of the scientific rigour of the proposal whether data sharing is appropriate. All applicants are asked to sign a data access agreement.

## Oversight and monitoring

### Composition of the coordinating centre and trial steering committee {5d}

The sponsor-investigator is responsible for the planning, implementation, completion and analysis of the study results. Study physicians do the recruitment, screening, inclusion, weekly visitation and follow-up of (potential) participants and are partially involved in the analysis. Also, study nurses conduct weekly and follow-up visits. Organizational steps are carried out by the sponsor, study physicians or nurses according to a separate agreement. Statistical analysis is done by statisticians and study physicians. Weekly meetings with the study team are held, and, if necessary, urgent inquiries with the sponsor-investigator are possible. The data management team is responsible for the set-up, adjustments and closure of the electronic data capture system.

### Composition of the data monitoring committee, its role and reporting structure {21a}

The monitoring of the study is performed by the Clinical Trial Unit of the University Hospital Basel. It is independent from the sponsor and has no competing interests.

### Adverse event reporting and harm {22}

At each visit, participants’ well-being is assessed and reported symptoms are documented. The relatedness to the study medication is judged (causality assessment of the event to the study medication [definitively, probably, possibly, unlikely, not related or not assessable]) based on the criteria listed in the ICH E2A guidelines [50]. If events are classified as serious [being defined as any untoward medical occurrence that results in death, is life-threatening, requires in-patient hospitalization or prolongation of existing hospitalization, results in persistent or significant disability/incapacity, or is a congenital anomaly/birth defect], they are reported to the respective authorities according to law.

### Frequency and plans for auditing trial conduct {23}

Authorized representatives of the national or local authorities will be permitted to inspect or audit the facilities and records relevant to this study.

### Plans for communicating important protocol amendments to relevant parties (e.g. trial participants, ethical committees) {25}

The sponsor-investigator and the principal investigators are allowed to amend the protocol or to provide suggestions for a protocol amendment. Substantial amendments are only implemented after approval of the Competent Ethics Committee and if required of the Swissmedic, respectively. All non-substantial amendments are communicated as soon as possible or within the Annual Safety Report, respectively.

## Dissemination plans {31a}

The results of this study shall be published in a peer-reviewed journal and presented at scientific conferences. No use of professional writers is intended.

## Discussion

Cigarette smoking has a tremendous impact on morbidity and mortality and puts a significant burden on the public health system. As shown in a recent study, excessive cardiovascular risk can be abolished by quitting smoking [14]. However, smoking quit rates are unsatisfactory even if smokers participate in a dedicated programme and make use of available pharmacological treatment [5, 6].

GLP-1 analogues are widely studied and used as food-intake regulating hormones. It is likely that food-related and drug-induced reward processing share common mechanisms. Based on recent animal data, showing that GLP-1 controls reward induced by nicotine and other addictive drugs, similar effects are assumed in humans [24, 51, 52] and several studies are ongoing (NCT03232112 [53]; NCT02690987). To date, one clinical study investigating GLP-1 RA as a smoking cessation therapy has been published. The pilot randomized controlled study by Yammine et al. [29] included 84 smokers who were randomized to the GLP-1 RA exenatide or placebo in addition to nicotine replacement therapy. After a 6-week treatment period, smoking abstinence was improved while craving, withdrawal symptoms and weight were reduced on exenatide compared to placebo. The results of this study are very reassuring, but the intervention period was short and only overweight or prediabetic smokers were included. In comparison, our study evaluates GLP-1 analogues in a broader cohort of both normal weight and overweight smokers with and without diabetes and assesses quit rates at a later—and clinically more relevant—timepoint. We use the nicotinic receptor partial agonist varenicline [54] plus behaviour counselling as standard treatment as varenicline has been shown to be the most efficient single pharmacological treatment [6], leading to an abstinence rate of 33.5% after 12 weeks [55]. This approach is in line with national guidelines [39], which recommend a pharmacological treatment with varenicline, nicotine replacement therapy or bupropion in smokers with moderate to severe nicotine dependency. Although some randomized study evidence suggests further benefit by adding nicotine replacement therapy to varenicline [55, 56], this combination had not been adopted in national guidelines in Switzerland at the time of the study start. Varenicline reduces symptoms of nicotine withdrawal as a selective nicotinic receptor partial agonist. Previous concerns about neuropsychiatric issues of varenicline have not been confirmed by a large randomized controlled study [55]. To date, no data are available about the possible interaction between varenicline and GLP-1 analogues. Both drugs are assumed to influence reward regulation. By binding to different receptors, the combination of the drugs may potentiate their anti-addictive effect and lead to the desired nicotine aversion.

Besides influencing craving and nicotine withdrawal symptoms, GLP-1 analogues may prevent unfavourable metabolic effects of smoking cessation especially weight gain, which is the main reason for not quitting and/or relapsing, and diabetes. Post-cessational weight gain seems to occur during the first months and year [10], being as high as 2.3 kg after 2 months [15]. A temporary increase in the risk of type 2 diabetes is only observed in quitter with post-cessational weight gain [14]. Therefore, post-cessational weight control has to be incorporated in smoking cessation strategies. Given the weight and glucose-lowering properties of GLP-1 analogues, we expect a reduced proportion of new-onset prediabetes or diabetes in dulaglutide versus placebo-treated participants.

### Limitations

This study focuses on the mechanistic properties of GLP-1 RA in this new explanatory design [57]. The treatment phase was therefore limited to 12 weeks and the primary outcome is assessed at the end of these 12 weeks, when the most significant effects are expected. However, the clinically most relevant question is long-term abstinence and a longer treatment period might be necessary. In case of a beneficial effect on short-term smoking cessation rates, a larger multicentre study addressing long-term abstinence under GLP-1 analogues will be designed. The abrupt treatment stop after 3 months may put participants at risk for weight rebound or even smoking relapse. Therefore, a more individualized approach (prolonged treatment, dosage tapering) may be needed.

### Conclusion

This study investigates the GLP-1 analogue dulaglutide as a novel therapy for smoking cessation with the hypothesis that dulaglutide may target withdrawal symptoms on the one hand and preventing weight gain on the other. This study has the potential to unlock a new therapy for smokers who wish to overcome their addiction and is, therefore, of high relevance and broad impact.

## Trial status

The protocol is version 4.0, March 12, 2018. The trial commencement was in June 2017. The anticipated completion date (last patient out) is July 2022. The protocols of the substudies fMRI are version 1.0 dated October 13, 2017.

## Supplementary Information

**Additional file 1.** QSU-G.

**Additional file 2.** VAS smoking urge.

**Additional file 3.** Clinical study protocol (main study).

**Additional file 4.** Clinical study protocol (SKIP fMRI).

**Additional file 5.** Clinical study protocol (SKIP Energy).

**Additional file 6.** Informed consent form (energy), informed consent form (fMRI), informed consent form (main study).

## Data Availability

For detailed information, protocols of the main and substudies are available (Additional files 3, 4 and 5) in the Supplementary Materials. The sponsor and principal investigator as well as designated authors have access to the final study dataset. Any data required to support the protocol can be supplied on request after approval by the steering committee of this study.

## Abbreviations

AE: Adverse event
BMI: Body mass index
CI: Confidence interval
CO: Carbon monoxide
CRF: Case report forms
CTU: Clinical Trial Unit
EoS: End of study
ITT-S: Intention-to-treat set
fMRI: Functional magnetic resonance imaging
GLP-1: Glucagon-like peptide-1
GCP: Good clinical practice
HbA1c: Haemoglobin A1c
NPY: Neuropeptide Y
PPS: Per-protocol analysis set
QSU-G: Questionnaire on Smoking Urges
REE: Resting energy expenditure
SCS: Standardized smoking cessation service
SOC: Standard of care
VAS: Visual analogue scale
V: Visit
VO2: Volume of oxygen uptake
VCO2: Volume of carbon dioxide
