# Supplementary material for: Glucagon-like peptide-1 analogues: a new way to quit smoking? (SKIP)—a structured summary of a study protocol for a randomized controlled study
Source: Trials. 2023 Apr 20;24:284. doi: 10.1186/s13063-023-07164-9 (PMC10120253; doi:10.1186/s13063-023-07164-9)
Supplement: Supplementary file 1 — Additional file 1. QSU-G. [file 13063_2023_7164_MOESM1_ESM.pdf]

Julius-Maximilians-Universität Würzburg
Lehrstuhl für Psychologie I
Prof. Dr. P. Pauli

	**Subject Number: I__I__I__I**
Date I__I__I : I__I__I__I__I : I__I__I	□**V0** □**V4** □**V12**

The Skip Study

QSU-G- Fragebogen

Bitte lesen Sie die folgenden Aussagen aufmerksam durch. Kennzeichnen Sie die für Sie zutreffende Einschätzung mit einem Kreis. Bitte achten Sie auf Verneinungen durch das Wort "nicht".
Wir interessieren uns für Ihr momentanes Gefühl, also dafür, wie Sie sich genau jetzt, beim ausfüllen des Fragebogens fühlen.
Bitte beantworten Sie sorgfältig jede Frage.

		Stimmt überhaupt nicht — Stimmt völlig
1	Rauchen würde mir jetzt dazu verhelfen, mich sehr gut zu fühlen..	1 – 2 – 3 – 4 – 5 – 6 - 7
2	Ich wäre weniger reizbar, wenn ich jetzt rauchen könnte……..	1 – 2 – 3 – 4 – 5 – 6 - 7
3	Nichts wäre besser, als jetzt eine Zigarette zu rauchen………..	1 – 2 – 3 – 4 – 5 – 6 - 7
4	Mir fehlt das Rauchen jetzt gerade nicht………………………	1 – 2 – 3 – 4 – 5 – 6 - 7
5	Ich werde rauchen, sobald ich wieder die Möglichkeit dazu bekomme……………………………………………………	1 – 2 – 3 – 4 – 5 – 6 - 7
6	Ich möchte jetzt nicht rauchen……………………………...	1 – 2 – 3 – 4 – 5 – 6 - 7
7	Rauchen würde meine schlechte Stimmung deutlich verbessern…………………………………………………	1 – 2 – 3 – 4 – 5 – 6 - 7
8	Rauchen würde mir jetzt nicht helfen, mich zu beruhigen…......	1 – 2 – 3 – 4 – 5 – 6 - 7
9	Wenn ich jetzt eine Zigarette angeboten bekäme, würde ich sie sofort rauchen……………………………………………………..	1 – 2 – 3 – 4 – 5 – 6 - 7
10	Ich könnte ab sofort für eine lange Zeit auf das Rauchen verzichten………………………………………………………	1 – 2 – 3 – 4 – 5 – 6 - 7
11	Jetzt eine Zigarette zu rauchen, wäre nicht angenehm………..	1 – 2 – 3 – 4 – 5 – 6 - 7
12	Wenn ich jetzt im Moment rauchen würde, wäre ich weniger gelangweilt……………………………………………………..	1 – 2 – 3 – 4 – 5 – 6 - 7
13	Das Einzige, was ich jetzt gerne hätte, wäre eine Zigarette…....	1 – 2 – 3 – 4 – 5 – 6 - 7
14	Wenn ich jetzt eine Zigarette rauchen würde, würde ich mich weniger müde fühlen……………………………………………...	1 – 2 – 3 – 4 – 5 – 6 - 7
15	Jetzt zu rauchen, würde mich glücklicher machen……………..	1 – 2 – 3 – 4 – 5 – 6 - 7

16	Sogar, wenn es jetzt möglich wäre, würde ich wahrscheinlich nicht rauchen………………………………………………...	1 – 2 – 3 – 4 – 5 – 6 - 7
17	Ich habe jetzt gerade keinen Wunsch nach einer Zigarette…….	1 – 2 – 3 – 4 – 5 – 6 - 7
18	Mein Wunsch nach einer Zigarette scheint gerade so stark zu sein, dass er mich beinahe voll im Griff hat……………………	1 – 2 – 3 – 4 – 5 – 6 - 7
19	Wenn ich jetzt rauchen würde, wäre fast alles in Ordnung…….	1 – 2 – 3 – 4 – 5 – 6 - 7
20	Ich sehne mich gerade nach einer Zigarette………………….....	1 – 2 – 3 – 4 – 5 – 6 - 7
21	Ich würde eine Zigarette jetzt nicht genießen………………….	1 – 2 – 3 – 4 – 5 – 6 - 7
22	Eine Zigarette würde jetzt nicht gut schmecken…………….….	1 – 2 – 3 – 4 – 5 – 6 - 7
23	Ich verspüre jetzt gerade den Drang nach einer Zigarette……..	1 – 2 – 3 – 4 – 5 – 6 - 7
24	Ich hätte die Dinge gerade besser im Griff, wenn ich rauchen würde………………………………………………………..	1 – 2 – 3 – 4 – 5 – 6 - 7
25	Ich werde rauchen, sobald ich die Möglichkeit dazu habe…….	1 – 2 – 3 – 4 – 5 – 6 - 7
26	Ich würde mich körperlich nicht besser fühlen, wenn ich jetzt rauchen würde………………………………………………	1 – 2 – 3 – 4 – 5 – 6 - 7
27	Eine Zigarette wäre jetzt nicht sehr befriedigend……………....	1 – 2 – 3 – 4 – 5 – 6 - 7
28	Wenn ich jetzt eine angezündete Zigarette in der Hand hätte, würde ich sie wahrscheinlich gar nicht rauchen………………..	1 – 2 – 3 – 4 – 5 – 6 - 7
29	Wenn ich jetzt rauchen würde, könnte ich klarer denken……...	1 – 2 – 3 – 4 – 5 – 6 - 7
30	Ich würde fast alles tun, um jetzt eine Zigarette zu bekommen..	1 – 2 – 3 – 4 – 5 – 6 - 7
31	Ich muss jetzt rauchen……………………………………….	1 – 2 – 3 – 4 – 5 – 6 - 7
32	Gerade jetzt habe ich nicht vor, zu rauchen…………………...	1 – 2 – 3 – 4 – 5 – 6 - 7
