# Supplementary figures and images for: Glucagon-like peptide-1 analogues: a new way to quit smoking? (SKIP)—a structured summary of a study protocol for a randomized controlled study
Source: Trials. 2023 Apr 20;24:284. doi: 10.1186/s13063-023-07164-9 (PMC10120253; doi:10.1186/s13063-023-07164-9)

**Visual Analogue Scale smoking urge 0-10**

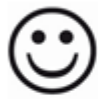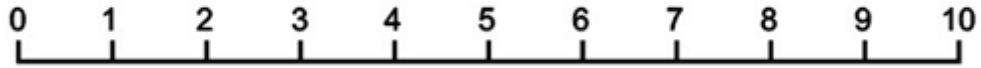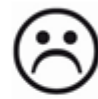

No urge at  
all for  
nicotine

Maximal urge  
for nicotine

Supplement: Supplementary file 2 — Additional file 2. VAS smoking urge. [file 13063_2023_7164_MOESM2_ESM.pdf]
