# Supplementary material for: Glucagon-like peptide-1 analogues: a new way to quit smoking? (SKIP)—a structured summary of a study protocol for a randomized controlled study
Source: Trials. 2023 Apr 20;24:284. doi: 10.1186/s13063-023-07164-9 (PMC10120253; doi:10.1186/s13063-023-07164-9)
Supplement: Supplementary file 3 — Additional file 3. Clinical study protocol (main study). [file 13063_2023_7164_MOESM3_ESM.pdf]

Clinical Study Protocol

Smoking cessation facilitated by glucagon-like peptide-1 (GLP-1) analogues – a randomized, double-blind, placebo-controlled trial

Study Type:	Clinical trial with Investigational Medicinal Product (IMP)
Study Categorisation:	Risk category B
Study Registration:	clinicaltrials.gov and kofam.ch intended
Sponsor-Investigator:	Prof. Mirjam Christ-Crain<br>Universitätsspital Basel, Petersgraben 4, 4031 Basel
Principal Investigator:	Dr. med. Bettina Winzeler
Co-Investigators:	Dr. med. Thilo Burkard<br>Dr. med. Andrea Meienberg<br>Dr. med. Nica Jeanloz<br>Dr. med. Fabian Meienberg
Investigational Product:	Dulaglutide (Trulicity®)
Protocol Version and Date:	Version 4, 12.03.2018<br>Version 3, 13.10.2017<br>Version 2, 03.05.2017<br>Version 1, 16.02.2017

Signature Page

Study number - BASEC 2017-00286

Study Title The SKIP-Study

The Sponsor-Investigator and trial statistician have approved the protocol version [4, 12.04.2018], and confirm hereby to conduct the study according to the protocol, current version of the World Medical Association Declaration of Helsinki, ICH-GCP guidelines and the local legally applicable requirements.

Sponsor Investigator: Prof. M. Christ-Crain

Basel, 12.3.18

Place/Date Signature

Principal Investigator: Dr. med. B. Winzeler

Basel 12.3.18

Place/Date Signature

Statistician: Dr. T. Erlanger

Basel, 12.3.18

Place/Date Signature

TABLE OF CONTENT

STUDY SYNOPSIS 7
STUDY SUMMARY IN LOCAL LANGUAGE 11
ABBREVATIONS 11
STUDY SCHEDULE 12
1. STUDY ADMINISTRATIVE STRUCTURE 13
1.1 Sponsor, Sponsor-Investigator 13
1.2 Principal Investigator 13
1.3 Statistician ("Biostatistician") 13
1.4 Clinical Epidemiologists 13
1.5 Laboratory 13
1.6 Monitoring institution 13
1.7 Data Safety Monitoring Committee 13
1.8 Authors' contributions 13
2. ETHICAL AND REGULATORY ASPECTS 14
2.1 Study registration 14
2.2 Categorisation of study 14
2.3 Competent Ethics Committee (CEC) 14
2.4 Competent Authorities (CA) 14
2.5 Ethical Conduct of the Study 14
2.6 Declaration of interest 14
2.7 Patient Information and Informed Consent 14
2.8 Participant privacy and confidentiality 15
2.9 Early termination of the study 15
2.10 Protocol amendments 15
3. BACKGROUND AND RATIONALE 15
3.1 Background and Rationale 15
3.2 Investigational Product (treatment, device) and Indication 16
3.3 Preclinical Evidence 16
3.4 Clinical Evidence to Date 16
3.5 Dose Rationale / Medical device: Rationale for the intended purpose in study (pre-market MD) 17
3.6 Explanation for choice of comparator (or placebo) 17
3.7 Risks / Benefits 17
3.8 Justification of choice of study population 17
4. STUDY OBJECTIVES 18
4.1 Overall Objective 18
4.2 Primary Objective and Hypothesis 18
4.3.1 Main Secondary Objective 18

4.3.2 Further Secondary Objectives 18
4.4 Safety Objectives 19
5. STUDY OUTCOMES 19
5.1 Primary Outcome 19
5.2 Secondary Outcomes 19
5.3 Safety Outcomes 19
6. STUDY DESIGN 19
6.1 General study design and justification of design 19
6.2 Methods of minimising bias 20
6.2.1 Randomisation 20
6.2.2 Blinding procedures 20
6.2.3 Unblinding Procedures (Code break) 20
7. STUDY POPULATION 20
7.1 Eligibility criteria 20
7.2 Recruitment and screening 21
7.3 Assignment to study groups 21
7.4 Criteria for withdrawal / discontinuation of participants 21
8. STUDY INTERVENTION 21
8.1 Identity of Investigational Products (treatment / medical device) 21
8.1.1 Experimental Intervention (treatment / medical device) 21
8.1.2 Control Intervention (standard/routine/comparator treatment / medical device) 22
8.1.3 Packaging, Labelling and Supply (re-supply) 22
8.1.4 Storage Conditions 22
8.2 Administration of experimental and control interventions 22
8.2.1 Experimental Intervention 22
8.2.2 Control Intervention 22
8.3 Dose / Device modifications 22
8.3.1 Dose reduction of the study drug 22
8.3.2 Dose reduction of varenicline 22
8.4 Compliance with study intervention 22
8.5 Data Collection and Follow-up for withdrawn participants 23
8.6 Trial specific preventive measures 23
8.7 Concomitant Interventions (treatments) 23
8.8 Study Drug / Medical Device Accountability 23
8.9 Return or Destruction of Study Drug / Medical Device 23
9. STUDY ASSESSMENTS 23
9.1 Assessment of primary outcome 23
9.2 Assessment of main secondary outcome 23
9.2.1 Assessment of further secondary outcomes: 23

9.2.2 Abstinence rate during follow-up phase 23
9.2.3 Smoking reduction 24
9.2.4 Craving 24
9.2.5 Body weight 24
9.2.6 Glucose homeostasis 24
9.2.7 Further explorative outcome measures 24
9.2.8 Assessment of safety outcomes 24
9.2.9 Assessments in participants who prematurely stop the study 24
9.3 Procedures at each visit 24
9.3.1 Screening phase: 24
9.3.2 Visit 0, Inclusion 24
9.3.3 Treatment phase, visits 2, 3, 4, 5, 6, 7, 8, 9, 10, 11, 12 25
9.3.4 Follow-up phase, visit 24 (week 24) and Visit 52 (week 52) 25
9.3.5 End of study visit for withdrawn participant 25
9.3.6 Visits within the SCS 25
10. SAFETY 25
10.1 Definition and assessment of (serious) adverse events and other safety related events 26
10.2 Reporting of serious adverse events (SAE) and other safety related events 26
10.3 Follow up of (Serious) Adverse Events 27
11. STATISTICAL METHODS 27
11.1 Hypothesis 27
11.2 Determination of Sample Size 27
11.3 Planned Analyses 28
11.3.1 Datasets to be analysed 28
11.3.2 Primary Analysis 28
11.3.3 Secondary Analyses 28
11.4 Interim Analysis 28
11.5 Safety Analysis 28
11.6 Deviation(s) from the original statistical plan 28
11.7 Handling of missing data and drop-outs 28
12. QUALITY ASSURANCE AND CONTROL 29
12.1 Data handling and record keeping / archiving 29
12.1.1 Case Report Forms 29
12.1.2 Specification of source documents 29
12.1.3 Record keeping / archiving 29
12.2 Data management 29
12.2.1 Data Management System 29
12.2.2 Data security, access and back-up 29
12.2.3 Analysis and archiving 29
12.2.4 Electronic and central Data validation 30

12.3 Monitoring 30
12.4 Audits and Inspections 30
12.5 Confidentiality, Data Protection 30
12.6 Storage of biological material and related health data 30
13. PUBLICATION AND DISSEMINATION POLICY 30
14. FUNDING AND SUPPORT 30
14.1 Funding 30
14.2 Other Support 31
15. INSURANCE 31
16. SIGNIFICANCE OF THE STUDY 31
17. REFERENCES 31
APPENDICES 35

STUDY SYNOPSIS

**Sponsor / Sponsor-Investigator**	Prof. Mirjam Christ-Crain
**Study Title:**	**Smoking cessation facilitated by glucagon-like peptide-1 (GLP-1) analogues – a randomized, double-blind, placebo-controlled trial**
**Short Title / Study ID:**	Smoking cessation by GLP-1 analogues
**Protocol Version and Date:**	Version 4, 12.03.2018
**Trial registration:**	clinicaltrials.gov and kofam.ch intended
**Study category and Rationale**	Category B: Dulaglutide is approved for treatment of diabetes mellitus, but not studied as pharmacotherapy for smoking cessation.
**Clinical Phase:**	Phase 2
**Background and Rationale:**	Cigarette smoking is the leading preventable cause of premature death worldwide. However smoking is a very difficult addiction to break and despite established smoking cessation programs quit rates are low, especially in the real-life setting. The main reasons for not quitting or relapsing after cessation are the nicotine withdrawal syndrome and post-cessational weight gain. GLP-1 analogues are well known to stimulate insulin secretion and to reduce energy intake and therefore body weight. Recent findings from animal and human studies suggest a role of GLP-1 in the pathophysiology of addiction. The putative role of GLP-1 analogues in nicotine reward regulation combined with its weight reducing effects might be of major interest in view of novel pharmatherapeutic options for smoking cessation.
**Objective(s):**	1. To evaluate the influence of the GLP-1 analogue dulaglutide combined with varenicline and behavioural counselling (=standard of care [SOC]) on point prevalence abstinence rate at 12 weeks compared to SOC alone.<br>2. To assess the influence of the GLP-1 analogue dulaglutide on post-cessation weight change compared to SOC.

**Outcome(s):**	The primary outcome is point prevalence abstinence rate at week 12 of dulaglutide treatment and SOC versus SOC alone, confirmed with end-expiratory exhaled carbon monoxide measurements of 10 ppm or less.<br>The main secondary outcome is change in body weight in kg (and BMI [kg/m²]) relative to baseline at week 12 of dulaglutide treatment versus placebo.<br>Further secondary outcomes are<br>point prevalence abstinence rate at week 24 and 52;<br>prolonged abstinence rate at week 24 and 52;<br>Smoking reduction at week 12, 24, and 52;<br>Change of craving at week 4 and 12 relative to baseline;<br>Change of body weight in kg (and BMI [kg/m²]) at week 4, 8, 24, and 52;<br>Change in haemoglobin A1c levels at week 12, 24, 52 relative to baseline

Study design:

Double-blind, placebo controlled, parallel group, superiority, single-center randomized trial

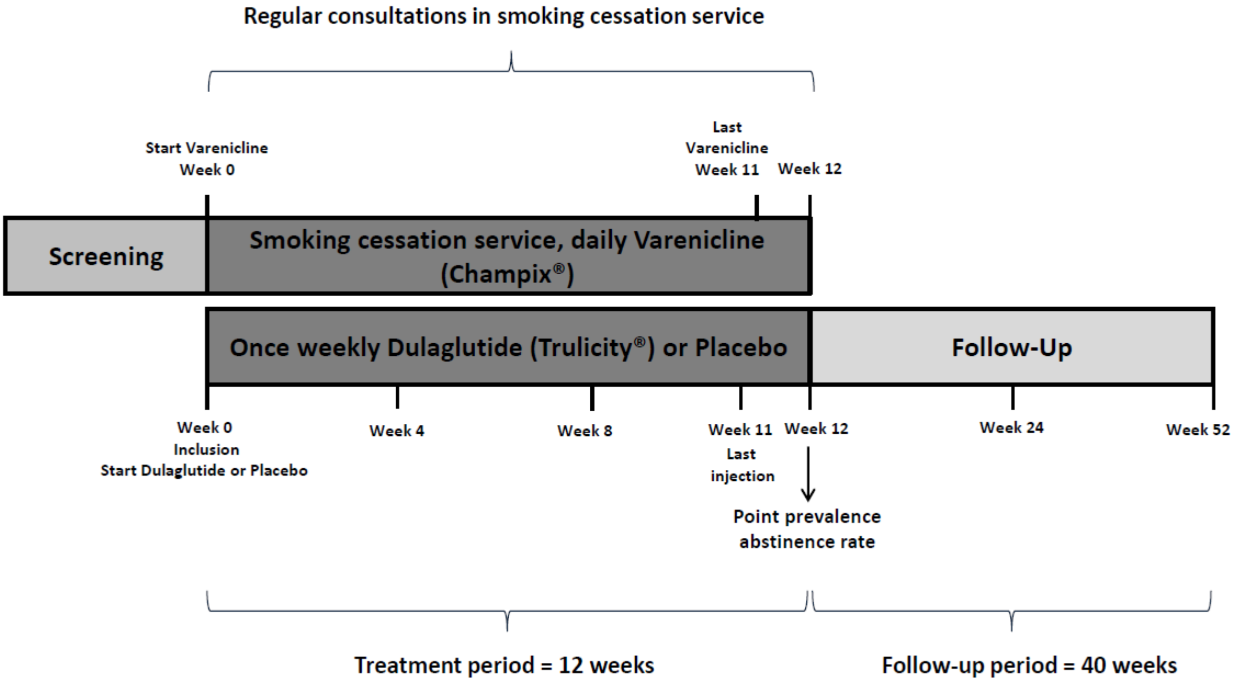

**Inclusion / Exclusion criteria:**	**Inclusion criteria:**<br>- Age 18 to 75 years<br>- Daily smokers who are willing to quit <u>and</u> exhibit one of the following criteria:<br>• ≥10 cigarettes per day **<u>or</u>**<br>• At least moderate nicotine dependence defined by a Fagerstroem Score of ≥5 points **<u>or</u>**<br>• Tobacco associated disease<br>- Treatment with varenicline (Champix®)<br>**Exclusion criteria:**<br>- Pregnancy (incl. wish to become pregnant within next 3 months) or breast feeding<br>- Pre-existing treatment with GLP-1 agonists<br>- History of pancreatitis<br>- Severe renal insufficiency (estimated glomerular filtration rate <30 ml/min/1,73 m^2)<br>- Instable psychiatric conditions<br>Anorexia nervosa
**Measurements and procedures:**	12-week treatment phase with dulaglutide (Trulicity®) or placebo and SOC, followed by a 40-week non-treatment phase. Assessment of smoking status by end-expiratory exhaled carbon monoxide measurements.
**Study Product / Intervention:**	Dulaglutide (Trulicity®) 0.75 mg and 1.5 mg in 0.5 ml, via pen s.c. once weekly for 12 weeks.
**Control Intervention (if applicable):**	Placebo: 0.5 ml normal saline (0.9% sodium chloride [0.9% NaCl]), injection s.c. via syringe once weekly for 12 weeks.
**Number of Participants with Rationale:**	Power calculation was done for the primary outcome. For calculating sample size a simulation was carried out . It was assumed that point prevalence abstinence rate in the control arm will be 33% and the increase of quit rate in the intervention arm 18% (increase from 33% to 51%) at week 12<br>For this trial a total of 256 patients should be recruited. Hypothesis testing to estimate efficacy of standard therapy plus dulaglutide is on the basis of 80% power and an *α* error threshold of 5%.
**Study Duration:**	Approximately 36 months
**Study Schedule:**	First-Participant-In (planned): 01.04.2017<br>Last-Participant-Out (planned): 31.03.2020
**Investigator(s):**	Prof. Mirjam Christ-Crain<br>Dr. med. Bettina Felicitas Winzeler<br>Universitätsspital Basel, Petersgraben 4, 4031 Basel
**Study Centre(s):**	Single-centre: Universitätsspital Basel

**Statistical Considerations:**	Primary analysis will assess whether point prevalence abstinence rate in the intervention arm after 12 weeks is significantly different to the control arm. A Pearson's χ^2 -test will be applied to test the hypothesis (H-Null: no difference H-Alternative: significant difference).<br><br>Main secondary analysis will assess whether change in body weight in kg (and BMI [kg/m²]) relative to baseline is significantly different between the experimental and the control arm at week 12. Difference between the 2 arms will be assessed by ANCOVA. Weight at week 12 will be the outcome variable, weight at baseline and treatment arm both explanatory variables.<br><br>Further secondary outcomes will be analysed as follows:<br>- For point prevalence abstinence rate at 24 and 52 weeks the same methods as for the primary analysis will be applied.<br>- For prolonged abstinence at 24 and 52 weeks, the same statistical methods as for the primary analysis will be applied except that only patients are included who were already abstinent at previous assessments (at week 12 for prolonged abstinence rate at 24 weeks and at weeks 12 and 24 for abstinence at week 52).<br>- For smoking reduction, it will be tested whether the difference in percentage of patients who have a reduced of end-expiratory exhaled CO of more than 50% at 12, 24, and 52 weeks is significantly different between the two arms. The Pearson's χ^2 -test will be applied.<br>- Whether craving between the two arms at week 4 and 12 relative to baseline is significantly different will be analysed by ANCOVA. Craving at week 4 or 12 will be the outcome variable, craving at baseline and treatment arm both explanatory variables.<br>- Change of body weight in kg (and BMI [kg/m²]) at week 4, 8, 24 and 52 will be assessed by ANCOVA. Weight at week 24 or 52 will be the outcome variable, weight at baseline and treatment arm both explanatory variables.<br>- Change of haemoglobin A1c (HbA1c) concentration at week 12, 24, and 52, relative to baseline will be analysed by ANCOVA. HbA1c at 12, 24, or 52 weeks will be the outcome variable, HbA1c at baseline and treatment arm both explanatory variables.<br><br>- Time trends of means or percentages of all secondary endpoints will be plotted including standard deviations for means and 95%-CI for percentages.<br><br>- For hypothesis testing an alpha error threshold of 5% will be chosen.
**GCP Statement:**	This study will be conducted in compliance with the protocol, the current version of the Declaration of Helsinki, as well as the GCP and all national legal and regulatory requirements.

STUDY SUMMARY IN LOCAL LANGUAGE

Nikotinkonsum ist mit einer deutlich erhöhten Morbidität und Mortalität vergesellschaftet. Obwohl viele Raucher gerne mit dieser Sucht brechen möchten, scheitern sie im Alltag an den Hindernissen, welche ein Rauchstopp mit sich bringt. Hier sind in erster Linie die Nikotin-Entzugserscheinungen und die Gewichtszunahme zu nennen. Ziel dieser Studie ist es, eine neue Behandlung zur Rauchentwöhnung zu untersuchen, welche genau auf diese zwei Barrieren abzielt.
Die zur Behandlung von Zuckerkrankheit und Übergewicht eingesetzten Medikamente Glucagon-like Peptide 1 (GLP-1) Analoga, welche die Wirkung des körpereigenen Darmhormons GLP-1 imitieren, sind für ihren Appetit zügelnden Effekt bekannt. Neuste Resultate aus Tierstudien weisen darauf hin, dass GLP-1 bei verschiedensten Suchterkrankungen (wozu letztlich auch die Adipositas gehört) eine Rolle spielt: das durch Suchtmittel (z.B. Alkohol, Kokain, Nikotin) aktivierte Belohnungssystem scheint durch GLP-1 moduliert zu werden. Wir vermuten deshalb, dass eine Therapie mit GLP-1 Analoga die Entzugserscheinungen nach Rauchstopp zu lindern vermag.
Des Weiteren nehmen wir an, dass bekannte gewichtsreduzierende Effekte von GLP-1 Analoga in der Phase der Rauchentwöhnung ebenfalls besonders vorteilhaft sein werden; damit könnte die typischerweise mit dem Rauchstopp vergesellschaftete Gewichtszunahme reduziert oder gänzlich verhindert werden.
Ein dritter Aspekt macht GLP-1 Analoga zu einer vielversprechenden Behandlungsmöglichkeit: obwohl Rauchen an sich mit einem Risiko für die Entwicklung eines Diabetes mellitus vergesellschaftet ist, erhöht auch der Nikotinstopp das Risiko kurzfristig. Dies könnte durch die blutzuckersenkende Wirkung der GLP-1 Analoga positiv beeinflusst werden. Zusammenfassend vermuten wir, dass die Therapie mit GLP-1 Analoga zur Rauchentwöhnung sowohl die Chance auf einen erfolgreichen Rauchstopp erhöht als auch das kardiovaskuläre Risiko günstig beeinflusst.

ABBREVIATIONS

ACTH	Adrenocorticotropic hormone
AE	Adverse event
AOBPM	Automated Office Blood Pressure Measurement
BD	Blood pressure
BMI	Body mass index
CA	Competent Authority (e.g. Swissmedic)
CEC	Competent Ethics Committee
ClinO	Ordinance on Clinical Trials in Human Research *(in German: KlinV, in French: OClin)*
CO	Carbon monoxide
CRF	Case Report Form
CTCAE	Common terminology criteria for adverse events
CTU	Clinical Trial Unit
DSUR	Development safety update report
eCRF	Electronic Case Report Form
ER	Entity-relationship modelling
GCP	Good Clinical Practice
GLP-1	glucagon-like peptide-1
Ho	Null hypothesis
H1	Alternative hypothesis
hCG	Human chorionic gonadotropin
HFG	Humanforschungsgesetz (Law on human research)
HMG	Heilmittelgesetz
HPA axis	Hypothalamic-pituitary-adrenal axis
HR	Heart rate
HRA	Federal Act on Research involving Human Beings
IB	Investigator's Brochure
ICF	Informed Consent Form
IIT	Investigator-initiated Trial
IMP	Investigational Medicinal Product

ISO	International Organisation for Standardisation
ITT	Intention to treat
KlinV	Verordnung über klinische Versuche in der Humanforschung *(in English: ClinO, in French OClin)*
LPTh	Loi sur les produits thérapeutiques
LRH	Loi fédérale relative à la recherche sur l'être humain
MD	Medical Device
NRT	Nicotine Replacement Therapy
OClin	Ordonnance sur les essais cliniques dans le cadre de la recherche sur l'être humain *(in German : KlinV, in English : ClinO)*
PI	Principal Investigator
PP	Primary polidypsia
QoL	Quality of life
QSU-G	Questionnaire of smoking urge, german version
s.c.	Subcutaneously
SCS	Smoking cessational service
SDV	Source Data Verification
SOC	Standard of Care
SOP	Standard Operating Procedure
SPC	Summary of product characteristics
SUSAR	Suspected Unexpected Serious Adverse Reaction
TMF	Trial Master File
VAS	Visual analogue scale

STUDY SCHEDULE

**Study procedure**	**Screening**	**Week 0**	**Week 1**	**Week 2**	**Week 3**	**Week 4**	**Week 5**	**Week 6**	**Week 7**	**Week 8**	**Week 9**	**Week 10**	**Week 11**	**Week 12**	**Week 24**	**Week 52**	**End of study visit**
**Study Visit**		**V0**				**V4**				**V8**				**V12**	**V13**	**V14**	
Eligibility assessment	X	X															
Informed consent	X	X															
Medical history	X	X															
Inclusion/randomization		X															
Smoking status		X	X	X	X	X	X	X	X	X	X	X	X	X	X	X	X
VAS smoking urge (0-10)		X	X	X	X	X	X	X	X	X	X	X	X	X	X	X	X
QSU-G		X				X								X			
Motivation		X												X		X	X
Alcohol consumption/noxae		X												X		X	X
Vital signs (AOBPM)		X				X				X				X	X	X	X
Weight, height, BMI		X				X				X				X	X	X	X
Laboratory testing		X												X	X	X	X
CO-measurment	X	X	X	X	X	X	X	X	X	X	X	X	X	X	X	X	X
U-cotinine-measurement		X												X	X	X	X
Study drug		X	X	X	X	X	X	X	X	X	X	X	X				
Varenicline		X	X	X	X	X	X	X	X	X	X	X	X				
New Medication			X	X	X	X	X	X	X	X	X	X	X	X	X	X	X
Adverse events			X	X	X	X	X	X	X	X	X	X	X	X	X	X	X
**Substudies**																	

fMRI		X												X			
Indirect calorimetry (REE)		X												X			
Body impedance analysis		X												X			
Serum catecholamines		X												X			

1. STUDY ADMINISTRATIVE STRUCTURE

1.1 Sponsor, Sponsor-Investigator

Prof. Mirjam Christ-Crain
Endokrinologie, Diabetologie und Metabolismus
Universitätsspital Basel, Petersgraben 4, 4031 Basel
Tel: 061 328 70 80, mirjam.christ@usb.ch

1.2 Principal Investigator

Dr. med. Bettina Winzeler
Endokrinologie, Diabetologie und Metabolismus
Universitätsspital Basel, Petersgraben 4, 4031 Basel
Tel: 061 556 50 75, bettina.winzeler@usb.ch

1.3 Statistician ("Biostatistician")

Dr. Tobias Erlanger
Clinical Trial Unit (CTU) Basel
Universitätsspital Basel

1.4 Clinical Epidemiologists

Dr. med. Lars G. Hemkens, MPH
Dr. Benjamin Speich
Basel Institute for Clinical Epidemiology and Biostatistics
Universitätsspital Basel

1.5 Laboratory

Zentrallabor Universitätsspital Basel

Universitätsspital Basel, Petersgraben 4, 4031 Basel

1.6 Monitoring institution

CTU Basel

Universitätsspital Basel

1.7 Data Safety Monitoring Committee

Not applicable

1.8 Authors' contributions

Authors contributions to study protocol: BW wrote the study protocol. BW and TB created the study concept and design. MCC, NJ, AM and FM provided input to study design and drafted the manuscript. TE drafted the statistical analysis section. LGH and BS critically reviewed the protocol and made important contributions on methodological issues. All authors were involved in the critical revision of the protocol for important intellectual content. MCC supervised the study protocol process.

Substudy Energy: NJ and BW wrote the protocol. TB drafted the statistical section. MCC and Matthias Betz (MB) reviewed the protocol and made important contributions.

Substudy fMRI: NJ, BW and Davide Zanchi (DZ) wrote the protocol. TB drafted the statistical section. MCC and Stefan Borgwardt (SB) reviewed the protocol and made important contributions.

2. ETHICAL AND REGULATORY ASPECTS

Before the study will be conducted, the protocol, the proposed patient information and consent form as well as other study-specific documents are submitted to a properly constituted Competent Ethics Committee (CEC), Ethikkommission Nordwest und Zentralschweiz (EKNZ) and Swissmedic.

The decision of the EKNZ and Swissmedic concerning the conduct of the study will be made in writing to the Sponsor-Investigator before commencement of this study. The clinical study can only begin once approval from all required authorities has been received. Any additional requirements imposed by the authorities shall be implemented.

2.1 Study registration

The study will be registered at www.clinicaltrials.gov and the Swiss National Clinical Trials Portal (SNCTP) on www.kofam.ch.

2.2 Categorisation of study

Category B: This is a placebo-controlled study. Dulaglutide is authorized in Switzerland for treatment of diabetes mellitus but is not used in non-diabetics.

2.3 Competent Ethics Committee (CEC)

The responsible investigator at each site ensures that approval from an appropriately constituted Competent Ethics Committee (CEC) is sought for the clinical study.
All changes in the research activity and all unanticipated problems involving risks to humans (including in case of planned or premature study end and the final report) are reported within the allowed time frame to the CEC and no changes are made to the protocol without prior Sponsor and CEC approval, except where necessary to eliminate apparent immediate hazards to study participants.
Premature study end or interruption of the study is reported within 15 days. The regular end of the study is reported to the CEC within 90 days, the final study report is submitted within one year after study end.

2.4 Competent Authorities (CA)

The Sponsor will obtain approval from the competent authority (Swissmedic) before the start of the clinical trial. Premature study end or interruption of the study is reported within 15 days to CA. The regular end of the study is reported to the CA within 90 days, the final study report is submitted within one year after study end. Amendments are reported according to chapter 2.10. Non-substantial amendments are reported as soon as possible.

2.5 Ethical Conduct of the Study

The study will be carried out in accordance to the protocol and with principles enunciated in the current version of the Declaration of Helsinki, the guidelines of Good Clinical Practice (GCP) issued by ICH, the Swiss Law and Swiss regulatory authority's requirements. The CEC and regulatory authorities will receive annual safety and interim reports and be informed about study stop/end in agreement with local requirements.

2.6 Declaration of interest

This is an investigator driven study, there are no conflicts of interest.

2.7 Patient Information and Informed Consent

The investigators (or his designee) will explain to each participant the nature of the study, its purpose, the procedures involved, the expected duration, the potential risks and benefits and any discomfort it may entail. Each participant will be informed that the participation in the study is voluntary and that he/she may withdraw from the study at any time without giving any reasons and that withdrawal of consent will not affect his/her subsequent medical assistance and treatment.
The participant is informed that his/her medical records may be examined by authorised individuals other than their treating physician.
All participants for the study will be provided a participant information sheet and a consent form describing the study and providing sufficient information for participants to make an informed decision about their

participation in the study (see Appendix 1). Enough time is given to the participant to decide whether to participate or not.
The patient information sheet and the consent form will be submitted to the CEC and to the competent authority to be reviewed and approved. The formal consent of a participant, using the approved consent form, must be obtained before the participant is submitted to any study procedure.
The participant should read and consider the statement before signing and dating the informed consent form, and is given a copy of the signed document. The consent form is also signed and dated by the investigator (or his designee) and it will be retained as part of the study records.

2.8 Participant privacy and confidentiality

The investigator affirms and upholds the principle of the participant's right to privacy and that they shall comply with applicable privacy laws. Especially, anonymity of the participants is guaranteed when presenting the data at scientific meetings or publishing them in scientific journals.
Individual subject medical information obtained as a result of this study is considered confidential and disclosure to third parties is prohibited. Subject confidentiality will be further ensured by utilising subject identification code numbers to correspond to treatment data in the computer files.
For data verification purposes, authorised representatives of the Sponsor, Swissmedic or EKNZ may require direct access to parts of the medical records relevant to the study, including participants' medical history.

2.9 Early termination of the study

The Sponsor-Investigator (and any competent authority) may terminate the study prematurely according to certain circumstances, for example:

- ethical concerns,
- insufficient participant recruitment,
- when the safety of the participants is doubtful or at risk, respectively,
- alterations in accepted clinical practice that make the continuation of a clinical trial unwise,
- early evidence of benefit or harm of the experimental intervention.

2.10 Protocol amendments

The Sponsor-Investigator and the Principal Investigators are allowed to amend the protocol or to provide suggestions for a protocol amendment. Substantial amendments are only implemented after approval of the CEC and CA respectively.

Under emergency circumstances, deviations from the protocol to protect the rights, safety and well-being of human subjects may proceed without prior approval of the sponsor and the CEC/CA. Such deviations are documented and reported to the sponsor and the CEC/CA as soon as possible.
All non-substantial amendments are communicated to the CA as soon as possible and to the CEC within the Annual Safety Report (ASR).

3. BACKGROUND AND RATIONALE

3.1 Background and Rationale

Smoking cessation needs to be a health priority as cigarette smoking is the leading preventable cause of premature death[1]. Although most of smokers say that they wish to quit, one year quit rates after an unaided attempt are very low (3-6 %)[2,3]. If smokers participate in a smoking cessation program making use of the most effective treatment - a combination of behavioural and pharmacotherapy - abstinence rates after one year are higher, but still unsatisfactory according to two systematic reviews (around 30%)[4,5].
There are several difficulties that smokers encounter when they try to quit. As nicotine is a potent psychoactive drug causing physical dependence[6], withdrawal symptoms (e.g. craving for cigarettes, changes in mood) peaking in the first weeks put patients on risk for an early relapse.
Another important barrier represents the post-cessational weight gain[7]. On average smoking quitters show an increase in mean body weight of 4-5 kg within 12 months[8-10]. Current pharmacotherapy for smoking cessation (e.g. nicotine replacement therapy (NRT), varenicline and bupropion) has not been shown to reduce post-cessational weight gain[10,11].
Although smoking per se is a risk factor for incident type 2 diabetes[12], smoking cessation increases short

term risk of diabetes[13]. Besides post-cessational weight gain, several other potential causal factors are discussed (e.g. systemic inflammation)[14,15].

Taken together, to maximise smoking cessation, novel strategies should address both: nicotine withdrawal syndrome and unfavourable metabolic effects of smoking cessation especially weight gain, which is often reported as the main reason for not quitting or relapsing.

The gut-brain hormone GLP-1 is released from endocrine cells from the distal gut and centrally by neurons in the nucleus of the solitary tract of the hindbrain in response to food entry in the gastrointestinal tract[16-18] [19-21]. GLP-1 receptors are expressed in several brain areas, including the hypothalamus and the reward nodes ventral tegmental area and nucleus accumbens[20,22], implicating a role of GLP-1 in reward regulation[23].

Due to its well-known insulinotropic and satiation-promoting effects, GLP-1 analogues are widely used as a treatment of type 2 diabetes and obesity. GLP-1 might not only play a role in regulating appetite and energy intake (probably via food-related reward processing), but also in controlling reward induced by addictive drugs such as alcohol, amphetamine or nicotine[24,25]. A recent study found that treatment with the GLP-1 analogue exendin-4 attenuated nicotine-induced effects on the mesolimbic dopamine system in mice[23], which is thought to be responsible for the development of addiction and compulsive drug taking[26].

Thus, GLP-1 analogues might alleviate symptoms of the nicotine withdrawal syndrome in humans, thereby making it easier for smokers to stop. Furthermore, as mentioned above, both the weight reducing effect and the anti-diabetogenic properties of GLP-1 analogues are of particular relevance in the context of smoking cessation. GLP-1 analogues seem a very promising novel therapy for smoking cessation targeting withdrawal symptoms on the one hand and preventing weight gain and diabetes on the other.

3.2 Investigational Product (treatment, device) and Indication

TRULICITY contains dulaglutide, which is a human GLP-1 receptor agonist with 90% amino acid sequence homology to endogenous human GLP-1 (7-37). Dulaglutide activates the GLP-1 receptor, a membrane-bound cell-surface receptor coupled to adenylyl cyclase in pancreatic beta cells. Dulaglutide increases intracellular cyclic AMP (cAMP) in beta cells leading to glucose-dependent insulin release. Dulaglutide also decreases glucagon secretion and slows gastric emptying. The overall molecular weight of dulaglutide is approximately 63 kilodaltons. TRULICITY is indicated as an adjunct to diet and exercise to improve glycemic control in adults with type 2 diabetes mellitus.

TRULICITY is a clear, colorless, sterile solution. Each 0.5 mL of TRULICITY solution contains 1.5 mg of dulaglutide. Each single-dose pen contains 0.5 mL of solution and the following excipients: citric acid anhydrous (0.07 mg), mannitol (23.2 mg), polysorbate 80 (0.10 mg), trisodium citrate dihydrate (1.37 mg), in water for injection[27].

3.3 Preclinical Evidence

There is growing literature indicating that GLP-1 is involved in the pathophysiology of addiction by mediating reward induced by several substances (e.g. alcohol, amphetamine, nicotine)[24]. Initial publications focused primarily on alcohol addiction and showed that the GLP-1 analogues exendin-4 and liraglutide attenuates the reinforcing properties of alcohol measured by locomotor stimulation and reduces alcohol intake in rodents[28-30]. Similary, exendin-4 seems to prevent as well rewarding properties of amphetamine and cocaine[31-33].

Results from a recent animal study are consistent with the hypothesis that GLP-1 regulates nicotine-induced effects on the mesolimbic dopamine system in mice[23], which is thought to be responsible for the development of addiction and compulsive drug taking[26].

3.4 Clinical Evidence to Date

Besides preclinical data suggesting a role of GLP-1 and -analogues in reward regulation, there is growing indirect evidence that GLP-1 receptors may be pharmacological targets for addictive diseases in humans: Decreased alcohol intake in both rats and humans has been observed after gastric bypass surgery – a procedure well known to increase GLP-1 levels[34]. Furthermore, one study reported an association of genetic variation in GLP-1 receptors and alcohol use disorders[35]. So far, there is no human evidence available showing a role of GLP-1 in nicotine dependency. Currently, there is one ongoing experimental study looking at addictive behaviour under exenatide treatment assessed by functional MRI in dependent individuals who have recently stopped smoking or drinking alcohol (NCT02690987) and one ongoing randomized controlled trial evaluating the effects of the GLP-1 analogue exenatide (Bydureon®) on smoking cessation (NCT02975297). The study design of the latter trial differs from ours, being more

explorative: the experimental product (exenatide) is given for 6 weeks only on top of NRT and quit-rates are assessed weekly during a period of 10 weeks. The planned sample size is smaller (90 participants) and inclusion criteria are more restrictive focusing only on overweight or prediabetic smokers.

3.5 Dose Rationale / Medical device: Rationale for the intended purpose in study (pre-market MD)

Dulaglutide 1,5 mg is injected once a week according to the dosage recommended for treatment of diabetes mellitus. The higher of the two available treatment doses (0.75 and 1.5 mg) is chosen in order to achieve a higher effect.

3.6 Explanation for choice of comparator (or placebo)

To causally attribute the effect to dulaglutide (and not to the interventional setting) and prove that the agent modulates smoking cessation, we choose a placebo-controlled design for the study.

3.7 Risks / Benefits

The GLP-1 analogue dulaglutide is currently used as an antidiabetic treatment in type 2 diabetics at doses of 0.75 and 1.5 mg once weekly and has a favourable safety profile. In several phase 3a trials of the AWARD program (the Assessment of Weekly AdministRation of LY2189265 in Diabetes)[36-39] dulaglutide has been evaluated in totally 4572 patients with type 2 diabetes. In these clinical trials patients were initiated and maintained on either 0.75 or 1.5 mg once a week, without titration algorithms.
Predominant adverse effects were gastrointestinal symptoms with nausea (up to 20%) as the largest component. The gastrointestinal symptoms – peaking during the first two weeks of treatment - were usually mild to moderate and mostly transient. E-R modelling from pooled clinical pharmacology data found no significant difference in nausea events between fixed dosing (1.5 mg weekly) and a titrated regimen (0.75 mg initial dose followed by 1.5 mg)[40]. In the trials of the AWARD program no severe hypoglycaemia occurred under monotherapy with dulaglutide, documented symptomatic hypoglycaemia in dulaglutide treated diabetic patients (1.5 mg/week) was rare (0.19-0,62 episodes/patient/year). Similarly, hypoglycemia was no relevant issue when another GLP-1 analogue (liraglutide) was evaluated in 2487 non-diabetic obese patients (spontaneous, not severe, hypoglycaemia reported by 1.3% versus 1.0% patients treated with liraglutide and placebo, respectively)[41]. Liraglutide is now approved for weight management in the USA and EU.

Besides its glucose and weight lowering properties, GLP-1 analogues exert benefits on further cardiovascular risk factors such as blood pressure and dyslipidemia. There is growing literature from large-scale clinical trials showing improved cardiovascular outcome associated with different GLP-1 analogue treatment[42]. Especially for liraglutide and semaglutide a significant cardiovascular risk reduction was shown in diabetic patients with high cardiovascular burden[43,44].
For dulaglutide cardiovascular outcome data are awaited from the Researching Cardiovascular Events with a Weekly Incretin in Diabetes (REWIND) study estimated to be completed in 2018 (NCT01394952). Approximately 9000 type 2 diabetic patients with established cardiovascular disease are randomized to 1.5 mg dulaglutide or placebo in addition to standard of care during an average follow-up of 6.5 years. The primary end point includes time to first occurrence of a three component MACE (including CV death, nonfatal myocardial infarction, or nonfatal stroke).

There are no major safety concerns with the trial product dulaglutide, thus we consider the risks of this study to be minor.
During the study period possible adverse events are assessed on each study visit. In case of adverse events or other concerns throughout the entire study period participants can contact the study team during office hours or visit the emergency department of the University Hospital Basel at any time.

3.8 Justification of choice of study population

Adult smokers will participate in the study after thorough information and providing written informed consent. This study population has been chosen because the aim of the study is to offer a novel treatment to smokers who wish to quit.

4. STUDY OBJECTIVES

4.1 Overall Objective

The overall objective of the study is to evaluate the GLP-1 analogue dulaglutide (Trulicity®) as a new therapeutic drug for smoking cessation assessing abstinence rate at 3 months of dulaglutide treatment.

4.2 Primary Objective and Hypothesis

The primary objective is to assess the influence of dulaglutide combined with standard therapy (varenicline and behaviour counselling) for smoking cessation on smoking quit rates after 12 weeks compared to standard therapy alone.
Question to be answered: Has dulaglutide an effect on smoking quit rates (presumably by reducing symptoms of the nicotine withdrawal syndrome and preventing post-cessational weight gain) when combined with standard therapy (i.e. SCS, daily varenicline) of smoking cessation?
Hypothesis: The success rate of smoking cessation under standard therapy is 33 % at three months. Dulaglutide treatment increases smoking quit rate (week 12) by 18% compared to standard therapy alone.
These hypotheses are based on the following data evidence and assumptions:

- Results of a recent smoking cessation trial evaluating the efficacy of a 3 months treatment with varenicline and cessation counselling in more than 2000 patients showed a continuous abstinence rate of 33.5% after 12 weeks[45]. In a retrospective analysis of all patients consulting our own smoking cessation service (SCS) between 2012 and 2013, the abstinence rate under standard therapy at 3 months was 36% (own unpublished data; counting patients with missing outcome data as smokers). In this analysis, smoking abstinence was self-reported and not biochemically validated by CO measurement. Thus, we assume that the biochemically validated abstinence rate (week 12) will be slightly lower (approximately 33%).
- The effect of varenicline on smoking cessation might be increased by adding a second component of medical therapy. The assumption of an additional increase by 18 % is clinically relevant. We presume that the effect combining varenicline with dulaglutide will be in a similar range as found in the largest available RCT combining varenicline with nicotine replacement therapy (NRT) that is included in a recent meta-analysis of the three available randomized trials[46]. In this trial, the additional increase at 3 months was 14.5%. The summary effect across the three trials in the meta-analysis showed an increased odds of 50% (OR 1.50; 95% CI 1.14 to 1.97) to quit smoking early when NRT was combined with varenicline as second component of medical therapy. Given the potential additional weight reducing effect of GLP-1 analogues we presume a somewhat better effect of dulaglutide compared to NRT, supporting our assumption of an 18% absolute increase.

4.3.1 Main Secondary Objective

The main secondary objective is to assess body weight change after smoking cessation under dulaglutide treatment and standard therapy (varenicline and behaviour counselling) compared to standard therapy alone.
Question to be answered: Can dulaglutide reduce or even avoid weight gain, which is typically seen after smoking cessation?
Hypothesis: Three months treatment with dulaglutide reduces post-cessational weight gain assessed at 12 weeks compared to standard therapy alone.
This hypothesis is based on the following data and considerations:

- Mean post-cessational weight gain has been shown to be approximately 4 kg over 5 years in a 2015 meta-analysis of observational studies[47]
- The majority of weight gain seems to occur during the first months and year[48], being as high as 2.3 kg after two months[10]
- A similar absolute weight reduction as seen in diabetic patients (-2.3 kg after 26 weeks of dulaglutide treatment[37]) is expected in participants receiving dulaglutide who do not quit smoking

4.3.2 Further Secondary Objectives

Effect of dulaglutide versus placebo on:

- Point prevalence abstinence at 24 and 52 weeks
- Prolonged abstinence at 24 and 52 weeks (requires abstinence at week 12 and at week 12+24, respectively)
- Short-term (12 weeks) and long-term (24 and 52 weeks) smoking reduction
- Nicotine craving, especially in the early phase of smoking cessation (4 weeks) and at week 12 relative to baseline
- Change of body weight at 4, 8, 24 and 52 weeks
- Glucose homeostasis at 12, 24 and 52 weeks
- Resting Energy Expenditure (see Substudy energy Appendix 4)
- Functional magnetic resonance imaging (fMRI) brain activity (see Substudy fMRI Appendix 5)

4.4 Safety Objectives

As dulaglutide is already in use as antidiabetic treatment, safety objectives regarding an investigational treatment do not apply. However the study assesses tolerability of dulaglutide in smokers who are not explicitly diabetic.

5. STUDY OUTCOMES

5.1 Primary Outcome

Point prevalence abstinence rate at week 12 of dulaglutide treatment combined with standard therapy versus standard therapy alone, confirmed with end-expiratory exhaled carbon monoxide (CO) measurements of 10 ppm or less.

Point prevalence abstinence is assessed at week 12 (within a ± 7days time window) and defined as:

- Self-reported 7-days nicotine abstinence **and**
- End-expiratory exhaled carbon monoxide (CO) measurement of 10 ppm or less

5.2 Secondary Outcomes

Main secondary outcome: Change of body weight in kg (and BMI [kg/m²]) at week 12 relative to baseline.
Further outcomes:

- Point prevalence abstinence at week 24 and 52 (self-reported 7 days abstinence), biochemically validated by end-expiratory exhaled CO (10 ppm or less)
- Prolonged abstinence at week 24 and 52, defined by the condition that the patient was already previously abstinent (i.e. at week 12 for prolonged abstinence at week 24 and at week 12 and 24 for prolonged abstinence at week 52)
- Smoking reduction defined as reduction of end-expiratory exhaled CO more than 50% at week 12, 24 and 52 compared to baseline
- Craving at week 4 and 12 relative to baseline assessed by the German version of the "Questionnaire on Smoking urges" (QSU-G) and a visual analogue scale (VAS)
- Change of body weight in kg (and BMI [kg/m²]) at week 4, 8, 24 and 52 relative to baseline
- Change in haemoglobin A1c (HbA1c) in percent at week 12, 24 and 52 relative to baseline

5.3 Safety Outcomes

During the study period possible adverse events are assessed on each visit.

6. STUDY DESIGN

6.1 General study design and justification of design

This is a placebo-controlled, double-blind, parallel group, superiority, single-center randomized trial.

- The estimated sample size is 256 adult smokers.
- Participants are randomly allocated (1:1 ratio) to treatment with dulaglutide or placebo.
- The 12-week treatment phase with dulaglutide or placebo is followed by a 40-week non-treatment

phase.

- Participants, health-care providers and data collectors are blinded except unblinded study staff doing the injection of the trial drug (see below).
- The primary outcome is measured after 12 weeks of treatment with dulagultide 1.5 mg weekly or placebo.
- The experimental drug is given on top of standard of care, which include behaviour counselling and pharmacological treatment with varenicline.

This is a proof of concept study evaluating GLP-1 analogues as a novel treatment option for smoking cessation. It focuses on mechanistic properties of this new potential indication, why the primary outcome is assessed at the end of the treatment phase of 12 weeks, when effects are likely to be visible. In case of a positive result a larger multicentre trial addressing the clinically more relevant question of long-term abstinence under GLP-1 analogues will be designed.
According to national guidelines ("Nationales Rauchstopp-Programm")[49], a pharmacological treatment with varenicline, NRT or bupropion is recommended in smokers with moderate to severe nicotine dependency. As varenicline has been shown to be the most efficient pharmacological treatment[5], this drug has been chosen as standard of care for this trial. Even though there is some randomized trial evidence suggesting benefit by adding NRT to varenicline[46], this combination has not been adopted in current national guidelines and is not reimbursed by health insurers in Switzerland. Moreover, NRT lacks any of the potential additional benefits on body weight changes.

6.2 Methods of minimising bias
6.2.1 Randomisation

After successful screening, participants are assigned to a sequentially numbered study ID. By activating the study ID in the electronic data capture system (secuTrial®), participants are randomized (1:1 ratio, simple randomization) to be given dulaglutide and placebo according to a computer generated randomisation list produced by the CTU Basel.

6.2.2 Blinding procedures

Participants, health-care providers and data collectors are blinded to treatment allocation. As injection devices of dulaglutide and placebo are not identical, there is unblinded study staff doing the injections. The unblinded study staff is otherwise **not** involved in the trial. During the trial drug injection, participants wear a blindfold and are not able to see the injection side or the injection device. This process is conducted and documented according to the respective SOP (trial drug application by an unblinded person) of the CTU Basel.

6.2.3 Unblinding Procedures (Code break)

In emergency situations, e.g. adverse events, and up to the decision of the investigator if medically important, it is allowed to break the blind of the participant and to reveal the codes in the sealed data system.

7. STUDY POPULATION

7.1 Eligibility criteria

Inclusion criteria:

- Age 18 to 75 years
- Daily smokers who are willing to quit <u>and</u> exhibit one of the following criteria:
- ≥10 cigarettes per day <u>**or**</u>
- At least moderate nicotine dependence defined by a Fagerstroem Score of ≥5 points <u>**or**</u>
- Tobacco associated disease
- Treatment with varenicline (Champix®)

Exclusion criteria:

- Pregnancy (incl. wish to become pregnant within next 3 months) or breast feeding
- Pre-existing treatment with GLP-1 agonists
- History of pancreatitis

- Severe renal insufficiency (estimated glomerular filtration rate <30 ml/min/1,73 m^2)
- Instable psychiatric conditions
- Anorexia nervosa

Diabetes mellitus is not an exclusion criterion. Included patients with diabetes are treated by their private doctors; during the study period all kinds of anti-diabetic drugs can be used except GLP-1 analogues.

7.2 Recruitment and screening

At the Medical Outpatient Department of the University Hospital Basel a standardized smoking cessational service (SCS) was established in 2012. Under the lead of a cardiologist (TB) and a general internist (AM) advice and treatment is offered to all patients willing to quit smoking. Treatment consists of the combination of individual advice, behavioural support and pharmacotherapy according to current national guidelines for smoking cessation (Nationales Rauchstopp-Programm, www.frei-von-tabak.ch)[49].
The investigators will recruit potential participants primarily in this SCS. In 2016 four new smokers willing to quit were seen in the SCS per week. According to internal experience around 80% would meet eligibility criteria of this trial. We assume that 50% of currently treated patients in the SCS would be willing to participate (thus, we plan to recruit approximately 2 patients per week and 100 per year).
As the SCS is still expanding and there is capacity to see more patients, potential candidates are also searched by advertisements on the homepage of the University of Basel https://markt.unibas.ch/inserate/kategorie/job-angebot-studien, on the facebook website of the University Hospital of Basel https://www.facebook.com/unispitalbasel/ and by announcement in different print journals. Interested participants contact the study investigators by email or telephone and will subsequently receive the ICF via mail or e-mail. Willing to participate, they contact the investigators and an appointment in the SCS is arranged.
The screening requirements include a clinical evaluation with assessment of the Fagerstroem Score. In patients with a history of renal insufficiency a basal determination of creatinine is performed prior to inclusion.
All study visits will be conducted at the study centre of the University Hospital of Basel (Ambulantes Studienzentrum), which is located within the hospital area and close to the SCS.

The costs for the medical and psychosocial care (behavioural counselling) provided by the smoking cessational service is covered by the patient’s health insurance. The trial medication is offered from the study team for free. Participants completing the study according to protocol will receive CHF 150.- at the last visit. They receive an additional CHF 50.- compensation if both follow-up visits (after 6 and 12 months) are completed.

7.3 Assignment to study groups

Participants are assigned to a sequentially numbered study ID. By activating the study ID in the electronic data capture system (secuTrial®), participants are assigned to a treatment arm (dulaglutide and placebo) according to a computer generated randomization list produced by the CTU Basel. Investigators and involved study staff are informed by email if a new participant is enrolled. However only members of the unblinded study staff have access to the study group assignment of enrolled participants.

7.4 Criteria for withdrawal / discontinuation of participants

Participants can at any time and without reasons withdraw their informed consent to the study.

8. STUDY INTERVENTION

8.1 Identity of Investigational Products (treatment / medical device)
8.1.1 Experimental Intervention (treatment / medical device)

Dulaglutide injection 0.75 mg in 0.5 ml via Pen (only first injection)

Dulaglutide, injection 1.5 mg in 0.5 ml, via Pen (second to twelfth injection)

8.1.2 Control Intervention (standard/routine/comparator treatment / medical device)

0.5 ml normal saline (0.9% sodium chloride [0.9% NaCl]), injection via syringe.

8.1.3 Packaging, Labelling and Supply (re-supply)

Prior to the initiation of the study the drugs are labelled by an unblinded study person (CTU Basel).

8.1.4 Storage Conditions

Investigational products are kept in a secure, limited access storage area under the recommended storage conditions.

8.2 Administration of experimental and control interventions

The study drug (experimental or control) is injected s.c. once weekly by unblinded study staff at the study centre of the University Hospital Basel.

8.2.1 Experimental Intervention

Dulaglutide weekly s.c. injection of 0.75 mg/0.5 mL (first injection) and 1.5 mg/0.5 mL (second to twelfth injection) in the abdomen or thigh (total treatment duration: 12 weeks).

8.2.2 Control Intervention

Placebo weekly s.c. injection of 0.5 mL in the abdomen or thigh (total treatment duration: 12 weeks).

8.3 Dose / Device modifications

During the study period all participants are on a concomitant treatment with varenicline (=SOC). As varenicline may also produce gastrointestinal (GI) side effects (nausea [≥1/10], other GI symptoms [≥1/100, <1/10]), all patients in the smoking cessational service are generally instructed to ingest varenicline together with a meal and to use a PPI if needed – in order to obtain an optimal tolerability.
If a study participant complains gastrointestinal side effects at any time, investigators proceed as follows:

1. Remind participant to ingest varenicline together with a meal
2. Prescription of a proton pump inhibitor (PPI)
3. Dose reduction of the study drug
4. Dose reduction of varenicline

8.3.1 Dose reduction of the study drug

Given the transient nature of gastrointestinal side effects of dulaglutide, the standard dose of 1,5 mg is given after the first injection – whenever possible. In case of persisting gastrointestinal side effects, the investigator may decide at any time whether treatment dosage should be reduced to 0.75 mg/0.5 ml dulaglutide (or 0.5 ml of 0.9% NaCl). The unblinded study person is instructed to conduct a dose reduction if possible (=patient on verum) while the involved study team and the patient remain blinded. If intolerable side effects persist after dose reduction, investigators decide whether the dose of varenicline should be reduced. If side effects resolve, investigators decide whether the dose should again be increased during the remaining study period.

8.3.2 Dose reduction of varenicline

If gastrointestinal symptoms persist after reduction of the study drug, investigators may consider at any time a dose reduction of varenicline (standard dose: 1 mg twice daily, reduction to 0.5 mg twice daily or 0,5 mg once daily). If side effects resolve, investigators decide whether the varenicline dose should again be increased during the remaining study period. If intolerable side effects persist after dose reduction of the study drug and varenicline the participant will not continue with the allocated intervention but remains under follow-up.

8.4 Compliance with study intervention

Participants not presenting to the planned study visits are contacted by phone and a new appointment is arranged. The study drug is usually injected once weekly on a fixed weekday, but a time span of ±3 days is allowed (=one injection/week, independent of the day). If participants do not present within this time span,

the investigator decides if the visit is postponed by maximally one week or skipped. On reciprocal agreement smoking status incl. CO measurement and urinary cotinine can be assessed at a home visit.

8.5 Data Collection and Follow-up for withdrawn participants

For withdrawn patients an End of Study Visit is arranged in order to complete clinical data and assess possible AEs. Already collected data and biological material of withdrawn participants will be analysed and afterwards made anonymous. Patients have the right to let delete their data after study exclusion. If participants agree, they are contacted at week 12, 24 and 52 and smoking status is assessed. Whenever possible, self-reported smoking abstinence is biochemically validated by CO-measurement and urinary cotinine measurement. These tests can be assessed during a home visit.

8.6 Trial specific preventive measures

During the whole study period, use of other GLP-1 analogues (e.g. liraglutide) is not allowed.

8.7 Concomitant Interventions (treatments)

Whenever possible, any additional treatment during study period should be avoided. If concomitant medication is strongly recommended, the investigator decides whether the study can be continued. During the whole study period NRT are not allowed.

8.8 Study Drug / Medical Device Accountability

From shipment to the site until return or disposal study drugs are accurately and adequately monitored. Dates of receipt/expiry/use/return are recorded.

8.9 Return or Destruction of Study Drug / Medical Device

During the whole study period and at the end of the study the product is kept by the Sponsor.

9. STUDY ASSESSMENTS

See Study Schedule

9.1 Assessment of primary outcome

The primary outcome point-prevalence abstinence rate for week 12 is assessed by questionnaire (self-reported 7-days abstinence) <u>and</u> end-expiratory exhaled carbon monoxide measurements of 10 ppm or less at visit 12. The patient is considered smoker if one of these two tests is positive.

9.2 Assessment of main secondary outcome

Body weight in kg (and BMI [kg/m^2]) is assessed at baseline and week 12

9.2.1 Assessment of further secondary outcomes:

9.2.2 Abstinence rate during follow-up phase

Abstinence rate is assessed at week 24 and 52 by:

- Questionnaire (7-days abstinence)
- End-expiratory exhaled carbon monoxide measurements of 10 ppm or less
- Qualitative testing for urinary cotinine (negative result=abstinent, positive result=smoker)

Abstinence is defined as follows:

- Point prevalence abstinence: self-reported 7-days abstinence biochemically confirmed by a CO measurement of 10 ppm or less
- Prolonged abstinence:
- -> at week 24: biochemically confirmed self-reported abstinence at week 12 and 24
- -> at week 52: biochemically confirmed self-reported abstinence at week 12, 24 and 52

9.2.3 Smoking reduction

Smoking reduction is assessed at week 12, 24, 52 by:

- Questionnaire (number of cigarettes per day during the last week)
- End-expiratory exhaled carbon monoxide measurements at week 12, 24 and 52 compared to baseline (a reduction of more than 50% is required)

9.2.4 Craving

Craving is assessed:

- at baseline and week 4 and 12 by the german version of the "Questionnaire of smoking urge" (QSU-G)[50] (see Appendix 2)
- at baseline and week 4 and 12 by a visual analogue scale (VAS), ranging from 0-10 points (no to maximal craving), compromising the last 24 hours (see Appendix 3)

9.2.5 Body weight

- Body weight in kg (and BMI [kg/m²]) is assessed at week 4, 8, 24 and 52

9.2.6 Glucose homeostasis

- Glucose homeostasis is assessed at baseline, week 12, 24 and 52 by measurement of HbA1c

9.2.7 Further explorative outcome measures

- Abstinence assessed by urinary cotinine (qualitative testing) at week 12, 24, 52
- Smoking status and habits incl. end-expiratory exhaled carbon monoxide on every visit
- Smoking Cessation: Motivation and confidence assessed by a self-constructed questionnaire of the SCS at week 0, 12, 24, 52
- Craving assessed by VAS (range 0-10) on every visit
- AOBPM at baseline, week 4, 8 and 12
- Alcohol consumption and other substance abuse assessed by a self-constructed questionnaire at week 0,12, 24, 52

9.2.8 Assessment of safety outcomes

On all study visits while participants receive a dose of dulaglutide/placebo, tolerability of the drug and symptoms (abdominal pain, nausea, vomitus, diarrhoea, local irritation or pain, allergic reaction) are assessed.

9.2.9 Assessments in participants who prematurely stop the study

Withdrawn patients will be asked to complete the following assessments at the end of study (End of Study Visit):
Smoking status incl. biochemical validation
Smoking urge (VAS)
Alcohol and substance abuse by questionnaire
Vital signs (incl. weight and AOBPM)
Blood withdrawal for routine (HbA1c) and specific laboratory measurements

9.3 Procedures at each visit

9.3.1 Screening phase:

The following tests/actions are performed within 8 weeks before or on Visit 0 (see source doc):
Information about the study
Fagerstroem Score
Eligibility Criteria
Blood withdrawal (creatinine) if history of renal insufficiency present

9.3.2 Visit 0, Inclusion

Informed Consent
Demographics
Medical history questionnaire

Blood pressure, heart rate (AOBPM)
Weight, Height, BMI
CO-measurement
Questionnaire for smoking urge (QSU-G)
VAS smoking urge during the last 24 hours
Blood withdrawal
Urinary analysis
Pregnancy test in premenopausal females
Randomization
Scheduling of all other study visits
The first dose of trial medication is injected by an unblinded study person
The medication with varenicline is started the same as visit 0

9.3.3 Treatment phase, visits 2, 3, 4, 5, 6, 7, 8, 9, 10, 11, 12

- The weekly dose of the trial medication is injected
- Tolerability of the drug, adverse events and new medications are assessed
- Dose of the trial drug and varenicline are documented
- Assessment of smoking status by questionnaire and CO measurement
- Assessment of smoking urge by VAS

Additionally:

- Body weight on visits 4,8,12
- AOBPM on visits 4,8,12
- Questionnaire for smoking urge on visits 4,12
- Blood withdrawal on visit 12
- Urinary analysis on visit 12

9.3.4 Follow-up phase, visit 24 (week 24) and Visit 52 (week 52)

- Assessment of adverse events
- Assessment of smoking status
- Assessment of smoking urge by VAS
- Co-measurement
- AOBPM
- Weight
- Blood withdrawal
- Urinary analysis

9.3.5 End of study visit for withdrawn participant

- Assessment of adverse events
- Assessment of smoking status
- Assessment of smoking urge by VAS
- Co-measurement
- AOBPM
- Weight
- Blood withdrawal
- Urinary analysis

9.3.6 Visits within the SCS

The program of the SCS generally includes 4 follow-up visits (e.g. week 2,4,8,12), or more on an individual basis if needed. Treatment consists of a combination of individual advice and behavioural support.

10. SAFETY

During the entire duration of the study, all adverse events (AE) and all serious adverse events (SAEs) are collected, fully investigated and documented in source documents and case report forms (CRF).

10.1 Definition and assessment of (serious) adverse events and other safety related events

An **Adverse Event (AE)** is any untoward medical occurrence in a patient or a clinical investigation participant administered a pharmaceutical product and which does not necessarily have a causal relationship with the study procedure. An AE can therefore be any unfavourable and unintended sign (including an abnormal laboratory finding), symptom, or disease temporally associated with the use of a medicinal (investigational) product, whether or not related to the medicinal (investigational) product. □ICH E6 1.2□

A **Serious Adverse Event (SAE)** is classified as any untoward medical occurrence that:

- results in death,
- is life-threatening,
- requires in-patient hospitalization or prolongation of existing hospitalisation,
- results in persistent or significant disability/incapacity, or
- is a congenital anomaly/birth defect.

Assessment of Causality

Both Investigator and Sponsor-investigator make a causality assessment of the event to the study drug, based on the criteria listed in the ICH E2A guidelines:

Relationship	Description
Definitely	Temporal relationship<br>Improvement after dechallenge*<br>Recurrence after rechallenge<br>(or other proof of drug cause)
Probably	Temporal relationship<br>Improvement after dechallenge<br>No other cause evident
Possibly	Temporal relationship<br>Other cause possible
Unlikely	Any assessable reaction that does not fulfil the above conditions
Not related	Causal relationship can be ruled out
*Improvement after dechallenge only taken into consideration, if applicable to reaction	

Unexpected Adverse Drug Reaction

An “unexpected” adverse drug reaction is an adverse reaction, the nature or severity of which is not consistent with the applicable product information (e.g. Investigator’s Brochure for drugs that are not yet approved and Product Information for approved drugs, respectively). □ICH E2A□

Suspected Unexpected Serious Adverse Reactions (SUSARs)

The Sponsor-Investigator evaluates any SAE that has been reported regarding seriousness, causality and expectedness. If the event is related to the investigational product and is both serious and unexpected, it is classified as a SUSAR.

Assessment of Severity

For this study the severity grading scale “Common Terminology Criteria for Adverse Events CTCAE Version 4.0” is used.

10.2 Reporting of serious adverse events (SAE) and other safety related events

All SAEs are reported immediately and within a maximum of 24 hours to the Sponsor-Investigator of the study. The Sponsor-Investigator will re-evaluate the SAE and return the form to the site.
SAEs resulting in death are reported to the local Ethics Committee (via local Investigator) within 7 days.

Reporting of SUSARs

SUSAR’s are reported to the local Ethics Committee and to Swissmedic (via Sponsor-Investigator) within 7 days, if the event is fatal, or within 15 days (all other events).

Reporting of Safety Signals
All suspected new risks and relevant new aspects of known adverse reactions that require safety-related measures, are reported to the Sponsor-Investigator within 24 hours. The Sponsor-Investigator reports the safety signals within 7 days to the local Ethics Committee and to Swissmedic.
Reporting and Handling of Pregnancies
Participants have to be willing to use contraceptive measures adequate to prevent becoming pregnant. Adequate contraceptive measures include hormonal methods used (e.g., oral contraceptive pills, contraceptive patch, or contraceptive vaginal ring), double barrier methods (e.g. diaphragm used together with contraceptive foam or jelly, and condom used combined with contraceptive foam or jelly), intrauterine methods (IUD) and sterilization (e.g., tubal ligation or a monogamous relationship with a vasectomized partner).
Pregnant participants are immediately withdrawn from the clinical study. Any pregnancy during the treatment phase of the study and within 20 days after discontinuation of study medication will be reported to the Sponsor-Investigator within 24 hours. The course and outcome of the pregnancy is followed up carefully, and any abnormal outcome regarding the mother or the child is documented and reported.

Periodic reporting of safety
An annual safety report is submitted once a year to the local Ethics Committee via local Investigator and to Swissmedic.

10.3 Follow up of (Serious) Adverse Events

Following a comprehensive baseline evaluation, each subject's safety will be monitored with periodic recording and evaluation of all treatment-emergent AEs.
Once evidence of a clinical abnormality is noticed, the condition will be treated while trying to determine its cause. The subject will then be followed until the condition resolves or becomes chronic or stable. Patients will be instructed about possible acute AEs. If adverse events are observed, the patients are promptly treated according to standard of care.

11. STATISTICAL METHODS

11.1 Hypothesis

The Null Hypothesis (H_0) is as follows: point prevalence abstinence rate in smokers treated with dulaglutide in addition to standard of care (varenicline and behavioural counselling) for 12 weeks, is not different from point prevalence abstinence rate when treated with placebo in addition to standard of care.
H_0 will be rejected when point prevalence abstinence rate between dulaglutide in addition to standard of care and placebo in addition to standard of care is significantly different.
For hypothesis testing, an alpha error threshold of 5% will be used.

11.2 Determination of Sample Size

Sample size was estimated using a simulation approach. Each sample size, $n_{i=1,...,201}$ = 100, ..., 500, was evaluated by simulating R = 999 times n_i outcomes as differences in point prevalence abstinence rates in %.
The difference between point prevalence abstinence rates of intervention and control arm follows, presumably, a χ^2 -distribution. To assess the significance between the two point prevalence abstinence rates, a Pearson's χ^2 -test was applied.
It is assumed that efficacy (point prevalence abstinence rates) for the control group is 33% and the increase of effect size under experimental treatment is 18%, i.e. point prevalence abstinence rate increases from 33% to 51%.
For this study, 256 patients should be recruited for both arms. This sample size allows in at least 80% of 999 hypothetical repetitions of the study to show a difference in point prevalence abstinence rates between the two study arms at a significance level, α, of 5%.

11.3 Planned Analyses

11.3.1 Datasets to be analysed

The full analysis dataset (FAS) consists of all patients who are randomised. Following ITT recommendations, they are analysed according to their group of randomisation.
The per protocol dataset (PP) consists of patients in the FAS data set without any major protocol deviations. Major protocol deviations occur if patients receive less than 80% of the trial medication, i.e. less than 10 of 12 possible doses.

11.3.2 Primary Analysis

Primary analysis will assess whether point prevalence abstinence rates between experimental and control arms are significantly different. Since the difference follows a χ^2 distribution, a Pearson's χ^2-test will be applied.

11.3.3 Secondary Analyses

Difference of change of body weight (kg) between the 2 arms will be assessed by ANCOVA. Body weight in kg at week 4, 8, 12, 24, and 52 will be the outcome variable, baseline weight and treatment arm both explanatory variables.
Difference of change of BMI (kg/m^2) between the 2 arms will be assessed by ANCOVA. BMI at week 4, 8, 12, 24, and 52 will be the outcome variable, baseline BMI and treatment arm both explanatory variables.
Differences between arms regarding point prevalence abstinence rates at week 24 and 52 will be calculated like the primary analysis.
Differences between arms regarding prolonged abstinence at week 24 and 52 will be calculated like the primary analysis, except that only patients are considered being abstinent who were so at previous assessments (week 12 for abstinence at week 24 and weeks 12+24 for abstinence at week 52).
Smoking reduction at week 12, 24, and 52 relative to baseline will be analysed as a binomial outcome. Percentage of people who reduced smoking by at least 50% will be compared between arms. A Pearson's χ^2 -test will be applied to assess whether there are significant differences between arms.
Differences of craving between the 2 arms assessed by QSU-G and VAS at week 4 and 12 relative to baseline will both be analysed by ANCOVA. Scores at week 4 or 12 will be the outcome variable, baseline BMI and treatment arm explanatory variables.
Change of haemoglobin A1c (HbA1c) concentration at week 12, 24, and 52, relative to baseline will be analysed by ANCOVA. HbA1c at week 12, 24, and 52 will be the outcome variable, HbA1c at baseline and treatment arm explanatory variables.
Time trends of means or percentages of all secondary endpoints will be plotted including standard deviations for means and 95%-CI for percentages.
Sensitivity analysis for primary and secondary outcomes will be done using the PP dataset.
For hypothesis testing an alpha error threshold of 5% will be chosen.

11.4 Interim Analysis

No interim analysis is planned.

11.5 Safety Analysis

The proportion of participants with an adverse or serious adverse event will be estimated by trial arm, and reported together with 95%-CIs. Safety analyses will be performed on the FAS.

11.6 Deviation(s) from the original statistical plan

If substantial deviations of the analysis as outlined in these sections are needed for whatever reason, the protocol will be amended.
All deviations of the analysis from the protocol or from the detailed analysis plan will be listed and justified in a separate section of the final statistical report and described in the study publication.

11.7 Handling of missing data and drop-outs

Careful trial planning and conducting will minimise the occurrence of missing data as far as possible.
Results will be summarised using all available data. Multiple attempts will be made to obtain missing data. If participants are not able to present at the study center, the CO measurement and urinary test (cotinine)

can be obtained at a home visit.
Considerable efforts will be made to minimize attrition due to technical or logistical reasons and minimize outcome measurements missing completely at random. We assume that the remaining missing measurements for the primary outcome are missing for reasons closely linked to the smoking status, and we assume that such patients are smokers. We will describe a detailed algorithm for handling of missing data in the statistical analysis plan, including sensitivity analyses addressing this issue.
This includes that for patients with missing values at-random, missing values at random will be imputed by linear interpolation of the preceding and subsequent measure, using a numeric time scale.
For patients with missing values with informative character, the method of inverse probability of censoring weights will be applied.
Criteria to decide whether missing values are at random or informative, will be described in the statistical analysis plan.

12. QUALITY ASSURANCE AND CONTROL

12.1 Data handling and record keeping / archiving

12.1.1 Case Report Forms

Study data will be recorded in Case Report Forms (CRF) in an encrypted fashion, i.e. by their individual study participant number.

12.1.2 Specification of source documents

- Written informed consent forms
- Demographic data of the patients
- Study number
- Visit dates
- Results of laboratory analysis

The source data will be stored in the department of Endocrinology, Diabetes and Metabolism, University Hospital Basel.

12.1.3 Record keeping / archiving

Records and documents pertaining the conduct of this study, including CRFs, consent forms, laboratory test results and clinical notes will be retained for 10 years.

12.2 Data management

12.2.1 Data Management System

The clinical data will be collected in an electronic data capture (EDC) system, named secuTrial®. The EDC system runs on a server maintained by the IT-department of the University Hospital Basel. The electronic CRF (eCRF) is implemented (set-up and adjusted) by the data management group at the Clinical Trial Unit (CTU) at the University Hospital Basel.

12.2.2 Data security, access and back-up

The EDC system is accessible via a standard browser on a WWW-connected device.
Password protection ensures that only authorized persons can enter the system to view, add or edit data according to their permissions. User administration and user training is performed by the CTU according to predefined processes. Back-up of secuTrial® study data is performed according to the processes of the IT-department of the University Hospital Basel.

12.2.3 Analysis and archiving

The EDC will be locked after all data has been monitored and all raised queries have been resolved. Data is exported and transferred to the investigator by the CTU according to internally defined processes. Data will be archived by the investigator.

12.2.4 Electronic and central Data validation

Data is entered into the eCRF and can be validated for completeness and discrepancies automatically. An audit trail system maintains a record of initial entries and changes (reasons for changes, time and date of changes, user identification of entry and changes).

12.3 Monitoring

The monitoring of the study is performed by the Clinical Trial Unit of the University Hospital Basel.

12.4 Audits and Inspections

Authorized representatives of the national or local authorities will be permitted to inspect or audit the facilities and records relevant to this study.

12.5 Confidentiality, Data Protection

All personal and medical information obtained for this study is confidential and disclosure to third parties other than those noted below is prohibited. Patients' data will be identified by study and subject ID number.
Confidentiality of the patients will be maintained by assigning patients a study number, keeping identifiers separate from the data and storing data in a locked file in the department of Endocrinology, Diabetes and Metabolism. The sponsor investigator, the principle investigator, co-investigators and the study nurses will have access to the encryption list.
Scientific reports generated from the study will not contain information that would identify the patients. After termination of the study records will be archived for ten years and then destroyed.
Upon the participant's permission, medical information may be given to his or her personal physician or other appropriate medical personnel responsible for her or his welfare.

12.6 Storage of biological material and related health data

Biological material, i.e. blood samples, will be handled according to good clinical practice and good laboratory practice. Samples will be collected in secure containers (Sarstedt Monovette®) and will be centrifuged to collect serum. Serum will be stored at – 80 C in a thermo-controlled ultra-deep freezer.
Blood samples are stored for eventual future research aims (Biobank Departement Endokrinologie). Records and documents pertaining the conduct of this study, including CRFs, consent forms, laboratory test results and clinical notes will be retained for at least 10 years.

13. PUBLICATION AND DISSEMINATION POLICY

The results of this study shall be published in a peer-reviewed journal and presented at scientific conferences. No use of professional writers is intended.
Criteria for authorship will be based on the rules of the International Committee of Medical Journal Editors and will include participation in the planning phase of the study, raising of funds, completion of assigned duties and promptness of response, number of patients recruited and completed into the trial and the level of involvement in data analysis and in the drafting of the manuscript. Individual contributions will be specified at the end of the text and upon submission of the manuscript, authors will declare that they participated in the trial and will have to revise forwarded versions and approve the final version within 5 working days. Potential conflicts of interest will be listed and specified.
The principal investigator decides whether abstracts are to be submitted to conferences, and how the results are distributed if more than one manuscript is to be drafted.

14. FUNDING AND SUPPORT

14.1 Funding

This is an investigator-initiated trial. The study is supported by funds of the University of Basel and the Clinic for Endocrinology, University Hospital Basel. We are currently applying for further funding to cover the study costs (e.g. Swiss National Science Foundation, propatient Foundation, Bangerter-Rhyner Foundation).

14.2 Other Support

The University Hospital Basel, will provide the location and infrastructure.
This is collaborative work of the Department of Endocrinology and the Medical Outpatient Department of the University Hospital of Basel, where recently (2012) a smoking cessation service was set up. The service is under the lead of a cardiologist and a general internist. Treatment consists of a combination of individual advice, behavioural support and pharmacotherapy according to current guidelines for smoking cessation. In average four new patients are seen each week (220 new patients per year), such that enough patients are available for recruitment.
By this interdisciplinary approach, which brings different disciplines of the University Hospital Basel together (endocrinology, internal medicine, cardiology, smoking cessation counsellors), the professional competence is assured.
Moreover the project is integrated in the Clinical Trail Unit (CTU) of the University of Basel, where necessary working space and infrastructure are available. Onsite-management activities by study-nurses, the set-up of quality assurance and control measures are taken over by the CTU.

15. INSURANCE

The Insurance will be provided by the Sponsor.

16. SIGNIFICANCE OF THE STUDY

Cigarette smoking associated morbidity and mortality is preventable. Efficient smoking cessation strategies are therefore of high interest. In clinical practice many patients struggle with obstacles associated with smoking cessation such as nicotine withdrawal syndrome and weight gain.
As outlined above GLP-1 analogues are thought to influence exactly these two main barriers and are therefore a very promising novel treatment option for patients, who wish to quit smoking.
So far, there are no published or on-going human studies addressing this question. This project is of high relevance as it opens new therapeutic possibilities in a field of health priority with immediate benefits for patients by potentially preventing the disastrous consequences of nicotine abuse and obesity.

17. REFERENCES

1. Jha P, Ramasundarahettige C, Landsman V, et al. 21st-century hazards of smoking and benefits of cessation in the United States. *N Engl J Med* 2013; **368**(4): 341-50.
2. Rigotti NA. Strategies to help a smoker who is struggling to quit. *JAMA* 2012; **308**(15): 1573-80.
3. Centers for Disease C, Prevention. Quitting smoking among adults--United States, 2001-2010. *MMWR Morb Mortal Wkly Rep* 2011; **60**(44): 1513-9.
4. Stead LF, Koilpillai P, Fanshawe TR, Lancaster T. Combined pharmacotherapy and behavioural interventions for smoking cessation. *Cochrane Database Syst Rev* 2016; **3**: CD008286.
5. Cahill K, Stevens S, Perera R, Lancaster T. Pharmacological interventions for smoking cessation: an overview and network meta-analysis. *Cochrane Database Syst Rev* 2013; (5): CD009329.
6. Henningfield JE, Miyasato K, Jasinski DR. Abuse liability and pharmacodynamic characteristics of intravenous and inhaled nicotine. *J Pharmacol Exp Ther* 1985; **234**(1): 1-12.

7. Siahpush M, Singh GK, Tibbits M, Pinard CA, Shaikh RA, Yaroch A. It is better to be a fat ex-smoker than a thin smoker: findings from the 1997-2004 National Health Interview Survey-National Death Index linkage study. *Tob Control* 2014; **23**(5): 395-402.
8. Chinn S, Jarvis D, Melotti R, et al. Smoking cessation, lung function, and weight gain: a follow-up study. *Lancet* 2005; **365**(9471): 1629-35; discussion 00-1.
9. Flegal KM, Troiano RP, Pamuk ER, Kuczmarski RJ, Campbell SM. The influence of smoking cessation on the prevalence of overweight in the United States. *N Engl J Med* 1995; **333**(18): 1165-70.
10. Aubin HJ, Farley A, Lycett D, Lahmek P, Aveyard P. Weight gain in smokers after quitting cigarettes: meta-analysis. *BMJ* 2012; **345**: e4439.
11. Parsons AC, Shraim M, Inglis J, Aveyard P, Hajek P. Interventions for preventing weight gain after smoking cessation. *Cochrane Database Syst Rev* 2009; (1): CD006219.
12. Willi C, Bodenmann P, Ghali WA, Faris PD, Cornuz J. Active smoking and the risk of type 2 diabetes: a systematic review and meta-analysis. *JAMA* 2007; **298**(22): 2654-64.
13. Duncan AE, Lessov-Schlaggar CN, Nelson EC, Pergadia ML, Madden PA, Heath AC. Body mass index and regular smoking in young adult women. *Addict Behav* 2010; **35**(11): 983-8.
14. Yeh HC, Duncan BB, Schmidt MI, Wang NY, Brancati FL. Smoking, smoking cessation, and risk for type 2 diabetes mellitus: a cohort study. *Ann Intern Med* 2010; **152**(1): 10-7.
15. Harris KK, Zopey M, Friedman TC. Metabolic effects of smoking cessation. *Nat Rev Endocrinol* 2016; **12**(5): 299-308.
16. Herrmann C, Goke R, Richter G, Fehmann HC, Arnold R, Goke B. Glucagon-like peptide-1 and glucose-dependent insulin-releasing polypeptide plasma levels in response to nutrients. *Digestion* 1995; **56**(2): 117-26.
17. Gutniak M, Orskov C, Holst JJ, Ahren B, Efendic S. Antidiabetogenic effect of glucagon-like peptide-1 (7-36)amide in normal subjects and patients with diabetes mellitus. *The New England journal of medicine* 1992; **326**(20): 1316-22.
18. Nauck MA, Heimesaat MM, Orskov C, Holst JJ, Ebert R, Creutzfeldt W. Preserved incretin activity of glucagon-like peptide 1 [7-36 amide] but not of synthetic human gastric inhibitory polypeptide in patients with type-2 diabetes mellitus. *The Journal of clinical investigation* 1993; **91**(1): 301-7.
19. Larsen PJ, Tang-Christensen M, Holst JJ, Orskov C. Distribution of glucagon-like peptide-1 and other preproglucagon-derived peptides in the rat hypothalamus and brainstem. *Neuroscience* 1997; **77**(1): 257-70.
20. Merchenthaler I, Lane M, Shughrue P. Distribution of pre-pro-glucagon and glucagon-like peptide-1 receptor messenger RNAs in the rat central nervous system. *J Comp Neurol* 1999; **403**(2): 261-80.

21. Mojsov S, Kopczynski MG, Habener JF. Both amidated and nonamidated forms of glucagon-like peptide I are synthesized in the rat intestine and the pancreas. *The Journal of biological chemistry* 1990; **265**(14): 8001-8.
22. Alvarez E, Roncero I, Chowen JA, Thorens B, Blazquez E. Expression of the glucagon-like peptide-1 receptor gene in rat brain. *J Neurochem* 1996; **66**(3): 920-7.
23. Egecioglu E, Engel JA, Jerlhag E. The glucagon-like peptide 1 analogue Exendin-4 attenuates the nicotine-induced locomotor stimulation, accumbal dopamine release, conditioned place preference as well as the expression of locomotor sensitization in mice. *PloS one* 2013; **8**(10): e77284.
24. Engel JA, Jerlhag E. Role of appetite-regulating peptides in the pathophysiology of addiction: implications for pharmacotherapy. *CNS Drugs* 2014; **28**(10): 875-86.
25. Morganstern I, Barson JR, Leibowitz SF. Regulation of drug and palatable food overconsumption by similar peptide systems. *Curr Drug Abuse Rev* 2011; **4**(3): 163-73.
26. Larsson A, Engel JA. Neurochemical and behavioral studies on ethanol and nicotine interactions. *Neurosci Biobehav Rev* 2004; **27**(8): 713-20.
27. Fachinformation des Arzneimittel-Kompendium der Schweiz®. 2015.
28. Egecioglu E, Steensland P, Fredriksson I, Feltmann K, Engel JA, Jerlhag E. The glucagon-like peptide 1 analogue Exendin-4 attenuates alcohol mediated behaviors in rodents. *Psychoneuroendocrinology* 2013; **38**(8): 1259-70.
29. Shirazi RH, Dickson SL, Skibicka KP. Gut peptide GLP-1 and its analogue, Exendin-4, decrease alcohol intake and reward. *PloS one* 2013; **8**(4): e61965.
30. Vallof D, Maccioni P, Colombo G, et al. The glucagon-like peptide 1 receptor agonist liraglutide attenuates the reinforcing properties of alcohol in rodents. *Addict Biol* 2016; **21**(2): 422-37.
31. Erreger K, Davis AR, Poe AM, Greig NH, Stanwood GD, Galli A. Exendin-4 decreases amphetamine-induced locomotor activity. *Physiol Behav* 2012; **106**(4): 574-8.
32. Graham DL, Erreger K, Galli A, Stanwood GD. GLP-1 analog attenuates cocaine reward. *Mol Psychiatry* 2013; **18**(9): 961-2.
33. Egecioglu E, Engel JA, Jerlhag E. The glucagon-like peptide 1 analogue, exendin-4, attenuates the rewarding properties of psychostimulant drugs in mice. *PloS one* 2013; **8**(7): e69010.
34. Davis JF, Schurdak JD, Magrisso IJ, et al. Gastric bypass surgery attenuates ethanol consumption in ethanol-preferring rats. *Biol Psychiatry* 2012; **72**(5): 354-60.
35. Suchankova P, Yan J, Schwandt ML, et al. The glucagon-like peptide-1 receptor as a potential treatment target in alcohol use disorder: evidence from human genetic association studies and a mouse model of alcohol dependence. *Transl Psychiatry* 2015; **5**: e583.

36. Dungan KM, Povedano ST, Forst T, et al. Once-weekly dulaglutide versus once-daily liraglutide in metformin-treated patients with type 2 diabetes (AWARD-6): a randomised, open-label, phase 3, non-inferiority trial. *Lancet* 2014; **384**(9951): 1349-57.
37. Umpierrez G, Tofe Povedano S, Perez Manghi F, Shurzinske L, Pechtner V. Efficacy and safety of dulaglutide monotherapy versus metformin in type 2 diabetes in a randomized controlled trial (AWARD-3). *Diabetes Care* 2014; **37**(8): 2168-76.
38. Wysham C, Blevins T, Arakaki R, et al. Efficacy and safety of dulaglutide added onto pioglitazone and metformin versus exenatide in type 2 diabetes in a randomized controlled trial (AWARD-1). *Diabetes care* 2014; **37**(8): 2159-67.
39. Nauck M, Weinstock RS, Umpierrez GE, Guerci B, Skrivanek Z, Milicevic Z. Efficacy and safety of dulaglutide versus sitagliptin after 52 weeks in type 2 diabetes in a randomized controlled trial (AWARD-5). *Diabetes Care* 2014; **37**(8): 2149-58.
40. Umpierrez GE, Blevins T, Rosenstock J, et al. The effects of LY2189265, a long-acting glucagon-like peptide-1 analogue, in a randomized, placebo-controlled, double-blind study of overweight/obese patients with type 2 diabetes: the EGO study. *Diabetes Obes Metab* 2011; **13**(5): 418-25.
41. Pi-Sunyer X, Astrup A, Fujioka K, et al. A Randomized, Controlled Trial of 3.0 mg of Liraglutide in Weight Management. *The New England journal of medicine* 2015; **373**(1): 11-22.
42. Dalsgaard NB, Bronden A, Vilsboll T, Knop FK. Cardiovascular safety and benefits of GLP-1 receptor agonists. *Expert Opin Drug Saf* 2017: 1-13.
43. Marso SP, Daniels GH, Brown-Frandsen K, et al. Liraglutide and Cardiovascular Outcomes in Type 2 Diabetes. *N Engl J Med* 2016; **375**(4): 311-22.
44. Marso SP, Bain SC, Consoli A, et al. Semaglutide and Cardiovascular Outcomes in Patients with Type 2 Diabetes. *N Engl J Med* 2016; **375**(19): 1834-44.
45. Anthenelli RM, Benowitz NL, West R, et al. Neuropsychiatric safety and efficacy of varenicline, bupropion, and nicotine patch in smokers with and without psychiatric disorders (EAGLES): a double-blind, randomised, placebo-controlled clinical trial. *Lancet* 2016; **387**(10037): 2507-20.
46. Chang PH, Chiang CH, Ho WC, Wu PZ, Tsai JS, Guo FR. Combination therapy of varenicline with nicotine replacement therapy is better than varenicline alone: a systematic review and meta-analysis of randomized controlled trials. *BMC Public Health* 2015; **15**: 689.
47. Tian J, Venn A, Otahal P, Gall S. The association between quitting smoking and weight gain: a systemic review and meta-analysis of prospective cohort studies. *Obes Rev* 2015; **16**(10): 883-901.
48. O'Hara P, Connett JE, Lee WW, Nides M, Murray R, Wise R. Early and late weight gain following smoking cessation in the Lung Health Study. *American journal of epidemiology* 1998; **148**(9): 821-30.
49. Tabakprävention A, Cornuz, Jacot-Sadowski, Humair. Ärztliche Rauchstoppberatung - Die Dokumentation für die Praxis

. *Basisdokumentation für Ärzte und Ärztinnen Frei von Tabak, 2015* 2015; **3. Auflage**
50. Müller V, Mucha RF, Ackermann K, Pauli P. Die Erfassung des Cravings bei Rauchern mit einer deutschen Version des "Questionnaire on Smoking Urges" (QSU-G). *Zeitschrift für Klinische Psychologie und Psychotherapie* Hogrefe Verlag Göttingen 2001; **30** (3): 164-71.

APPENDICES

Appendix 1: Informed Consent Form, V2, 03.05.2017

Appendix 2: QSU-G

Appendix 3: VAS smoking urge

Appendix 4: Substudy Energy

Appendix 5: Substudy fMRI
