# Supplementary material for: Glucagon-like peptide-1 analogues: a new way to quit smoking? (SKIP)—a structured summary of a study protocol for a randomized controlled study
Source: Trials. 2023 Apr 20;24:284. doi: 10.1186/s13063-023-07164-9 (PMC10120253; doi:10.1186/s13063-023-07164-9)
Supplement: Supplementary file 4 — Additional file 4. Clinical study protocol (SKIP fMRI). [file 13063_2023_7164_MOESM4_ESM.pdf]

**Appendix 5: Substudy fMRI**

## 1. Background

Smokers compared to non-smokers exhibit increased functional brain activations in the prefrontal and limbic regions when exposed to craving-related smoking cue videos [1]. In this clinical trial (SKIP-study) the GLP-1 analogue Dulaglutide (Trulicity®) is evaluated as a treatment for smoking cessation. Supposing that GLP-1 and analogues modulates nicotine induced reward system [2, 3] we hypothesize that treatment with Dulaglutide (Trulicity®) attenuates craving and therefore functional brain activation. The aim of the present substudy is to evaluate effects of Dulaglutide treatment on functional neuronal changes in smokers who want to quit smoking.

## 2. Recruitment and screening

All SKIP-participants meeting the following in- and exclusion criteria are invited to participate in this substudy (until target sample size is reached).

### 2.1. Inclusion criteria

- Age 18-60 years
- Written informed consent signed
- Daily smokers who are willing to quit **and** exhibit one of the following criteria:
  - ≥10 cigarettes per day **and**
  - At least moderate nicotine dependence defined by a Fagerstroem Score of ≥5 points
  - Treatment with Varenicline (Champix®)

### 2.2. Exclusion criteria

- Pregnancy (incl. wish to become pregnant within next 3 months) or breast feeding
- Pre-existing treatment with GLP-1 agonists
- History of pancreatitis
- Severe renal insufficiency (estimated glomerular filtration rate <30 ml/min/1,73 m2)
- Instable psychiatric conditions including Anorexia nervosa
- Medical conditions that affect brain function (e.g. stroke, epilepsy, space occupying lesions, multiple sclerosis, Parkinson’s disease, dementia, transient ischemic attack)
- Current use of medications that alter brain function
- Current illicit drug abuse including marijuana (alcohol ≤ 1 drink per day allowed)
- Claustrophobia
- Cardiac pacemaker, electronic device or ferromagnetic metal foreign bodies
- Known or suspected allergy to trial product or related products

## 3. Objective, outcome, assumptions

### 3.1 Objective

To investigate the influence of the GLP-1 analogue Dulaglutide (Trulicity®) versus placebo on functional neuronal changes in the prefrontal cortex and limbic region measured by different methods of fMRI in smokers who want to quit smoking.

### 3.2 Outcome

a) Behavioural outcomes include:

- Craving measured by a Visual Analogue Scale (VAS) [1].
- Working memory performance investigated by the N-back task scores.

b) Functional neuronal changes are assessed through the surrogate of blood oxygenated level dependent (BOLD) signal, an indirect measure of neural activity. Three echo-planar imaging (EPI) sequences will be performed to investigate neural substrates of nicotine craving, working memory and resting state functional networks.

c) Structural outcomes include structural plasticity of grey and white matter in regions parts of the reward pathway (i.e. anterior cingulate cortex, insula, striatum) and in subcortical regions. One T1 sequence and one DTI sequence will be performed to investigate changes in grey and white matter.
Changes in the reward pathway are also assessed in the cerebro-spinal fluid (CBF) from the Arterial Spin Labelling (ASL) sequence.

### 3.3 Assumptions:

a) At the behavioural level:

- Decrease in nicotine craving perceived by the participants and improvement in working memory performance under treatment with Dulaglutide (Trulicity®) compared to placebo.
- Decrease in nicotine craving perceived by the participants and improvement in working memory performance in quitters compared to persistent smokers.
- Decrease in nicotine craving perceived by the participants and improvement in working memory performance under Varenicline (Champix®) treatment (visit week 12 versus baseline) assessed in placebo treated participants.
- Positive correlation between working memory performance and the severity of nicotine dependence, nicotine exposure and craving.

b) At the functional neuronal level:

- Reduced BOLD signal in the reward and limbic pathway under treatment with Dulaglutide (Trulicity®) compared to placebo.

- Reduced BOLD signal in the reward and limbic pathway in quitters compared to persistent smokers.
- Reduced BOLD in the reward and limbic pathway under Varenicline (Champix®) treatment (visit week 12 versus baseline) assessed in placebo treated participants.
- Positive correlation between functional neuronal changes and the severity of nicotine dependence, nicotine exposure and craving.

c) At the structural neuronal level:

- Less grey matter and white matter atrophy under treatment with Dulaglutide (Trulicity®) compared to placebo.
- Grey matter and white matter plasticity in quitters compared to persistent smokers.
- Less grey matter and white matter atrophy under Varenicline (Champix®) treatment (visit week 12 versus baseline) assessed in placebo treated participants.
- Positive correlation between brain atrophy and the severity of nicotine dependence, nicotine exposure and and craving.

## 4. Assessment and Procedures

In addition to the regular study visits fMRI will be conducted at baseline (after inclusion and before injection of the first trial medication) and after 3 months of treatment (a time frame between visit 8 and 12 will be accepted).

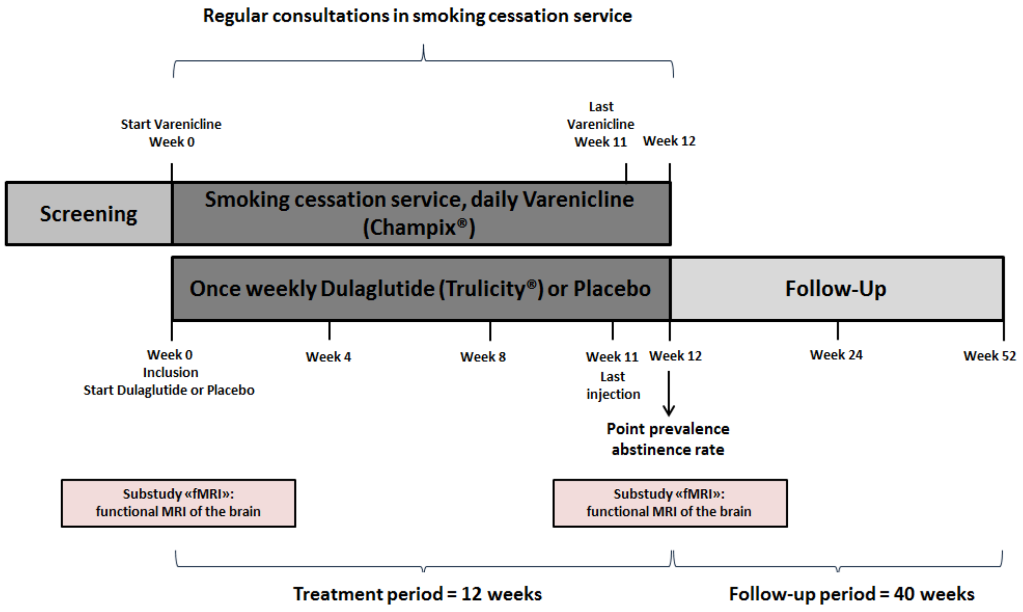


Before fMRI examination, smoking abstinence of at least 2 hours is required. Exposure to nicotine is assessed by end-expiratory exhaled carbon monoxide measurement, urinary cotinine (assessed at the regular visits 0 and 12), and former and actual nicotine consumption (pack years and average cigarettes per day during the last 3 days). Furthermore, severity of nicotine dependence is assessed at baseline by the Fagerstroem Score (at inclusion), craving by the German version of the “Questionnaire on Smoking

urges” (QSU-G) (assessed at the regular visits 0 and 12) and a Visual Analogue Scale (VAS) of smoking urge.

| Study procedure | Screening | Week 0 | Week 1 | Week 2 | Week 3 | Week 4 | Week 5 | Week 6 | Week 7 | Week 8 | Week 9 | Week 10 | Week 11 | Week 12 | Week 24 | Week 52 | End of Study Visit |
|---|---|---|---|---|---|---|---|---|---|---|---|---|---|---|---|---|---|
| **Study Visit** | | V0<br>V0 fMRI | | | | V4 | | | | V8 | | | | V12<br>V12 fMRI | V13 | V14 | EoSV fMRI |
| Inclusion / exclusion criteria | X | X | | | | | | | | | | | | | | | |
| Informed consent | X | X | | | | | | | | | | | | | | | |
| Inclusion / randomization | | X | | | | | | | | | | | | | | | |
| Medical history | | X | | | | | | | | | | | | | | | |
| Smoking status | | X | X | X | X | X | X | X | X | X | X | X | X | X | X | X | X |
| CO-measurement | | X | X | X | X | X | X | X | X | X | X | X | X | X | X | X | X |
| VAS smoking urge (0-10) | | X | X | X | X | X | X | X | X | X | X | X | X | X | X | X | X |
| QSU-G | | X | | | | X | | | | | | | | X | | | |
| Motivation | | X | | | | | | | | | | | | X | | X | X |
| Alcohol consumption/noxae | | X | | | | | | | | | | | | X | | X | X |
| Vital signs (AOBPM) | | X | | | | X | | | | X | | | | X | X | X | X |
| Weight, height, BMI | | X | | | | X | | | | X | | | | X | X | X | X |
| Laboratory testing | | X | | | | | | | | | | | | X | X | X | X |
| U-cotinine-measurement | | X | | | | | | | | | | | | X | X | X | X |
| Study drug | | X | X | X | X | X | X | X | X | X | X | X | X | | | | |
| Varenicline | | X | X | X | X | X | X | X | X | X | X | X | X | | | | |
| New Medication | | | X | X | X | X | X | X | X | X | X | X | X | X | X | X | X |
| Adverse events | | | X | X | X | X | X | X | X | X | X | X | X | X | X | X | X |
| **Substudy fMRI** | | | | | | | | | | | | | | | | | |
| fMRI | | X | | | | | | | | | | | | X | | | X |

### 4.1. fMRI procedure and acquisition

Participants undergo a block-design fMRI study performing two structural sequences: T1-weighting (T1) to investigate changes in grey matter and diffusor tensor imaging (DTI) to investigate changes in white matter.

Additionally, an Arterial Spin Labelling (ASL) sequence will be performed to assess changes in arterial perfusion.

Finally three functional sequences (EPI) will be performed to investigate changes in the BOLD signal: Resting State Sequence, a Nicotine Craving Task[1] to investigate nicotine craving, and an N-Back Task to investigate working memory-related brain activations.

#### 4.1.1. Nicotine craving task

The paradigm consists of an on-off block-design with two active conditions (smoking cue and control videos) and a neutral condition (cross displayed). The active condition uses video cues developed by Brody et al. [1, 4]. These cues are filmed from the first person point of view and are 45 seconds (s) in length. Each smoking video shows a potential craving situation, such as writing a letter and smoking a cigarette or standing outside of a nightclub smoking a cigarette. The control videos are matched for similar content except for the absence of smoking cues. After each video, a Visual Analogue Scale is presented for 2.5 s, and participants rate the degree of craving using an MR-compatible response box. The rating scale includes seven steps from no craving to high craving. After the rating, a rest period consists of the visual presentation of a fixation cross for 10 seconds. Each run includes five smoking and five control videos in a pseudo-randomized fashion and lasts 612 seconds. Each participant performs two runs. Before fMRI scanning, participants are instructed on the procedure and become familiar with the task by a training run outside of the MRI scanner.

#### 4.1.2. N-Back Task

A rapid, mixed trial, event-related fMRI design is used with jittered interstimulus intervals incorporating random event presentation to optimize statistical efficiency [5]. During the N-back task [6], all subjects see series of letters with an interstimulus interval of 2 s. Each stimulus is presented for 1 s. During a baseline (0-back) condition, subjects are required to press the button with the right hand when the letter „X” appears. During 1-back and 2-back conditions, participants are instructed to press the button if the currently presented letter is the same as that presented 1 (1-back condition) or 2 trials beforehand (2-back condition). The three conditions will be presented in ten alternating 30 s blocks (2 × 1-back, 3 × 2-back and 5 × 0-back) matched for the number of target letters per block (i.e., 2 or 3), in a pseudo-random order.

#### 4.1.3. Resting State Sequence

For the resting-state scan (5 minutes), subjects are instructed to lie in dimmed light with their eyes open, to think of nothing in particular, and not to fall asleep.

#### 4.1.4. T1, DTI and ASL sequences

During the structural scans, subjects are instructed to lie in scan without performing any particular tasks.

## 5. Statistical Methods

### 5.1 Sample Size

In total 60 patients who are willing to participate in the main and the substudy and meet the additional eligibility criteria. The decision to include 60 patients is based on feasibility criteria such as costs, availability of staff, infrastructure, and readiness of patients to participate. We suppose that 60 patients are sufficient to generate meaningful results that allow generation of hypotheses. Measurement of functional

brain activity by fMRI poses low risk to the patient. Therefore, the sample size is only limited by feasibility criteria.

### 5.2 Planned Analysis

The fMRI will be pre-processed using FSL (FSL Version 5.0.9; http://fsl.fmrib.ox.ac.uk) to make the data ready for statistical analyses. Details will be provided in a data management plan. There is an option to alter and add variables in an iterative process during statistical analysis.

The analysis set includes all patients that signed the informed consent of this substudy and performed at least one fMRI. Missing data will not be imputed but frequency and reasons for missingness described. No hypotheses will be tested. Explorative analyses are performed for hypothesis generation.

Summary statistics will be performed including reporting of frequencies of nominal and ordinal data and distribution parameters of numerical data.

All outcomes will be analysed by two group comparison at 12 weeks relative to baseline. Groups at 12-week visit are intervention or control arm, either quitters or persistent smokers. Analyses will be focussed on predefined assumptions described above. However, each possible combination of head-to-head comparisons will be made. Differences of outcomes between two groups will be analysed and frequentist p-values provided for each outcome of each comparison.

Further analyses include comparison of outcomes before and after Varenicline (Champix®) exposure in placebo treated participants and correlation of outcomes with the following variables:

- severity of nicotine dependence (Fagerstroem Score)
- nicotine exposure (pack years, actual number of cigarettes per day, urinary cotinine, end-expiratory exhaled carbon monoxide in ppm)
- craving (QSU-G, VAS)
- age
- sex

All alpha error levels will be reported. Despite alpha error inflation due to multiple testing, no alpha error correction will be performed. Interpretation of p-values will be of explorative nature and always reported as a whole.

Detailed description of variables, statistical models, and presentation of results will be provided in a statistical analysis plan.

Motivated by results, further explorative analyses that are not defined *a priori* in the statistical analysis plan might be carried out.

## REFERENCES


1. Brody, A.L., et al., *Neural substrates of resisting craving during cigarette cue exposure.* Biol Psychiatry, 2007. **62**(6): p. 642-51.
2. Engel, J.A. and E. Jerlhag, *Role of appetite-regulating peptides in the pathophysiology of addiction: implications for pharmacotherapy.* CNS Drugs, 2014. **28**(10): p. 875-86.
3. Zanchi, D., et al., *The impact of gut hormones on the neural circuit of appetite and satiety: A systematic review.* Neurosci Biobehav Rev, 2017. **80**: p. 457-475.
4. Zanchi, D., et al., *Cigarette smoking leads to persistent and dose-dependent alterations of brain activity and connectivity in anterior insula and anterior cingulate.* Addict Biol, 2015. **20**(6): p. 1033-41.
5. Ettinger, U., et al., *Functional magnetic resonance imaging of a parametric working memory task in schizophrenia: relationship with performance and effects of antipsychotic treatment.* Psychopharmacology (Berl), 2011. **216**(1): p. 17-27.
6. Broome, M.R., et al., *Neural correlates of executive function and working memory in the 'at-risk mental state'.* Br J Psychiatry, 2009. **194**(1): p. 25-33.
