# Supplementary material for: Glucagon-like peptide-1 analogues: a new way to quit smoking? (SKIP)—a structured summary of a study protocol for a randomized controlled study
Source: Trials. 2023 Apr 20;24:284. doi: 10.1186/s13063-023-07164-9 (PMC10120253; doi:10.1186/s13063-023-07164-9)
Supplement: Supplementary file 5 — Additional file 5. Clinical study protocol (SKIP Energy). [file 13063_2023_7164_MOESM5_ESM.pdf]

Appendix 4: Substudy “Energy”

1. Background

Total energy expenditure (TEE) is composed of resting energy expenditure (REE), the thermic effect of food and the physical activity expenditure [1]. REE, the energy which a fasting individual needs at rest in a thermoneutral environment, can be measured by indirect calorimetry. Beside physical activity and nutrition, several factors determine REE, e.g. age, gender and weight. Moreover, smoking and drugs may influence REE [1, 2].

Smokers show a 3-10% higher REE compared to nonsmokers [3-10]. This metabolic effect is thought to be mediated by nicotine cholinergic receptors in the brain and autonomic ganglia stimulating sympathetic nervous system and consequently release of catecholamines [8]. Smokers, who quit smoking, typically show an increase in body weight, which may be due to a decrease in resting energy expenditure by losing the nicotine effect [11].

Glucagon-like peptide 1 (GLP-1) analogues are well known for their weight lowering properties by suppression of appetite and energy intake. Moreover, an increase in REE (further promoting weight loss) has been discussed, but different studies conducted in heterogeneous settings showed controversial results [12-15].

In this clinical trial (SKIP-study) the GLP-1 analogue Dulaglutide (Trulicity®) is evaluated as a treatment for smoking cessation. The aim of the substudy “Energy” is to investigate the effect of Dulaglutide (Trulicity®) on REE and further parameters associated with energy metabolism (bodycomposition, haemodynamic parameters and catecholamine action) in a subset of patients recruited for the main trial.

2. Recruitment and screening

As pronounced under- and overweight influence energy expenditure, for this substudy a BMI range of 18-30 kg/m^2 is requested. All SKIP-participants meeting this additional criterion are invited to participate in this substudy (until target sample size is reached).

2.1 Inclusion criteria

- BMI 18-30 kg/m^2
- Age 18 to 75 years
- Daily smokers who are willing to quit <u>and</u> exhibit one of the following criteria:
    - ≥10 cigarettes per day **<u>or</u>**
    - At least moderate nicotine dependence defined by a Fagerstroem Score of ≥5 points **<u>or</u>**
    - Tobacco associated disease
- Treatment with varenicline (Champix®)
- Written informed consent signed

2.2 Exclusion criteria

- Pregnancy (incl. wish to become pregnant within next 3 months) or breast feeding
- Pre-existing treatment with GLP-1 agonists
- History of pancreatitis
- Severe renal insufficiency (estimated glomerular filtration rate <30 ml/min/1,73 m^2)
- Instable psychiatric conditions
- Anorexia nervosa
- Known or suspected allergy to trial product or related products

3. Objectives, outcomes and assumptions

3.1.1 Objective: REE

To investigate the influence of the GLP-1 analogue Dulaglutide (Trulicity®) versus placebo on REE in smokers who want to quit smoking.

3.1.2 Outcome

Kcal per 24 hours. It is assessed by indirect calorimetry measuring volume of oxygen uptake (VO_2) and expelled volume of carbon dioxide (VO_2) in ml/min and calculated by the Weir Equation REE = [3.9 * (VO_2) + 1.1 (VCO_2)] * 1.44. The respiratory quotient (RQ) is calculated by dividing VCO_2 by VO_2.

3.1.3 Assumptions

- Higher REE in Dulaglutide (Trulicity®) versus placebo treated participants at visit week 12.

- Lower REE in non-smokers versus persistent smokers, expecting however a less pronounced difference in participants treated with Dulaglutide (Trulicity®) compared to those receiving placebo.

- Similar REE during treatment with Varenicline (Champix®) comparing placebo treated participants at visit week 12 compared to baseline.

- Higher REE in male smokers, because of weight difference between genders.

3.2.1 Objective: body composition

To investigate the influence of the GLP-1 analogue Dulaglutide (Trulicity®) versus placebo on body composition in smokers who want to quit smoking.

3.2.2 Outcome

Body composition is assessed by bioelectrical impedance analysis. Measures are muscle and fat mass as a proportion (%) of total body weight.

3.2.3 Assumptions

- Change in body composition with lower fat but higher muscle mass in Dulaglutide (Trulicity®) versus placebo treated participants (comparing baseline to visit week 12).

- Due to post-cessational weight gain, change in body composition with higher fat but lower muscle mass in non-smokers versus persistent smokers, expecting however a less pronounced difference in participants treated with Dulaglutide (Trulicity®) compared to those receiving placebo.

3.3.1 Objective: haemodynamic parameters

To investigate the influence of the GLP-1 analogue Dulaglutide (Trulicity®) versus placebo on haemodynamic parameters (cardiac index, peripheral vascular resistance, volemia, inotropy and vasoactivity) in smokers who want to quit smoking.

3.3.2 Outcome

Blood pressure (mmHg), heart rate (beats per minute), cardiac index ($l/min/m^2$), and peripheral vascular resistance ($Pa*[s/m^3]$) assessed by non-invasive thoracic bioimpedance (HOTMAN®).

3.3.3 Assumptions

- Increased heart rate, lower peripheral vascular resistance and lower blood pressure in Dulaglutide (Trulicity®) versus placebo treated participants [16, 17].
- Lower peripheral vascular resistance and lower blood pressure in non-smokers versus persistent smokers.

3.4.1 Objective: sympathetic activity

To investigate the influence of the GLP-1 analogue Dulaglutide (Trulicity®) versus placebo on sympathetic activity in smokers who want to quit smoking.

3.4.2 Outcome

Plasma catecholamine (epinephrine and norepinephrine) and neuropeptide Y (NPY)* levels measured in pg/ml. NPY is a 36 aminoacid peptide well known to potentiate the action of catecholamine postsynaptically through the Y1 receptor and inhibit presynaptically the catecholamine secretion through the Y2 receptor [18].

3.4.3 Assumptions

The sympathetic nervous system may be stimulated both by nicotine and Dulaglutide (Trulicity®). Thus we expect:

- Higher plasma catecholamine and NPY levels in the Dulaglutide (Trulicity®) versus placebo treated

participants.

- Higher plasma catecholamine and NPY levels in smokers versus non-smokers.

4. Assessment

Additionally to the collected data from the main SKIP-study we will conduct indirect calorimetry, a bioelectrical impedance analysis, non-invasive thoracic bioimpedance measurement and measurement of plasma catecholamine and NPY levels at two time points (visit 0 “energy” and visit 12 “energy”). Furthermore self-perceived eating habits and physical activity will be captured. These additional assessments will be done at visit 0 and 12 <u>or</u> optionally at an additional visit as indirect calorimetry has to be done after an overnight fast. For visit 12 “energy” a time frame of 3 weeks (between visit 9 and 12) is accepted, while the main study outcome (point prevalence abstinence) will always be assessed at week 12 (+/- 7 days), see protocol paragraph 5.0). Haemodyncamic parameters by non-invasive thoracic bioimpedance measurement will additionally be assessed at week 24 and 52.

**Study procedure**	**Screening**	**Week 0**	**Week 1**	**Week 2**	**Week 3**	**Week 4**	**Week 5**	**Week 6**	**Week 7**	**Week 8**	**Week 9**	**Week 10**	**Week 11**	**Week 12**	**Week 24**	**Week 52**	**End of Study Visit**
**Study Visit**		**V0** **V0 energy**				**V4**				**V8**				**V12** **V12 energy**	**V13**	**V14**	**EoSV energy**
Inclusion / exclusion criteria	X	X															
Informed consent	X	X															
Inclusion / randomization		X															
Medical history		X															
Smoking status		X	X	X	X	X	X	X	X	X	X	X	X	X	X	X	X
CO-measurement		X	X	X	X	X	X	X	X	X	X	X	X	X	X	X	X
VAS smoking urge (0-10)		X	X	X	X	X	X	X	X	X	X	X	X	X	X	X	X
QSU-G		X				X								X			
Motivation		X												X		X	X
Alcohol consumption/noxae		X												X		X	X
Vital signs (AOBPM)		X				X				X				X	X	X	X
Weight, height, BMI		X				X				X				X	X	X	X
Laboratory testing		X												X	X	X	X
U-cotinine-measurement		X												X	X	X	X
Study drug		X	X	X	X	X	X	X	X	X	X	X	X				
Varenicline		X	X	X	X	X	X	X	X	X	X	X	X				
New Medication			X	X	X	X	X	X	X	X	X	X	X	X	X	X	X
Adverse events			X	X	X	X	X	X	X	X	X	X	X	X	X	X	X
Substudy Energy																	
Indirect calorimetry		X												X			X
HOTMAN®		X												X	X	X	X
BIA		X												X			X
Plasma catecholamines		X												X			X

4.1 Assessment of objective REE

Indirect calorimetry for measurement of REE will be assessed in the morning after an overnight fast of at least 10 hours in an air-conditioned room with a room temperature between 22-24° C with the patient wearing light clothing (i.e. underwear and t-shirt). To avoid activation of cold-induced thermogenesis and ensure a comfortably warm body surface temperature the patient will be covered by a blanket during the baseline measurement of REE. Energy expenditure will then be measured with the ventilated hood technique using a Cosmed Quark RMR (Cosmed, Rome, Italy) during 30 minutes. Considering pharmacokinetic of nicotine and for reasons of standardization participants who are smokers are requested to smoke the last cigarette immediately before indirect calorimetry takes place. Furthermore a maximum of 30 minutes of medium exercise during the preceding 24 h should be done. The unit of REE is kcal per 24 hours. It is assessed by volume of oxygen uptake (VO_2) and expelled volume of carbon dioxide (VO_2) in ml/min and calculated by the Weir Equation REE = [3.9 * (VO_2) + 1.1 (VCO_2)] * 1.44.

4.2 Assessment of further objective

4.2.1 Bioelectrical impedance analysis

Body composition, e.g. proportion of fat mass and muscle mass (%), will be assessed by a bioelectrical impedance analysis.

4.2.2 Non-invasive thoracic bioimpedance measurement

Haemodynamic parameters as blood pressure (mmHg), heart rate (beats per minute), cardiac index ($l/min/m^2$), peripheral vascular resistance ($Pa*[s/m^3]$, volemia, inotropy and vasoactivity will be non-invasively assessed in each patient at baseline (visit 0), at visit 12, 13 and 14 using the HOTMAN® System (Hemo Sapiens Medical Inc., Sedona, AZ, USA). For this measurement, the patient has to be in supine position. Electrodes will be positioned as shown in *Figure 1*. After 5 minutes of rest, 3 consecutive measurements spaced 2 min. apart will be recorded together with blood pressure measurements and the mean of the second and third measurement will be used for further calculations or comparisons.

Figure 1 – Placement of electrodes for thoracic bioimpedance

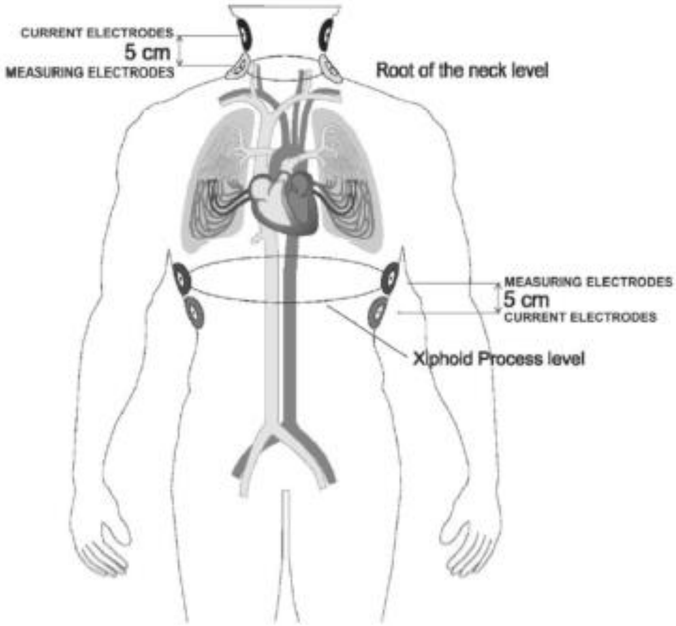

4.2.3 Measurement of plasma catecholamines and NPY

For later batch analysis of plasma catecholamines and NPY levels a 4.5ml heparin coated tube containing a cocktail of peptidase inhibitors on ice will be taken (NPY is a substrate for several proteases including DPP4 and kallikrein). To avoid stress stimuli participants have to lie calm for at least 20 minutes before blood drawing.

4.2.4 Eating habits and physical activity

At visit 12 participants will be asked whether their eating habits and physical activity has changed compared with the baseline visit.

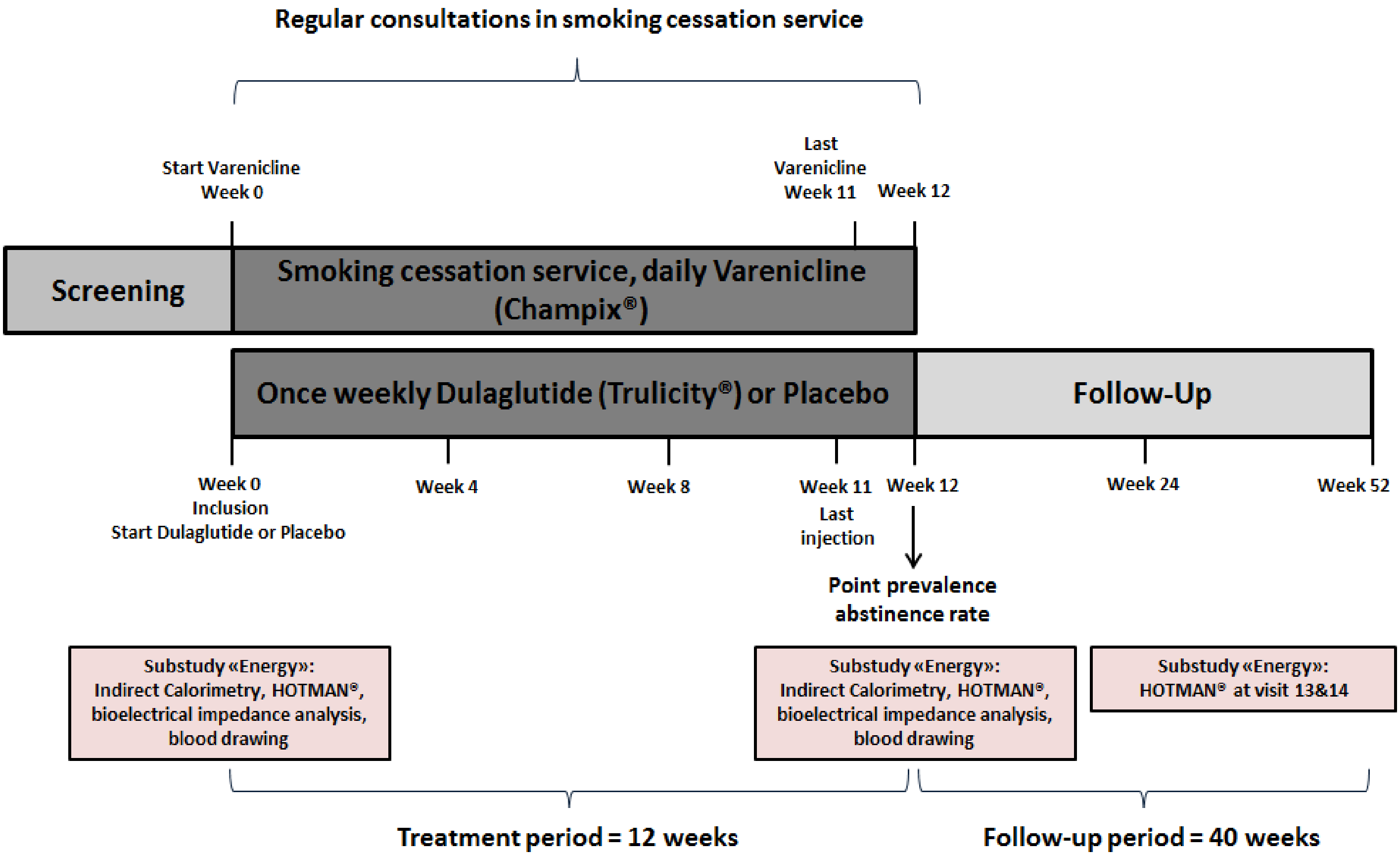

5. Procedures

Additionally to the regular planned visits we will carry out the following examinations:

Baseline visit (=visit 0 "energy"):

Indirect calorimetry, bioelectrical impedance analysis, non-invasive thoracic bioimpedance measurement, blood drawing (plasma catecholamines and NPY).

Visit 12 "energy":

Indirect calorimetry, bioelectrical impedance analysis, non-invasive thoracic bioimpedance measurement, blood drawing (plasma catecholamines and NPY).

Visit 13:

Non-invasive thoracic bioimpedance measurement

Visit 14 or end of study visit "energy":

Non-invasive thoracic bioimpedance measurement.

If the end of study visit takes place within the first 12 weeks, indirect calorimetry, bioelectrical impedance analysis and blood drawing (plasma catecholamins and NPY) will also be conducted.

6. Statistical Methods

Detailed methodology for summaries and statistical analyses of the data collected in this study will be documented in a statistical analysis plan.

6.1 Sample Size

In total 100 patients who are willing to participate in the main and the substudy and meet the additional inclusion criterion of a BMI between 18-30 kg/m^2 will be included.

The decision to include 100 patients is based on feasibility criteria such as costs, availability of staff, infrastructure, and readiness of patients to participate. We suppose that 100 patients are sufficient to generate meaningful results that allow generation of hypotheses. Assessment of the 4 outcomes REE, body composition, haemodynamic parameters, and sympathetic activity pose low risk to the patient. Therefore, the sample size is only limited by feasibility criteria.

6.2 Planned Analysis

The analysis set includes all patients that signed the informed consent and agreed to participate in the "Energy" substudy. Missing data will not be imputed but frequency and reason for missingness will be described. No hypotheses will be tested. Explorative analyses are performed solely for hypothesis generation.

If necessary, data will be transformed to approximate normal distribution. If distributions of outcomes allow, multivariable linear regression models will be fit. Outcome variables will be one of the following measures: REE (kcal/24h), RQ (rate), body and fat mass as a proportion of total body weight, blood pressure (mmHg), heart rate (beats per min), cardiac index (l/min/m^2), peripheral vascular resistance (Pa * [s/m^3]), and plasma catecholamine levels measured in pg/ml.

Explanatory variables will be treatment (intervention or control), smoker status (smoker or non-smoker), age, sex, BMI (kg/m^2), number of cigarettes consumed per day, extent of nicotine consumption (end-expiratory exhaled carbon monoxide in ppm, urinary cotinine), and muscle mass. For body composition, muscle mass will not be used as an explanatory variable. Model output will show whether treatment (intervention or control) and being smoker or non-smoker has an impact on outcomes.

For all objectives the following comparisons will be made:

- Intervention versus control arm at visit week 12 relative to baseline. Smoking status accounts as co-variable.
- Smokers versus non-smokers at visit week 12 relative to baseline. Study medication accounts as co-variable.
- Smokers at visit week 12 in the intervention arm relative to baseline.

- Smokers at visit week 12 in the control arm relative to baseline = effect of varenicline.
- Non-smokers at visit week 12 in the intervention arm relative to baseline.
- Non-smokers at visit week 12 in the control arm relative to baseline.
- Smokers versus non-smokers in the intervention arm at visit week 12.
- Smokers versus non-smokers in the control arm at visit week 12.
- Smokers in the intervention arm versus smokers in the control arm at visit week 12.
- Non-smokers in the intervention arm versus non-smokers in control arm at visit week 12.

Motivated by results, further explorative analyses that were not defined *a priori* might be carried out.

References

1. DeLany, J.P., *Measurement of energy expenditure.* Pediatr Blood Cancer, 2012. **58**(1): p. 129-34.
2. Psota, T. and K.Y. Chen, *Measuring energy expenditure in clinical populations: rewards and challenges.* Eur J Clin Nutr, 2013. **67**(5): p. 436-42.
3. Hofstetter, A., et al., *Increased 24-hour energy expenditure in cigarette smokers.* N Engl J Med, 1986. **314**(2): p. 79-82.
4. Perkins, K.A., et al., *Acute effects of nicotine on resting metabolic rate in cigarette smokers.* Am J Clin Nutr, 1989. **50**(3): p. 545-50.
5. Perkins, K.A., et al., *The effect of nicotine on energy expenditure during light physical activity.* N Engl J Med, 1989. **320**(14): p. 898-903.
6. Perkins, K.A. and J.E. Sexton, *Influence of aerobic fitness, activity level, and smoking history on the acute thermic effect of nicotine.* Physiol Behav, 1995. **57**(6): p. 1097-102.
7. Perkins, K.A., J.E. Sexton, and A. DiMarco, *Acute thermogenic effects of nicotine and alcohol in healthy male and female smokers.* Physiol Behav, 1996. **60**(1): p. 305-9.
8. Walker, J.F., et al., *The effect of smoking on energy expenditure and plasma catecholamine and nicotine levels during light physical activity.* Nicotine Tob Res, 1999. **1**(4): p. 365-70.
9. Gonseth, S., et al., *Association between smoking and total energy expenditure in a multi-country study.* Nutr Metab (Lond), 2014. **11**(1): p. 48.
10. Blauw, L.L., et al., *Smoking is associated with increased resting energy expenditure in the general population: The NEO study.* Metabolism, 2015. **64**(11): p. 1548-55.
11. Moffatt, R.J. and S.G. Owens, *Cessation from cigarette smoking: changes in body weight, body composition, resting metabolism, and energy consumption.* Metabolism, 1991. **40**(5): p. 465-70.
12. Pannacciulli, N., et al., *Higher fasting plasma concentrations of glucagon-like peptide 1 are associated with higher resting energy expenditure and fat oxidation rates in humans.* Am J Clin Nutr, 2006. **84**(3): p. 556-60.
13. Shalev, A., J.J. Holst, and U. Keller, *Effects of glucagon-like peptide 1 (7-36 amide) on whole-body protein metabolism in healthy man.* Eur J Clin Invest, 1997. **27**(1): p. 10-6.
14. van Can, J., et al., *Effects of the once-daily GLP-1 analog liraglutide on gastric emptying, glycemic parameters, appetite and energy metabolism in obese, non-diabetic adults.* Int J Obes (Lond), 2014. **38**(6): p. 784-93.
15. Horowitz, M., et al., *Effect of the once-daily human GLP-1 analogue liraglutide on appetite, energy

intake, energy expenditure and gastric emptying in type 2 diabetes. Diabetes Res Clin Pract, 2012. **97**(2): p. 258-66.

16. Kumarathurai, P., et al., *Effects of Liraglutide on Heart Rate and Heart Rate Variability: A Randomized, Double-Blind, Placebo-Controlled Crossover Study.* Diabetes Care, 2017. **40**(1): p. 117-124.

17. Andrews, C.N., et al., *Effects of glucagon-like peptide-1 and sympathetic stimulation on gastric accommodation in humans.* Neurogastroenterol Motil, 2007. **19**(9): p. 716-23.

18. Reichmann, F. and P. Holzer, *Neuropeptide Y: A stressful review.* Neuropeptides, 2016. **55:** p.99-109.
