# Supplementary material for: Glucagon-like peptide-1 analogues: a new way to quit smoking? (SKIP)—a structured summary of a study protocol for a randomized controlled study
Source: Trials. 2023 Apr 20;24:284. doi: 10.1186/s13063-023-07164-9 (PMC10120253; doi:10.1186/s13063-023-07164-9)
Supplement: Supplementary file 6 — Additional file 6. Informed consent form (energy), informed consent form (fMRI), informed consent form (main study). [file 13063_2023_7164_MOESM6_ESM.zip › Appendix 06 Informed Consent Form (fMRI)R1.pdf]

The SKIP Study - Smoking cessation facilitated by glucagon-like peptide-1 (GLP-1) analogues - a randomized, double-blind, placebo-controlled trial Substudy fMRI

Die SKIP-Studie - Rauchstopp unter GLP-1 Analoga - eine randomisierte, doppelblinde, Placebo-kontrollierte Studie Substudie fMRI

Diese Studie ist organisiert durch: Prof. Dr. med. Mirjam Christ-Crain

Sehr geehrte Dame, sehr geehrter Herr
Wir möchten Sie anfragen, ob Sie an einer klinischen Studie teilnehmen wollen. Im Folgenden wird Ihnen dieses Studienvorhaben dargestellt: zunächst in einer kurzen Zusammenfassung, damit Sie wissen, um was es geht, anschliessend in einer detaillierten Beschreibung.

Zusammenfassung

1	**Ziel der Studie**<br>Die Studie untersucht die Auswirkungen des Medikamentes Dulaglutid (Trulicity®) auf die Raucherentwöhnung und die damit oft verbundene Gewichtszunahme.
2	**Auswahl der Studienteilnehmenden**<br>Wir lassen Ihnen diese Informationsschrift zukommen, da sie aktuell rauchen und damit aufhören wollen.
3	**Allgemeine Informationen zur Studie**<br>Diese Studie richtet sich an Personen, welche gerne mit dem Rauchen aufhören würden. Dulaglutid (Trulicity®), ein Medikament, welches die Wirkung des körpereigenen Darmhormons (GLP-1) imitiert, wird gegen Zuckerkrankheit (Diabetes mellitus) und Übergewicht eingesetzt. Neuste Resultate aus Tierstudien weisen darauf hin, dass GLP-1 bei verschiedensten Suchterkrankungen (z.B. Nikotin, Alkohol, Kokain) eine Rolle spielt. Des Weiteren wollen wir mit dieser Substudie untersuchen, inwiefern Dulaglutid (Trulicity®), das Rauchen und auch ein möglicher Rauchstopp die Strukturen und die Aktivität im Gehirn beeinflussen.<br>Mit dieser Studie möchten wir erforschen, ob Dulaglutid (Trulicity®) Entzugserscheinungen nach einem Rauchstopp lindert und ob die oft damit verbundene Gewichtszunahme vermindert werden kann. Es handelt sich um eine doppelblinde, Placebo-kontrollierte Studie, was bedeutet, dass weder wir vom Studienteam noch Sie als Proband wissen, ob Sie das Studienmedikament (Dulaglutid [Trulicity®]) oder ein Scheinmedikament (Placebo) erhalten. Wer das Medikament und wer das Placebopräparat erhält, wird nach dem Zufallsprinzip entschieden (=randomisiert) und kann nicht beeinflusst werden.<br>Insgesamt werden wir 256 Personen in die Studie einschliessen, die Studiendauer ist voraussichtlich 3 Jahre. In der Substudie fMRI (functional magnetic resonance imaging) werden wir zusätzlich bei 60 der 256 Studienteilnehmern eine Bildgebung (fMRI des Schädels) durchführen.

4	**Studienablauf**<br>Parallel zur Studie werden Sie in der Rauchstopp-Sprechstunde des Universitätsspitals Basel behandelt und dort während 12 Wochen die Standardtherapie für eine Raucherentwöhnung erhalten, welche aktuell einer Verhaltenstherapie und der Einnahme von Vareniclin [Champix®] entspricht. Zusätzlich erhalten Sie im Rahmen der Studie das Studienmedikament (Dulaglutid [Trulicity®]) oder ein Scheinmedikament (Placebo) einmal wöchentlich durch uns unter die Haut (subkutan) gespritzt. Während dieser Zeit werden wir Sie regelmässig in Studienvisiten sehen, wobei wir Daten erheben (bspw. Gewicht, Blutdruck, Fragebögen), den Kohlenmonoxidgehalt in Ihrer Ausatemluft messen und gelegentlich Blut- und Urinproben abnehmen. Anschliessend erfolgen nach 6 und nach 12 Monaten Nachkontrollen bei uns.<br>Bei der Substudie fMRI werden wir zu Beginn der Studie und nach 12 Wochen ein fMRI des Schädels durchführen, wo wir die Hirnstruktur und die Hirnaktivität unter dem Einfluss von Dulaglutid (Trulicity®), dem Rauchen und einem möglichen Rauchstopp untersuchen werden. Um die Effekte des Nikotinentzugs besser darstellen zu können, dürfen Sie zwei Stunden vor dem fMRI nicht rauchen, zudem werden wir die Kohlenmonoxid-Konzentration in Ihrer Ausatemluft messen. Die erste sowie Visite 12 werden aufgrund der fMRI Untersuchung ca. zwei Stunden dauern, wobei es aus organisatorischen Gründen sein kann, dass die erste Visite aufgesplittet wird und Sie für das fMRI einen separaten Termin erhalten werden. Die restlichen Visiten dauern jeweils ca. 30 Minuten.
5	**Nutzen**<br>Durch die Studienteilnahme ermöglichen Sie es uns, wichtige Informationen über den Einfluss von GLP-1-Analoga auf die Raucherentwöhnung zu gewinnen. Im Idealfall profitieren Sie von einer Behandlung, welche Ihnen den Rauchstopp erleichtert und einer Gewichtszunahme entgegen wirkt. Andererseits ist es auch möglich, dass Sie keinen persönlichen Nutzen daraus ziehen können. Sollte sich unsere Studienfrage als richtig erweisen, könnte Dulaglutid (Trulicity®) in Zukunft als neue Therapie zur Raucherentwöhnung genutzt werden.<br>Falls sich unsere Studienfrage als richtig erweist und eine Gewichtszunahme während des Rauchstopps verhindert werden kann, ist es möglich, dass auch Sie davon profitieren. Die Resultate können wichtig sein für andere, die nach Ihnen mit dem Rauchen aufhören wollen.
6	**Rechte**<br>Sie nehmen freiwillig teil. Wenn Sie nicht mitmachen oder später Ihre Teilnahme zurückziehen wollen, müssen Sie dies nicht begründen. Ihre medizinische Behandlung/Betreuung ist unabhängig von Ihrem Entscheid gewährleistet. Sie dürfen jederzeit Fragen zur Studienteilnahme stellen. Wenden Sie sich dazu bitte an die Person, die am Ende dieser Information genannt ist.

7	**Pflichten**<br>Wenn Sie an dieser Studie teilnehmen, bitten wir Sie, bestimmte Anforderungen einzuhalten. Dies ist notwendig für Ihre Sicherheit und Gesundheit.<br>Als Studien-TeilnehmerIn sind Sie verpflichtet:<br>- sich an den Studienplan zu halten<br>- den Studienarzt/Studienärztin über festgestellte unerwünschte Wirkungen zu informieren<br>- den Studienarzt/Studienärztin über neue Symptome und Beschwerden zu informieren<br>- den Studienarzt/Studienärztin über die gleichzeitige Behandlung und Therapien bei einem anderen Arzt und über Einnahme von Medikamenten zu informieren, auch solche, die Sie selbst/ohne Rezept gekauft haben. Insbesondere sollten während der gesamten Studiendauer keine Nikotinersatzpräparate (z.B. Pflaster, Kaugummi) angewendet werden.
8	**Risiken**<br>Als unerwünschte Ereignisse von Dulaglutid (Trulicity®) können Magendarmbeschwerden wie Übelkeit (bis ca. 20% der behandelten Personen) sowie seltener Erbrechen, Durchfall, Verstopfung und Bauchschmerzen auftreten. Die Beschwerden treten insbesondere zu Beginn der Behandlung auf und lassen in der Regel innerhalb weniger Tage unter fortgesetzter Behandlung nach.<br>Während der gesamten Studiendauer wird rund 150ml Blut entnommen, was weniger ist als bei einer normalen Blutspende (450ml).<br>Magnetresonanztomographische Untersuchungen (MRT) dienen der bildlichen Darstellung des Körpers mit Hilfe von Magnetfeldern (d.h. ohne Röntgenstrahlung). Anhaltende Nebenwirkungen infolge einer Magnetresonanztomographie sind nicht bekannt. Personen, die schwanger sind oder stillen, die Metallteile oder medizinische Implantate (z.B. Pumpen oder Herzschrittmacher) tragen, dürfen nicht in MR-Geräten untersucht werden. Teilen Sie dies bitte in jedem Fall dem behandelnden Arzt mit und füllen Sie das Ihnen ausgehändigte Formular wahrheitsgetreu aus. Bei Unklarheiten fragen Sie bitte das Studienpersonal.
9	**Andere Behandlungsmöglichkeiten**<br>Es besteht die Möglichkeit, nur die Standardtherapie (Verhaltenstherapie und Einnahme von Vareniclin [Champix®]) im Rahmen der Rauchstopp-Sprechstunde anzuwenden. Auch können Sie selbstständig versuchen, mit dem Rauchen aufzuhören.
10	**Ergebnisse aus der Studie**<br>Bei Studienergebnissen während der Studie werden Sie informiert, wenn diese Ergebnisse für Sie gesundheitlich wichtig sind. Falls Sie dies nicht möchten, informieren Sie bitte Ihren Prüfarzt. Dies gilt ebenso für mögliche Zufallsbefunde, welche im MRI des Schädels entdeckt werden könnten.
11	**Vertraulichkeit von Daten und Proben**<br>Wir halten alle gesetzlichen Regeln des Datenschutzes ein. Wir verwenden Ihre Daten nur im Rahmen der Studie. Alle Beteiligten unterliegen der Schweigepflicht. Ihre Blut- und Urinproben werden verschlüsselt verwendet und geschützt.
12	**Rücktritt**<br>Sie können jederzeit ohne Begründung von der Studie zurücktreten und nicht mehr teilnehmen. Die bis dahin erhobenen Daten und Proben werden noch ausgewertet.

13	**Entschädigung**<br>Das Studienmedikament wird ihnen gratis zur Verfügung gestellt Sie erhalten bei abgeschlossener Studie eine Aufwandsentschädigung. Die regulären Konsultationen im Rahmen der Rauchstopp-Sprechstunde werden von ihrer Krankenkasse übernommen. Insgesamt entstehen durch die Studie weder für sie noch für ihre Krankenkasse zusätzliche Kosten. Für die fMRI Untersuchung erhalten Sie als zeitliche Aufwandsentschädigung (jeweils eine Stunde) sFr. 50.- pro Termin. Die Gesamtsumme von sFr. 100.- wird Ihnen nach dem zweiten fMRI-Termin ausgehändigt.
14	**Haftung**<br>Das Universitätsspital Basel ersetzt Ihnen Schäden, die Sie gegebenenfalls im Rahmen des klinischen Versuchs erleiden. Zu diesem Zweck hat das Universitätsspital Basel zu Ihren Gunsten eine Versicherung bei Helvetia Versicherungen abgeschlossen.
15	**Finanzierung**<br>Die Studie wird hauptsächlich durch das Universitätsspital Basel und die Universität Basel gesponsert.
16	**Kontaktperson**<br>Dr. med. B. Winzeler, +41 61 556 50 75<br>Prof. Dr. med. M. Christ-Crain, +41 61 328 70 80<br>Notfall-Nummer (24 Stunden): Zentrale Universitätsspital: 061 265 25 25, Dr. med. B. Winzeler oder Dienstarzt Endokrinologie/Diabetologie verlangen<br>Endokrinologie, Diabetologie und Metabolismus, Universitätsspital Basel<br>Petersgraben 4, CH-4031 Basel

Nebst dieser Kurzfassung finden Sie auf den nachfolgenden Seiten umfassende Zusatzinformationen. Diese sind integrierter Bestandteil der Information. Mit der Unterzeichnung der Einwilligungserklärung akzeptieren Sie das vollständige Dokument.

Detaillierte Information

1. Ziel der Studie

Mit dieser Studie soll geprüft werden, ob das Medikament Dulaglutid (Trulicity®) im Vergleich zu Placebo hilft, mit dem Rauchen aufzuhören. Rauchen ist mit einer deutlich erhöhten Krankheitsrate und Sterblichkeit vergesellschaftet. Obwohl viele Raucher gerne mit dieser Sucht brechen möchten, scheitern sie im Alltag an den Hindernissen, welche ein Rauchstopp mit sich bringt. Hier sind in erster Linie Nikotin-Entzugserscheinungen und Gewichtszunahme zu nennen. Ziel dieser Studie ist es, das Medikament Dulaglutid (Trulicity®), welches genau auf diese zwei Barrieren abzielt, als neue Behandlung zur Raucherentwöhnung zu untersuchen. Dulaglutid (Trulicity®) imitiert die Wirkung des körpereigenen Darmhormons (GLP-1) und wird dank seiner gewichtsreduzierenden Wirkung erfolgreich zur Behandlung von Diabetes mellitus (Zuckerkrankheit) und Übergewicht eingesetzt. Neuste Resultate aus Tierstudien weisen darauf hin, dass GLP-1 bei verschiedensten Suchterkrankungen (z.B. Nikotin, Alkohol, Kokain) eine Rolle spielt. Wir gehen deshalb davon aus, dass Dulaglutid (Trulicity®) die Entzugserscheinungen nach Rauchstopp lindert und gleichzeitig einen gewichtsreduzierenden Effekt hat. Zusätzlich wollen wir mit der Substudie fMRI untersuchen, wie sich Dulaglutid (Trulicity®), das Rauchen und auch ein Rauchstopp auf die Hirnstruktur und die Hirnaktivität auswirken.

2. Auswahl der Studienteilnehmenden

An der Studie teilnehmen können volljährige Personen, die täglich rauchen und damit aufhören möchten. Dabei müssen Sie entweder pro Tag mindestens 10 Zigaretten rauchen, im Fagerstroem Score, welcher die Nikotinabhängigkeit erfasst, mindestens 5 Punkte erreichen oder an einer mit dem Rauchen assoziierten Erkrankung leiden. Zudem sollten Sie bereit sein, sich in der Rauchstopp-Sprechstunde des Universitätsspitals Basel behandeln zu lassen und Vareniclin (Champix®) einzunehmen.
Nicht teilnehmen können Personen, welche eine schwere Niereninsuffizienz haben, bereits einmal eine Bauchspeicheldrüsenentzündung (Pankreatitis) hatten oder falls eine aktive psychiatrische Erkrankung vorliegt. Ebenso ausgeschlossen sind schwangere oder stillende Frauen und solche, bei welchen in den nächsten drei Monaten ein Schwangerschaftswunsch besteht.

3. Allgemeine Informationen zur Studie

Es handelt sich um eine nationale Studie, welche im Universitätsspital Basel durchgeführt wird und voraussichtlich 3 Jahre dauern wird. Insgesamt werden 256 Personen in die Studie eingeschlossen, wovon 60 gleichzeitig an der Substudie fMRI teilnehmen werden. Parallel zur Studie erfolgt eine Behandlung in der der Rauchstopp-Sprechstunde des Universitätsspitals Basel, welche nebst einer individuellen Beratung eine medikamentöse Therapie mit Vareniclin (Champix®) (Dosis 2mg/Tag) erfasst. Dies entspricht aktuell der besten Rauchstopp-Therapie (=Goldstandard).
Um die Wirkung von Dulaglutid (Trulicity®) zu untersuchen, erhält jeder Proband während 12 Wochen entweder das echte Medikament (Dulaglutid [Trulicity®], Dosis 0.75 mg [erste Gabe] 1.5mg in 0.5ml) oder ein Placebopräparat. Das Placebopräparat enthält keinen Wirkstoff und ist somit ein „Scheinmedikament". Wer das echte Medikament und wer das Placebopräparat erhält, wird nach dem Zufallsprinzip entschieden und kann nicht beeinflusst werden. Beide Präparate werden einmal wöchentlich für insgesamt 12 Wochen durch uns unter die Haut (subkutan) gespritzt. Während dieser Zeit finden regelmässige Studienvisiten statt. Danach erfolgen nach 6 und nach 12 Monaten Nachkontrollen mit Blut- und Urinentnahmen sowie Messung des Kohlenmonoxidwertes in der Ausatemluft. Die gesamte Studiendauer beträgt somit für den einzelnen Teilnehmer 12 Wochen mit zwei Nachkontrollen nach 6
und 12 Monaten. Dulaglutid (Trulicity®) wird in der Schweiz bislang zur Behandlung von Diabetes mellitus (Zuckerkrankheit) und Übergewicht eingesetzt.
In der Substudie fMRI werden wir mittels fMRI folgende Dinge untersuchen:
- Die Struktur des Gehirns.
- Die Hirnaktivität, indem die Stärke der Durchblutung in unterschiedlichen Hirnarealen gemessen wird.
- Die Hirnaktivität unter der Stimulation mit Videos, wobei Ihnen Situationen aus dem alltäglichen Leben und Situationen mit Bezug zum Rauchen gezeigt werden. Dabei müssen Sie Ihr aktuelles Verlangen nach einer Zigarette angeben.
- Des Weiteren wird ein kurzer Konzentrationstest durchgeführt.

All diese Werte erheben wir sowohl zu Beginn wie auch nach 12 Wochen. Diese Studie wird nach geltenden schweizerischen Gesetzen und nach international anerkannten Grundsätzen durchgeführt. Die zuständige Kantonale Ethikkommission und Swissmedic haben die Studie geprüft und bewilligt. Eine Beschreibung finden Sie auch auf der Internetseite des Bundesamtes für Gesundheit: www.kofam.ch

4. Studienablauf

Bild 1: Zeitlicher Studienablauf

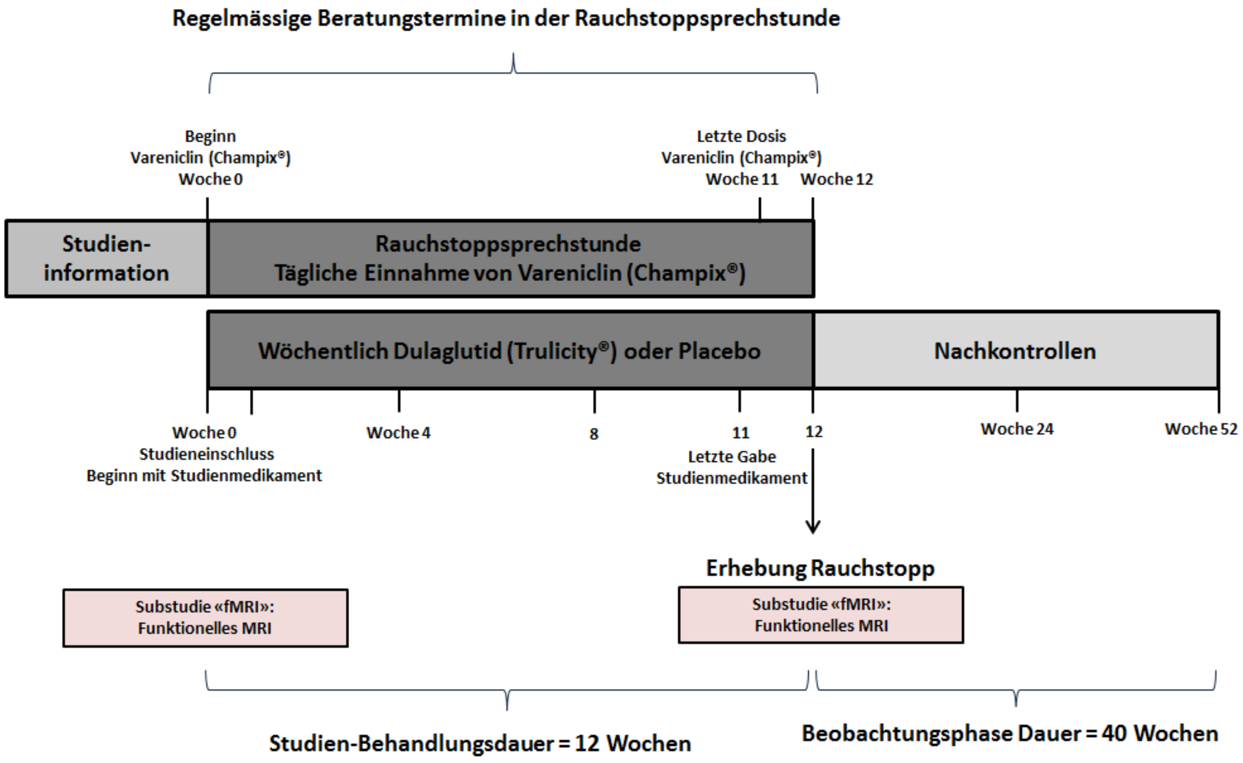

Im Rahmen ihrer ersten Konsultation in der Rauchstopp-Sprechstunde wird geklärt, ob Sie an der Studie teilnehmen können. Anschliessend erhalten Sie einen Termin, an dem Sie definitiv in die Studie eingeschlossen werden. Es erfolgt eine kurze körperliche Untersuchung (wir messen den Blutdruck, den Puls und ihr Gewicht), eine Blutentnahme und bei Frauen im gebärfähigen Alter ein Schwangerschaftstest. Anschliessend wird das Studienmedikament zum ersten Mal verabreicht. Dabei wird per Zufallsprinzip entschieden (=randomisiert), ob Sie Dulaglutid (Trulicity®) oder ein Placebopräparat erhalten. Das Studienmedikament wird ihnen für insgesamt 12 Wochen wöchentlich von uns unter die Haut (=subkutan) gespritzt wird. Das Studienmedikament wird bei der ersten Spritze in halber Dosis gespritzt, um eine bessere Verträglichkeit zu gewährleisten. Da Sie an der Substudie fMRI teilnehmen, werden wir vor der erstmaligen Verabreichung des Studienmedikaments ein fMRI des Schädels durchführen. Aus organisatorischen Gründen kann dafür ein separater Termin nötig sein. Nach 12 Wochen erfolgt nochmals ein fMRI, welches idealerweise gleichzeitig wie die letzte Studienvisite stattfindet.
Zusätzlich zu Ihren Kontrollterminen in der Rauchstopp-Sprechstunde (gemäss individuellem Bedarf, z. B. in den Wochen 1, 2, 4, 8, 12) werden wir Sie drei Mal für eine längere Studienvisite sehen (in den Wochen 4, 8, 12) und uns nach ihrem Wohlergehen erkundigen, Sie über ihren Raucherstatus befragen sowie folgende Parameter erheben: Ihren Blutdruck, Ihr Puls und Ihr Gewicht. Den Kohlenmonoxidgehalt in ihrer Ausatemluft werden wir wöchentlich nach der Injektion der Studienmedikation und nach 6 und 12 Monaten messen. Der Kohlenmonoxidgehalt gibt uns einen Hinweis, ob und wie viel Sie geraucht haben. Aus dem gleichen Grund nehmen wir in den Wochen 0 und 12 sowie nach 6 und 12 Monaten Urinproben von ihnen. Darin messen wir den Cotinin-Wert, welcher uns Rückschlüsse darüber gibt, ob die letzten 5 Tage geraucht wurde.

Zusätzlich werden Sie einen kurzen Fragebogen ausfüllen, welcher die Stärke ihrer Entzugserscheinungen erfasst (Visite 0, 4, 12) und wir werden Sie bei jeder Visite fragen, wie gross ihr aktuelles Verlangen nach einer Zigarette ist. In der zwölften Woche findet nach Abschluss der Studienmedikation eine vorerst letzte etwas längere Visite mit einer Blutentnahme statt. Falls Sie noch Raucher sind, erfragen wir Ihre Motivation, mit dem Rauchen aufzuhören.
Anschliessend werden wir Sie nach 6 und nach 12 Monaten nochmals für einen Termin kontaktieren, um Sie nach ihren Rauchgewohnheiten und ihrem Befinden zu befragen sowie Blutdruck, Puls, Gewicht und Kohlenmonoxidgehalt in der Ausatemluft zu messen. Auch erfolgt nochmals die Abnahme einer Blut- und Urinprobe. Insgesamt nehmen wir während der Studie viermal Blut ab (Woche 0, 12, 24, 52), was insgesamt einer Blutmenge von 150ml entspricht. Nach 12 Monaten ist die Studie dann beendet.
Zeitlich ist bei der ersten Visite sowie bei Visite 12 mit einer Dauer von 2 Stunden zu rechnen, bei den restlichen Visiten mit jeweils 30 Minuten.

Ihre Teilnahme kann durch den Prüfer/Ihre Prüferin jederzeit frühzeitig abgebrochen werden. Folgende Gründe können dazu führen:

- Allergie / Unverträglichkeit der Studienmedikamente
- Fehlende / unregelmäßige Wahrnehmung der geplanten Termine

In diesem Fall oder wenn Sie die Studie von sich aus abbrechen, ist zu Ihrer Sicherheit eine medizinische Abschlussuntersuchung notwendig (=End of study visit), wobei wir die gleichen Daten erheben, wie wenn Sie die Studie regulär nach 12 Monaten beendet hätten (siehe oben).

5. Nutzen

Durch die Studienteilnahme ermöglichen Sie uns, wertvolle Information hinsichtlich der Wirkung von GLP-1 Analoga auf die Raucherentwöhnung und den Gewichtsverlauf nach Rauchstopp zu gewinnen. Im Idealfall erleichtert Ihnen das Studienmedikament den Rauchstopp und verhindert die oft damit verbundene Gewichtszunahme. Andererseits ist es auch möglich, dass Sie keinen persönlichen Nutzen daraus ziehen können. Langfristig könnte sich durch die Studie eine neue Therapiemöglichkeit für Personen mit Wunsch nach einem Rauchstopp etablieren.

6. Rechte

Die Studienteilnahme ist freiwillig. Falls Sie auf die Teilnahme dieser Studie verzichten, haben Sie keine Nachteile für eine weitere medizinische Betreuung zu erwarten. Das gleiche gilt, wenn Sie Ihre dazu gegebene Entscheidung zu einem späteren Zeitpunkt widerrufen. Sie können Ihr Einverständnis zur Studienteilnahme jederzeit zurückziehen, ohne Angabe von Gründen. Sie dürfen jederzeit Fragen zur Studie stellen. Wenden Sie sich dazu bitte an die Person, die am Ende dieser Studieninformation genannt ist.

7. Pflichten

Als Studien TeilnehmerIn sind Sie verpflichtet:

- sich an den Studienplan zu halten
- den Studienarzt/Studienärztin über die bisherige Einnahme von Arzneimitteln und über Ihre bisherige Krankengeschichte zu informieren
- den Studienarzt/Studienärztin über festgestellte unerwünschte Wirkungen zu informieren
- den Studienarzt/Studienärztin über neue Symptome, Beschwerden zu informieren
- den Studienarzt/Studienärztin über die gleichzeitige Behandlung und Therapien bei einem anderen Arzt und über Einnahme von Medikamenten zu informieren, auch solche die Sie selbst/ohne Rezept gekauft haben. Insbesondere sollten Sie während der Studie keine Nikotinersatzpräparate wie z.B. Kaugummi oder Pflaster benützen.

8. Risiken
Die Behandlung mit Dulaglutid (Trulicity®) ist in der Regel gut verträglich. Als unerwünschte Ereignisse von Dulaglutid (Trulicity®) können Magendarmbeschwerden auftreten wie Übelkeit (bis ca. 20% der Patienten) sowie seltener Erbrechen, Durchfall, Verstopfung, Bauchschmerzen (ca. 1-10% der Patienten). Die Beschwerden treten insbesondere zu Beginn der Behandlung auf und lassen in der Regel innerhalb weniger Tage unter fortgesetzter Behandlung nach. Sollten Sie an starken Nebenwirkungen leiden, gibt es auch die Möglichkeit, die Dosis des Studienmedikaments zu reduzieren. Unterzuckerungen sind bei Nichtdiabetiker ohne blutzuckersenkende Medikamente nicht zu erwarten. Während der gesamten Studiendauer werden rund 150ml Blut entnommen, was weniger ist als bei einer normalen Blutspende (450ml).
Magnetresonanztomographische Untersuchungen (MRT) dienen der bildlichen Darstellung des Körpers mit Hilfe von Magnetfeldern (d.h. ohne Röntgenstrahlung). Anhaltende Nebenwirkungen infolge einer Magnetresonanztomographie sind nicht bekannt. Allerdings ist es im MR-Scanner recht laut und eng. Wenn Sie sich daher in engen Räumen unwohl fühlen oder unter Klaustrophobie (Angst und Panik in engen Räumen) leiden, können Sie leider an der Substudie fMRI nicht teilnehmen. Personen, die schwanger sind oder stillen, die Metallteile oder medizinische Implantate (z.B. Pumpen oder Herzschrittmacher) tragen, dürfen nicht in MR-Geräten untersucht werden. Teilen Sie dies bitte in jedem Fall dem behandelnden Arzt mit. Bitte füllen Sie das Ihnen ausgehändigte Formular wahrheitsgetreu aus und Fragen Sie bei Unklarheiten das Studienpersonal.

Für gebärfähige Frauen
Da die Auswirkungen von Dulaglutid (Trulicity®) auf den Fötus (das ungeborene Kind) nicht bekannt sind, werden wir bei Studienteilnehmerinnen in gebärfähigem Alter vor Studienbeginn einen Schwangerschaftstest durchführen. Frauen mit einem positiven Schwangerschaftstest werden von der Studie ausgeschlossen. Ebenso ausgeschlossen sind Frauen, welche innerhalb der nächsten drei Monaten schwanger werden wollen oder aktuell stillen. Während der Studie ist eine sichere (doppelte) Verhütungsmethode (z.B. hormonale Methode [Pille, Spirale], kombiniert mit einer mechanischen Methode [Präservativ, Diaphragma]) anzuwenden. Teilnehmerinnen, die während der Studie schwanger werden, müssen Ihren Studienarzt umgehend informieren. In diesem Fall werden Sie gebeten, Angaben über den Verlauf und den Ausgang der Schwangerschaft zu machen. Der Prüfarzt wird mit Ihnen das weitere Vorgehen besprechen.

9. Andere Behandlungsmöglichkeiten
Sie müssen bei dieser Studie nicht teilnehmen. Wenn Sie nicht mitmachen, können Sie auch lediglich in der Rauchstopp-Sprechstunde teilnehmen, wo Sie die bislang übliche Standardbehandlung mit Verhaltenstherapie und Einnahme von Vareniclin (Champix®) erhalten. Ebenso können Sie selbstständig ohne ärztliche Unterstützung versuchen, mit dem Rauchen aufzuhören.

10. Ergebnisse aus der Studie
Der Studienarzt wird Sie während der Studie über alle neuen Erkenntnisse informieren, die den Nutzen der Studie oder Ihre Sicherheit und somit Ihr Einwilligung zur Teilnahme an der Studie beeinflussen können. Sie werden die Information mündlich und schriftlich erhalten. Bei Zufallsbefunden, die bei Ihnen zur Verhinderung, Feststellung und Behandlung bestehender oder künftig zu erwartender Krankheiten beitragen können, werden Sie informiert. Wenn Sie nicht informiert werden wollen (sog. Recht auf Nicht-Wissen), sprechen Sie bitte mit Ihrem Prüfarzt. Dies gilt ebenso für Zufallsbefunde, welche im MRI des Schädels entdeckt werden könnten.

11. Vertraulichkeit der Daten und Proben
Für diese Studie werden Ihre persönlichen und medizinischen Daten erfasst. Nur sehr wenige Fachpersonen werden Ihre unverschlüsselten Daten sehen, und zwar ausschliesslich, um Aufgaben im Rahmen der Studie zu erfüllen. Bei der Datenerhebung zu Studienzwecken werden die Daten

verschlüsselt. Verschlüsselung bedeutet, dass alle Bezugsdaten, die Sie identifizieren könnten (Name, Geburtsdatum), gelöscht und durch einen Schlüssel ersetzt werden. Diejenigen Personen, die den Schlüssel nicht kennen, können daher keine Rückschlüsse auf Ihre Person ziehen. Die Schlüssel-Liste bleibt immer in der Institution/dem Spital. Alle Personen, die Einsicht in Ihre Daten haben, unterliegen der Schweigepflicht. Alle Vorgaben des Datenschutzes werden eingehalten und wir werden Ihren Namen weder in einer Publikation noch im Internet öffentlich machen. Sie als teilnehmende Person haben jederzeit das Recht auf Einsicht in Ihre Daten.
Die Daten/Proben werden vor Ort in der Biobank Departement Endokrinologie (DepEndo) gelagert. Es ist möglich, dass Ihre Daten und Proben für andere Untersuchungen zu einem späteren Zeitpunkt weiter verwendet werden oder später an eine andere Datenbank/Biobank in der Schweiz oder ins Ausland für noch nicht näher definierte Untersuchungen (Weiterverwendung) versandt und verwendet werden. Diese andere Datenbank/Biobank muss die gleichen Standards einhalten wie die Datenbank/ Biobank zu dieser Studie. Für diese Weiterverwendung bitten wir Sie, ganz am Ende dieses Dokuments eine weitere Einwilligungserklärung zu unterzeichnen.
Möglicherweise wird diese Studie durch die zuständige Ethikkommission, die Arzneimittelbehörde Swissmedic oder durch die Institution, die die Studie veranlasst hat, überprüft. Der Prüfarzt muss eventuell Ihre persönlichen und medizinischen Daten für solche Kontrollen offenlegen. Ebenso kann es sein, dass bei Schäden ausnahmsweise auch ein Vertreter der Versicherung Ihre Daten ansehen muss. Alle Personen müssen absolute Vertraulichkeit wahren.
Es ist möglich, dass Ihr nachbehandelnder Arzt kontaktiert wird, um Auskunft über Ihren Gesundheitszustand zu geben.

12. Rücktritt
Sie können jederzeit aus der Studie aussteigen, wenn Sie dies wünschen. Die medizinischen Daten, die wir bis dahin erhoben haben und das biologische Material (Blutproben, Gewebe etc.) von Ihnen werden wir trotzdem auswerten, weil sonst die ganze Studie ihren Wert verlieren würde. Danach werden wir Ihre Daten und Ihr Material anonymisieren, d.h. wir werden endgültig Ihren Namen darauf löschen. Niemand wird danach mehr erfahren können, dass die Daten und das Material von Ihnen stammen.

13. Entschädigung
Das Studienmedikament wird ihnen gratis zur Verfügung gestellt Sie erhalten bei abgeschlossener Studie eine Aufwandsentschädigung.. Die Konsultationen im Rahmen der Rauchstopp-Sprechstunde werden von ihrer Krankenkasse übernommen. Insgesamt entstehen durch die Studie weder für sie noch für ihre Krankenkasse zusätzliche Kosten. Für die fMRI Untersuchung
erhalten Sie jeweils sFr. 50.- als Aufwandsentschädigung. Die Gesamtsumme von sFr. 100.- wird Ihnen nach dem zweiten fMRI-Termin ausgehändigt.

14. Haftung
Das Universitätsspital Basel ersetzt Ihnen Schäden, die Sie gegebenenfalls im Rahmen des klinischen Versuchs erleiden. Zu diesem Zweck hat das Universitätsspital Basel zu Ihren Gunsten eine Versicherung bei Helvetia Versicherungen, Dufourstrasse 40, 9001 St. Gallen abgeschlossen. Stellen Sie während oder nach dem klinischen Versuch gesundheitliche Probleme oder andere Schäden fest, so wenden Sie sich bitte an die verantwortliche Prüferin (Prof. Dr. M. Christ-Crain). Sie wird für Sie die notwendigen Schritte einleiten.

15. Finanzierung
Die Studie wird hauptsächlich durch das Universitätsspital Basel und die Universität Basel gesponsert.

16. Kontaktpersonen

Bei Unklarheiten, unerwarteten oder unerwünschten Ereignissen, die während der Studie oder nach deren Abschluss auftreten, können Sie sich jederzeit an die untenstehenden Kontaktpersonen wenden:

Dr. med. Bettina Winzeler, Universitätsspital Basel, +41 61 556 50 75

Notfall-Nummer (24 Stunden): Zentrale Universitätsspital: +41 61 265 25 25, Dr. med. B. Winzeler oder Dienstarzt Endokrinologie / Diabetologie verlangen.

Sponsor: Prof. Dr. med. M. Christ-Crain, Universitätsspital Basel, Petersgraben 4 , CH-4031 Basel, Telefon: +41 61 328 70 80

17. Glossar (erklärungsbedürftige Begriffe)

- Was heisst „Placebo“?
  Manche Menschen, die ein Medikament bekommen, werden nicht durch das Medikament gesund, sondern erfahren alleine durch die die Zuwendung und Fürsorge des Arztes eine Besserung. Das kann man daran erkennen, dass es ihnen besser geht, selbst wenn sie ein sogenanntes Scheinmedikament bekommen. Dieses Scheinmedikament sieht aus wie ein echtes Medikament und ist auch gleich verpackt. Tatsächlich ist aber in diesem Scheinmedikament gar kein Wirkstoff. Man nennt es „Placebo“. Wir behandeln in unserer Studie einen Teil der Teilnehmenden mit dem richtigen Medikament (mit dem Wirkstoff) und den anderen Teil mit einem solchen Placebo (ohne Wirkstoff). Dadurch können wir besser abschätzen, wie gut das Medikament tatsächlich wirkt oder ob die Besserung nur eintritt, weil die Menschen Zuwendung und Fürsorge erhalten.

- Was heisst „randomisiert“?
  Bei vielen Studien werden zwei oder mehrere unterschiedliche Arten der Behandlung verglichen. Wir zum Beispiel vergleichen ein echtes Medikament mit einem Placebo, weshalb wir zwei Gruppen von Teilnehmenden bilden. „Randomisieren“ bedeutet dann, dass ausgelost wird, wer in welche Gruppe kommt. Es also Zufall, ob man das echte Medikament erhält oder das Placebo.

- Was heisst „doppelblind"?
  Eine Studie zu "verblinden" dient dazu, bessere und genauere Ergebnisse zu erhalten. "Doppelblind" ist eine Studie dann, wenn weder die Teilnehmenden noch die Forschenden wissen, ob ein Studienteilnehmer das echte Medikament oder das Placebo erhält. Nur die unabhängige Person, die diese Zuordnung ausgelost hat, weiss, wer was erhält. Wenn der Test zu Ende ist, wird die "Verblindung" aufgelöst. In einem Notfall kann die "Verblindung" jederzeit auch früher aufgehoben werden. Der Grund für eine Verblindung ist, dass eine Person ganz anders auf den Körper achtet, wenn sie weiss, dass sie das echte Medikament erhält. Dies kann dazu führen, dass die Wirkung des echten Medikaments im Vergleich zum Placebo überschätzt wird.

- Was heisst "doppelblind randomisierter, Placebo-kontrollierter klinischer Versuch"?
  Bei der Studie wird untersucht, wie gut das neue Medikament wirkt. Dazu werden die Teilnehmenden in zwei verschiedene Gruppen eingeteilt: die Teilnehmenden der einen Gruppe erhalten das Medikament (Dulaglutid [Trulicity®]), die anderen das Placebo (Scheinmedikament). So kann man sehen, ob das neue Medikament (in unserer Studie Dulaglutid [Trulicity®]) besser wirkt, als die bisherige Standardtherapie (Verhaltenstherapie und Vareniclin [Champix®]).

Einwilligungserklärung
Schriftliche Einwilligungserklärung zur Teilnahme an der Studie

- Bitte lesen Sie dieses Formular sorgfältig durch.
- Bitte fragen Sie, wenn Sie etwas nicht verstehen oder wissen möchten.

**Nummer der Studie:**	2017-00286
**Titel der Studie:**	The SKIP Study - Smoking cessation facilitated by glucagon-like peptide-1 (GLP-1) analogues – a randomized, double-blind, placebo-controlled trial<br>- Substudy fMRI<br><br>Die SKIP-Studie - Rauchstopp durch GLP-1 Analoga – eine randomisierte, doppelblinde, Placebo-kontrollierte Studie<br>- Substudie fMRI
**Verantwortliche Institution:**	Prof. Dr. med. Mirjam Christ-Crain, Universitätsspital Basel, Petersgraben 4, 4031 Basel Telefon: +41 61 556 50 75, +41 61 328 70 80
**Ort der Durchführung:**	Universitätsspital Basel
**Leiterin der Studie am Studienort:**	Prof. Dr. med. Mirjam Christ-Crain, Dr. B. Winzeler

Prüfperson: Name/ Vorname:
Studienteilnehmer: Name/ Vorname:
Geburtsdatum:
☐ männlich ☐ weiblich

- Ich wurde von der unterzeichnenden Prüfperson mündlich und schriftlich über den Zweck, den Ablauf der Studie mit Dulaglutid (Trulicity®), über die zu erwartenden Wirkungen, über mögliche Vor- und Nachteile sowie über eventuelle Risiken informiert.
- Ich nehme an dieser Studie freiwillig teil. Ich kann jederzeit und ohne Angabe von Gründen meine Zustimmung zur Teilnahme widerrufen, ohne dass ich deswegen Nachteile bei der weiteren medizinischen Betreuung erleide. Die bis zum Rücktritt erhobenen Daten und Proben werden für die Auswertung der Studie verwendet.
- Ich hatte genügend Zeit, meine Entscheidung zu treffen.
- Meine Fragen im Zusammenhang mit der Teilnahme an dieser Studie sind mir zufriedenstellend beantwortet worden. Ich kann die schriftliche Studieninformation behalten und erhalte eine Kopie meiner schriftlichen Einwilligungserklärung. Ich akzeptiere den Inhalt der zur oben genannten Studie abgegebenen schriftlichen Studieninformation.
- Ich wurde über mögliche andere Behandlungen aufgeklärt.
- Ich bin einverstanden, dass die zuständigen Fachleute des Sponsors, der zuständigen Ethikkommission und der Arzneimittelbehörde Swissmedic zu Prüf- und Kontrollzwecken in meine unverschlüsselten Daten Einsicht nehmen dürfen, jedoch unter strikter Einhaltung der Vertraulichkeit.
- Bei Studienergebnissen, die direkt meine Gesundheit betreffen, werde ich informiert. Wenn ich das nicht wünsche, informiere ich meinen Prüfarzt.
- Ich weiss, dass meine gesundheitsbezogenen und persönlichen Daten und Proben nur in verschlüsselter Form zu Forschungszwecken für diese Studie weitergegeben werden können.
- Ich bin darüber informiert, dass eine Versicherung Schäden deckt, die auf das Forschungsprojekt zurückzuführen sind.
- Ich bin mir bewusst, dass die in der Teilnehmerinformation genannten Pflichten während der Studie einzuhalten sind. Im Interesse meiner Gesundheit kann mich der Leiter / die Leiterin jederzeit von der Studie ausschliessen.
- Ich bin einverstanden, dass der Prüfarzt, falls ich ausserhalb des Prüfzentrums weiter behandelt werde, die behandelnden Ärzte kontaktieren darf, um für die Studie relevante Nachbehandlungsdaten zu erfragen.
- Ich bin einverstanden, dass der Hausarzt über meine Studienteilnahme informiert wird: ja ☐ nein ☐

Ort, Datum	***Unterschrift des Studienteilnehmers***

Bestätigung der Prüfperson: Hiermit bestätige ich, dass ich dem Teilnehmer Wesen, Bedeutung und Tragweite der Studie erläutert habe. Ich versichere, alle im Zusammenhang mit dieser Studie stehenden Verpflichtungen gemäss dem geltenden Recht zu erfüllen. Sollte ich zu irgendeinem Zeitpunkt während der Durchführung der Studie von Aspekten erfahren, welche die Bereitschaft des Teilnehmers zur Teilnahme an der Studie beeinflussen könnten, werde ich ihn umgehend darüber informieren.

Ort, Datum	***Unterschrift der Prüfperson***

Einwilligungserklärung für Weiterverwendung von Daten und biologischem Material

Teilnehmerin/Teilnehmer:
Name und Vorname in Druckbuchstaben:
Geburtsdatum:

☐ weiblich ☐ männlich

Ich erlaube, dass meine Daten und Proben aus dieser Studie für die medizinische Forschung weiter verwendet werden dürfen. Die Proben werden in einer Biobank (Biobank Departement Endokrinologie) gelagert und für zukünftige, noch nicht näher definierte Forschungsprojekte auf unbestimmte Zeitdauer verwendet. Diese Einwilligung gilt unbegrenzt.

Ich habe verstanden, dass die Proben verschlüsselt sind und der Schüssel sicher aufbewahrt wird. Die Daten und Proben können im In- und Ausland an andere Daten- und Biobanken zur Analyse gesendet werden, wenn diese dieselben Standards wie in der Schweiz einhalten. Alle rechtlichen Vorgaben zum Datenschutz werden eingehalten.

Ich entscheide freiwillig und kann diesen Entscheid zu jedem Zeitpunkt wieder zurücknehmen. Wenn ich zurück trete, werden meine Daten anonymisiert und meine Proben vernichtet. Ich informiere lediglich meinen Prüfarzt und muss diesen Entscheid nicht begründen.

Normalerweise werden alle Daten und Proben gesamthaft ausgewertet und die Ergebnisse zusammenfassend publiziert. Sollte sich ein für meine Gesundheit wichtiges Ergebnis ergeben, ist es möglich, dass ich über meinen Prüfarzt kontaktiert werde. Wenn ich das nicht wünsche, teile ich es meinem Prüfarzt mit.

Wenn Ergebnisse aus den Daten und Proben kommerzialisiert werden, habe ich keinen Anspruch auf Anteil an der kommerziellen Nutzung.

Ort, Datum	Unterschrift Teilnehmerin/ Teilnehmer

Bestätigung des Prüfarztes/der Prüfperson: Hiermit bestätige ich, dass ich dieser Teilnehmerin/ diesem Teilnehmer Wesen, Bedeutung und Tragweite der Weiterverwendung von Proben und/ oder genetischen Daten erläutert habe.

Ort, Datum	Name und Vorname der informierenden Prüfärztin/ des informierenden Prüfarztes/ der informierenden Prüfperson in Druckbuchstaben
	Unterschrift der Studienärztin/des Studienarztes/ der Prüfperson
